# Concise synthesis of (*R*)-reticuline and (+)-salutaridine by combining early-stage organic synthesis and late-stage biocatalysis†

**DOI:** 10.1039/d3sc02304d

**Published:** 2023-08-24

**Authors:** Emmanuel Cigan, Jakob Pletz, Sarah A. Berger, Bettina Hierzberger, Michael Grilec-Zlamal, Alexander Steiner, Isabel Oroz-Guinea, Wolfgang Kroutil

**Affiliations:** a Institute of Chemistry, University of Graz, NAWI Graz, BioTechMed Graz Heinrichstrasse 28/II 8010 Graz Austria Wolfgang.Kroutil@uni-graz.at; b Field of Excellence BioHealth, University of Graz 8010 Graz Austria

## Abstract

Efficient access to the morphinan scaffold remains a major challenge in both synthetic chemistry and biotechnology. Here, a biomimetic chemo-enzymatic strategy to synthesize the natural promorphinan intermediate (+)-salutaridine is demonstrated. By combining early-stage organic synthesis with enzymatic asymmetric key step transformations, the prochiral natural intermediate 1,2-dehydroreticuline was prepared and subsequently stereoselectively reduced by the enzyme 1,2-dehydroreticuline reductase obtaining (*R*)-reticuline in high ee and yield (>99% ee, up to quant. conversion, 92% isol. yield). In the final step, membrane-bound salutaridine synthase was used to perform the selective *ortho-para* phenol coupling to give (+)-salutaridine. The synthetic route shows the potential of combining early-stage advanced organic chemistry to minimize protecting group techniques with late-stage multi-step biocatalysis to provide an unprecedented access to the medicinally important compound class of promorphinans.

## Abstract 2

Designing an alternative access to the morphinan scaffold by taking advantage of biocatalysis for asymmetric C=N reduction and oxidative C–C bond formation.

## Graphical abstract

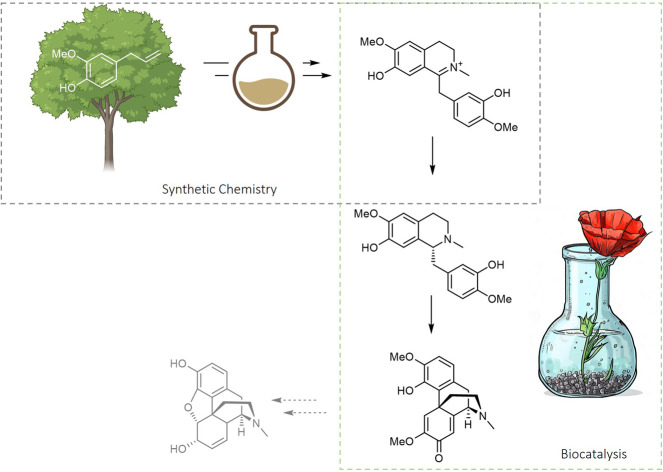

## 1 Introduction

**Scheme 1:**
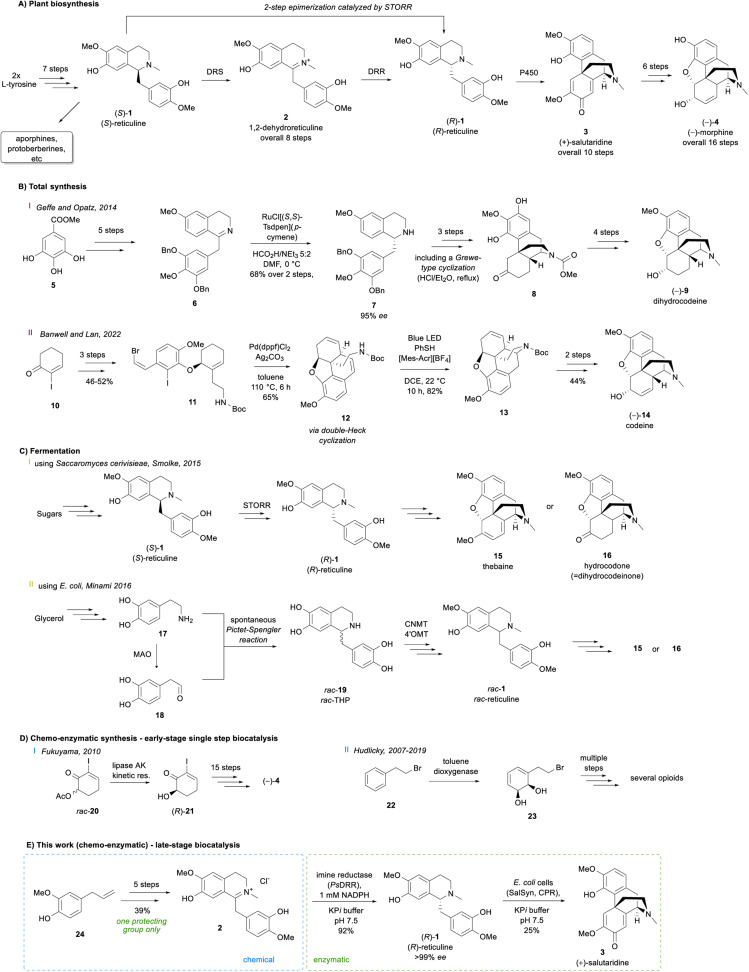
Overview of selected pathways to opioids including the natural pathway in plants (A), synthetic approaches involving chemical methods only (B), biotechnological exploitation of the natural pathways or selected parts of them *via* fermentation (C), chemo-enzymatic routes with early-stage single step biocatalysis (D) and a chemo-enzymatic route with late-stage biocatalysis (E).

Alkaloids occur in all kinds of organisms and represent highly important natural products especially for medicine.^1,2^ One major subcategory are benzylisoquinoline alkaloids (BIAs), which consist of more than 2500 known members.^3^ To construct the central metabolic BIA (*S*)-reticuline, (*S*)-1, nature starts from two l-tyrosine molecules to obtain within seven steps, (*S*)-1 (Scheme 1A), which is then diversified towards a plethora of complex alkaloids like morphinans, protoberberines, aporphines and others, showing a wide spectrum of different biological activities.^4^ For an efficient synthesis of the morphinan subclass including the prominent alkaloid morphine (−)-4, the formation of stereochemically inverted (*R*)-reticuline (*R*)-1 and subsequently (+)-salutaridine (+)-3, the first alkaloid in the sequence with promorphinan structure, is crucial.

Organic synthesis routes to natural opioid structures containing the morphinan architecture were extensively investigated,^5–14^ counting at least 40 published routes. The first approach considered as practical was published in 1980, accessing racemic dihydrocodeinone *rac*-16 by a Grewe-type cyclization as the key transformation.^7^ Since then, impressive progress has been made in the field exploiting a large variety of different strategies. For instance, a stereoselective high-yielding route published in 2014 comprised the synthesis of (−)-dihydrocodeine 9 in 13 steps and 24% overall yield (Scheme 1BI). The route starts from a commercial building block 5 and combines a ruthenium-catalyzed hydrogenation and a subsequent Grewe-type cyclization.^10^ As the most recent success in the field of organic synthesis, a seven-step strategy towards (−)-codeine 14 needs to be mentioned, which uses a double-Heck cyclization reaction coupled with a subsequent photo-redox hydroamination to build the target molecule's pentacyclic scaffold (Scheme 1BII).^14^

Although the recent synthetic progress to produce opiates is impressive, there are still challenges which need to be addressed like the use of several protecting groups and sophisticated transition metal catalysts lacking perfect stereoselectivities. Remarkably, the plant's crucial building block (*R*)-reticuline (*R*)-1 does not serve as an intermediate in any of the chemical approaches since there are no strategies in organic synthesis to directly and selectively transform (*R*)-1 to the tetracyclic promorphinan scaffold of (+)-salutaridine (+)-3 except for protected reticuline derivatives.^15–19^ Additionally, biochemical and biotechnological approaches have been investigated regarding their potential to produce morphinans/opioids in recent years.

In 2015, the complete metabolic pathway towards opioids was expressed in yeast to enable the opioid formation from sugar and can be considered as a proof of principle (Scheme 1CI).^20^ Even if the morphinans thebaine 15 and hydrocodone 16 were indeed produced, the obtained product titers were 6.4 and 0.5 μg L^−1^, respectively, rendering the process economically uncompetitive by several orders of magnitude.

Only one year later, the same target compounds were reported to be successfully synthesized in *E. coli*, resulting in higher titers (2.1 mg L^−1^ and 0.36 mg L^−1^ for thebaine and hydrocodone, respectively, Scheme 1CII).^21^ In both cases, the main bottlenecks proved to be the efficient formation of (*R*)-reticuline (*R*)-1 and the following C–C bond forming phenol coupling reaction. In the case of the approach using yeast, the natural fusion protein STORR was employed to form (*R*)-1 from its (*S*)-enantiomer, whereas an alternative strategy was established for the *E. coli* system. Since the native reticuline epimerase STORR showed insufficient activity when expressed in bacteria (*E. coli*), its use was circumvented by the racemic production of tetrahydropapaveroline (THP, *rac*-19). The latter was formed by a spontaneous Pictet–Spengler reaction of dopamine 17 and its monoamine oxidase catalyzed oxidation product 18. Two methyltransferases converted *rac*-19 to *rac*-reticuline *rac*-1, of which only the (*R*)-enantiomer was accepted by the next enzyme SalSyn reducing the atom efficiency by 50% (Scheme 1CII).^21^

The third possible methodology for the synthesis of morphinans is to combine chemo- and enzymatic strategies. This approach has successfully been used for the synthesis of a variety of natural products, synthetic APIs or interesting building blocks thereof and has experienced a lot of attention in the last years.^22–34^ The concepts developed for opioid structures are exclusively based on early stage enzymatic transformations to access basic chiral synthons followed by a sophisticated chemical synthetic sequence over numerous steps (Scheme 1DI and II).^35–45^ The biotransformations used involved either a broadly exploited toluene dioxygenase (for dihydroxylation) or a lipase (for a kinetic resolution).

In contrast, herein, we report a late-stage biocatalysis strategy in a 6–7-step chemo-enzymatic sequence for the synthesis of (*R*)-reticuline (*R*)-1 and its promorphinan product (+)-salutaridine (+)-3 (Scheme 1E), respectively, which are crucial intermediates for the morphinan/opioid synthesis. (+)-Salutaridine (+)-3 is of interest as it is a partial agonist at the GABA/benzodiazepine receptor complex,^46^ a partial MOR agonist,^47^ and also due to its use as anti-HBV agent.^48^ The herein used strategy includes the transformation of a lignin-derived feed stock (24) towards the natural intermediate 1,2-dehydroreticuline 2. The target molecule's chiral information was introduced by involving two final enzymatic steps.

## 2 Results and discussion

**Scheme 2:**
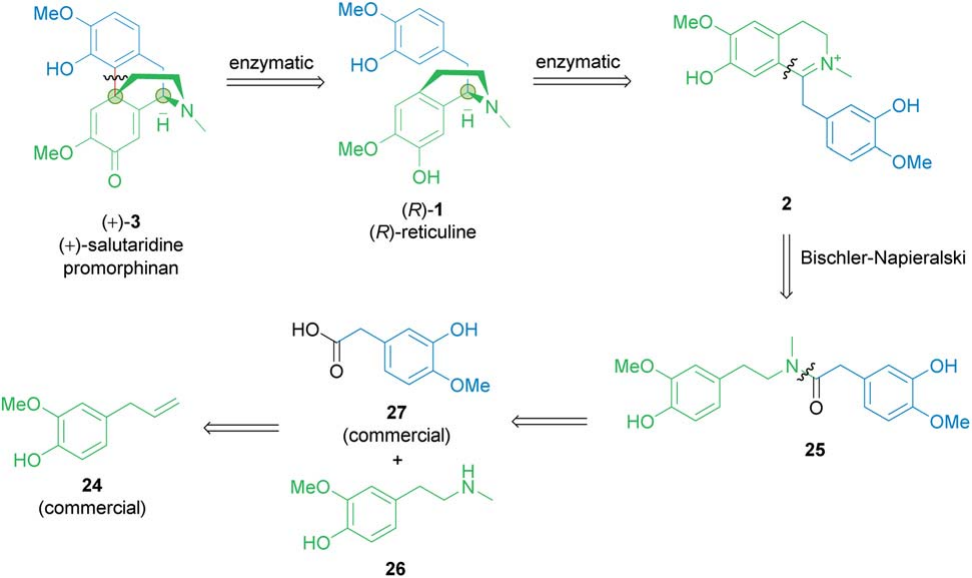
Retrosynthetic analysis of the promorphinan salutaridine 3. Green circles: chiral centers formed.

Considering established organic synthetic methodology as well as the potential from biocatalysis including non-established bio-reactions, a retrosynthetic analysis of the promorphinan (+)-salutaridine (+)-3 was performed to develop a potential efficient stereoselective route (Scheme 2). Thereby the natural intermediate 1,2-dehydroreticuline 2 was recognized as a possible prochiral intermediate, which may be transformed towards the tetracyclic structure of (+)-3 in two enzymatic steps. Consequently, we first focused on a short synthetic high-yielding route towards iminium ion 2 with the aim to minimize the use of protecting groups. Ideally, 2 would be formed *via* a Bischler–Napieralski reaction from amide 25, whereby the phenolic hydroxy groups may present a challenge. Amide 25 would be obtainable from the commercial carboxylic acid 27 and the secondary phenethylamine 26.

### Synthesis of amine building block 26

In an earlier publication,^49^ vanillin was used as starting material to produce the *O*-benzyl-protected derivative of phenethylamine building block 26 in a 5-step-synthetic sequence in 31% overall yield requiring a protection strategy. The protecting group was needed for the subsequent synthetic steps, including the condensation with another protected building block to give the corresponding amide.

**Scheme 3:**
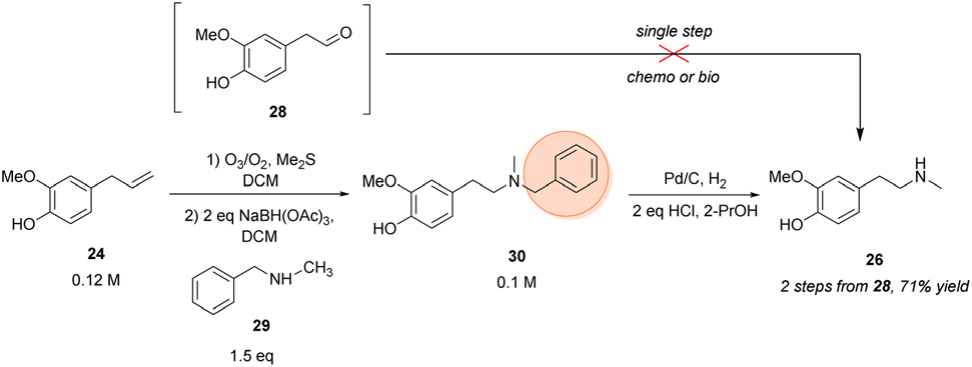
Chemical synthesis of the *N*-methylated phenethylamine building block 26. Orange circle: protecting group.

Here, eugenol 24 is suggested as an alternative feed stock containing already the required guaiacol unit of the target molecule (Scheme 3). Eugenol is a natural compound and a cheap starting material due to its high abundance in cloves and also to its accessibility as a lignin degradation product.^50,51^ The envisioned synthetic approach towards 26 involved the oxidative alkene cleavage to yield homovanillin 28 which may then be *N*-methylated.

For the formation of 28 from 24, different strategies were investigated, like using dihydroxylation/periodate cleavage. However, the latter method did not lead to the desired product since the reaction conditions seemed to be too harsh for selective cleavage of the corresponding diol intermediate (data not shown). Conversely, the ozonolysis of eugenol followed by a reductive workup was shown in literature^52^ and proved to be a suitable strategy for the transformation towards the respective aldehyde, accessing 28 in 74% isolated yield.

For the next step towards the secondary amine building block 26, numerous protocols were evaluated for the amination of aldehyde 28 whereby the theoretical most straight forward strategy would be the direct methylamination using MeNH_2_ as the amine donor. However, this approach was limited by overalkylation of the desired phenethylamine building block due to its increasing nucleophilicity after amination and was not overcome by numerous strategies (for details, see ESI†). Consequently, as direct methylamination seemed to be unfeasible, protecting group chemistry had to be considered.

Thus, the amination was performed using benzyl methylamine 29 in combination with NaBH(OAc)_3_ which led to >99% conversion of the starting material within 90 min and resulted in 79–84% isolated yield of the protected tertiary amine 30 after column purification. The subsequent deprotection was performed by hydrogenolysis over palladium on charcoal. After optimization, the hydrochloride of target amine 26 was isolated in 90% yield without the necessity of a column purification, just a filtration step was required to remove the Pd catalyst.

### Preparation of the natural intermediate 2

**Scheme 4:**
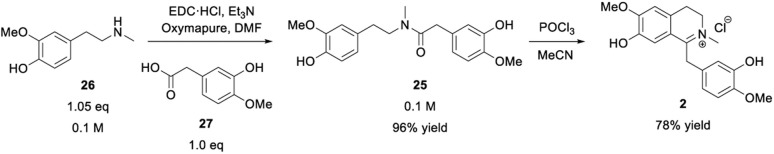
Protecting group-free amide coupling of building block 26 with acetic acid derivative 27 followed by a Bischler–Napieralski-type cyclization of amide 25 towards iminium ion 2.

Once the phenethylamine building block 26 was accessible, it was coupled with the commercial acetic acid derivative 27 containing another guaiacol subunit to achieve amide formation leading to 25 (Scheme 4).

The amide formation was performed employing the carbodiimide EDC·HCl for acid activation combined with either HOBt·H_2_O or Oxyma Pure as additives. The latter proved to be the method of choice for amide couplings.^53^ The *O*-acylisourea species initially formed between EDC and the carboxylic acid 27 may potentially rearrange through an intramolecular acyl-transfer towards the corresponding *N*-acylurea species. This rearrangement is irreversible and would lead to a dead end of the reaction. Therefore, the rate of addition of the limiting carboxylic acid to the other reaction components was controlled, ensuring a large excess of amine donor after activation of the acid. The auxiliary reagents (HOBt·H_2_O or Oxyma Pure) increased the overall rate of the process^54^ and were used in a stoichiometric amount. Both, HOBt·H_2_O and Oxyma Pure worked similarly well in terms of reactivity but the latter seemed to be the best choice since the oxime reagent has a less polar character and is thermally more stable than the benzotriazole derivative HOBt. Furthermore, HOBt showed a similar *R*_f_-value to the product, which rendered the separation by column chromatography more problematic. Consequently, 25 was obtained as the sole product in up to 96% isolated yield after flash chromatography. The next step was the cyclization of amide 25 towards iminium ion 2 which was performed *via* a Bischler–Napieralski reaction using POCl_3_ as reagent (Scheme 4). To our satisfaction, the cyclization was successful with the unprotected amide 25, which was not shown before in the synthesis of similarly complex compounds (*e.g.*, alkaloids) as the phenolic groups had always been protected.^49^ By the use of POCl_3_ and acetonitrile under reflux, complete consumption of the starting material was observed after four hours, leading to the formation of the target iminium ion 2 as the major product. The subsequent workup for 2 was simple, including solvent and POCl_3_-removal under reduced pressure. Subsequently, the product was purified by flash chromatography and after the treatment with aq. HCl, compound 2 was finally obtained in 78% yield as a canary yellow, fine-crystalline salt. Thus, the natural intermediate 1,2-dehydroreticuline 2 was obtained in a short 5-step organic synthesis approach in 39% overall yield starting from the lignin-derived feed stock eugenol 24.

### Enzymatic reduction towards (*R*)-reticuline (*R*)-1

While numerous strategies have been developed for the synthesis of *rac*-1 and (*S*)-1, including organic (asymmetric) synthesis,^55,56^ as well as *via* fermentation^57–67^ or a biocatalytic deracemisation approach,^68^ reports on the synthesis of the mirror image (*R*)-reticuline (*R*)-1 are scarce.^49,69,70^ Until now, only approaches involving classic organic synthesis have been described. Here, a biocatalytic late-stage strategy was implemented to introduce chirality exploiting the reductive domain of the natural fusion protein CYP82Y2 (STORR) from the opium poppy (*Papaver somniferum*).^71^ STORR, consisting of two domains, epimerizes in nature (*S*)-reticuline (*S*)-1 to (*R*)-1*via* an oxidation–reduction sequence: a cytochrome P450 oxidase domain catalyzes the amine oxidation, leading to the prochiral iminium ion, and a C-terminal aldo-keto reductase domain performs its nicotinamide-dependent asymmetric reduction.^71^

**Scheme 5:**
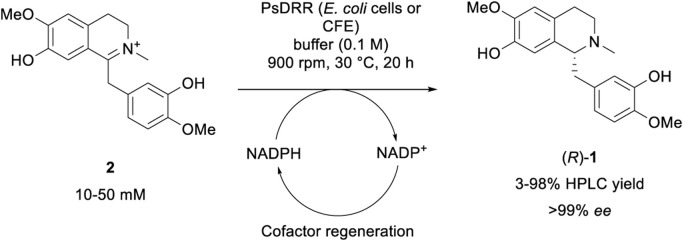
Biotransformation of iminium ion 2 towards optically pure amine target (*R*)-1 employing PsDRR; CFE = cell-free extract.

**Fig. 1:**
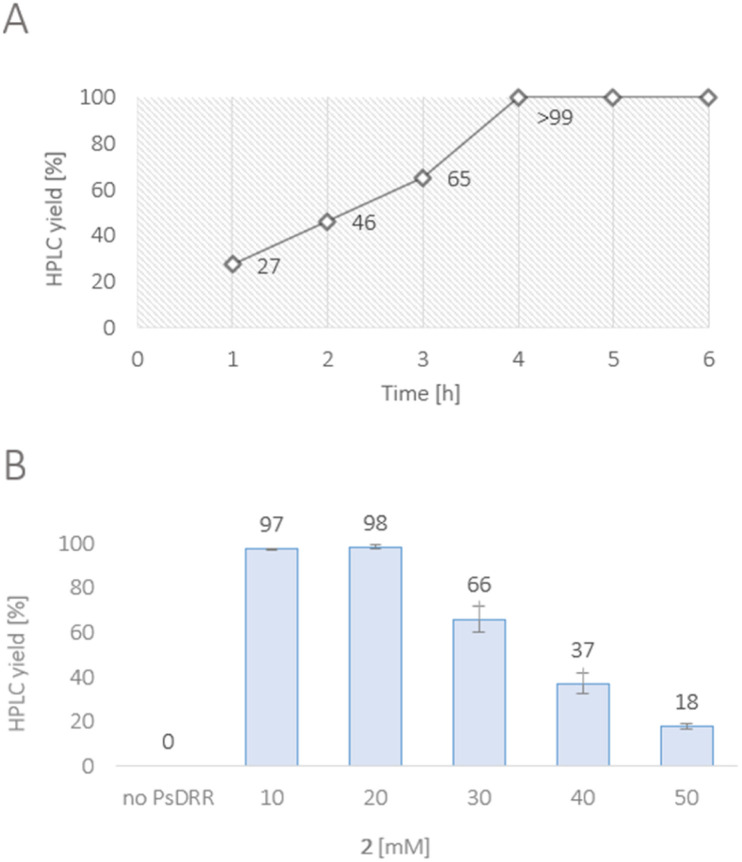
(A) Time course of the reduction of 2 towards (*R*)-1. Reaction conditions: substrate 2 (10 mM), PsDRR preparation (lyophilized cells, 0.06 U mL^−1^), NADP^+^ (1 mM), d-glucose (50 mM), GDH (24 U mL^−1^), KPi buffer (100 mM, pH 7.0), 30 °C, 900 rpm. (B) Substrate loading study of the reduction of 2 towards (*R*)-1. Reaction conditions: substrate 2 (10–50 mM), PsDRR preparation (lyophilized cells, 0.06 U mL^−1^), NADP^+^ (1 mM), d-glucose (50 mM), GDH (4.5 U mL^−1^), KPi buffer (100 mM, pH 7.0), 30 °C, 900 rpm, 20 h. The experiment was performed in triplicates.

The gene encoding for the reductase domain (PsDRR, hereinafter also called 1,2-dehydroreticuline reductase) was expressed in *E. coli* ArcticExpress (DE3) cells. The enzyme proved to be suitable in biocatalytic transformations both as whole lyophilized cells, as well as lyophilized cell-free extract using a cofactor regeneration system to recycle NADPH (Scheme 5). Initial reaction time studies showed that the conversion of substrate 2 at 10 mM was completed within 3–4 hours yielding the target amine (*R*)-1 in optical purity (>99% ee, Fig. 1A). Testing the productivity of the reaction revealed that HPLC yields of 98% could be reached for substrate concentrations up to 20 mM (6.6 g L^−1^, Fig. 1B) and in some cases even 30 mM (9.9 g L^−1^) of 2 (*e.g.*, Fig. S10†). Remarkably, the investigation of the pH optimum and the buffer reaction medium revealed two maxima within the tested pH range (see ESI, Fig. S13†). Apart from the optimum at pH 8.0–8.5 using Tris–HCl buffer, another optimum was found for the pH range 9.5–10.0 (glycine–NaOH buffer). This observation may be explained by the target molecule's electronic properties, namely the presence of acidic phenolic groups paired with a nitrogen functionality. Consequently, a minimum at pH 9.0 may derive from a large population containing the net charge 0. Studying the influence of organic cosolvents revealed that 10% DMSO increased the HPLC yield by 35% compared to the cosolvent-free reaction (see ESI, Fig. S14†). All other cosolvents tested at 2% v/v were well tolerated and did not influence the reaction outcome. Moreover, the PsDRR-catalyzed reaction was well compatible with different cofactor regeneration systems: in addition to the initially used GDH system, also other cofactor recycling systems were investigated, whereby ADH/2-propanol as well as phosphite dehydrogenase (PtDH) showed product formation exceeding 95% HPLC yield within 20 hours at 30 mM substrate concentration. Formate dehydrogenase was less efficient at the conditions used (see ESI, Fig. S8†). Actually, when using whole lyophilized cells, the addition of GDH for cofactor recycling can be skipped as the endogenous GDHs present in *E. coli* seemed to be sufficient for cofactor recycling when just d-glucose was added at substrate concentrations up to 30 mM (see ESI, Fig. S9†).

After the initial optimization, the reaction was evaluated also with higher substrate loadings up to 100 mM (see ESI, Fig. S15 and S17†), which did not lead to increased yields compared to the starting conditions. The results indicated a substrate inhibition which triggered to investigate the enzymatic reaction in a fed-batch approach. Since it was known that the quantitative conversion of 10 mM of 2 is completed within 3–4 h (Fig. 1A), the substrate was added stepwise increasing the concentration by 10 mM (referred to the final conc.) every 4 h. Interestingly, the control reaction containing only 10 mM substrate loading resulted in the same absolute product formation (see ESI, Fig. S19†) as the fed-batch reaction which was performed with 80 mM substrate loading in total. This observation strongly suggests that the enzyme lost its functionality already after the first loading with 10 mM, indicating additionally a reduced stability of the enzyme under the conditions employed.

The enzymatic reduction of 2 was finally performed on a 0.5 mmol scale to show the enzyme's applicability for the chemo-enzymatic route. The biotransformation was performed with whole lyophilized cells using optimized conditions but without cofactor regeneration enzyme (GDH) and in the absence of cosolvent to ease product isolation. Using this protocol, 92% (162 mg) of optically pure (*R*)-reticuline was isolated, demonstrating the applicability of this recently described biocatalyst for the chemo-enzymatic synthesis.

### Enzymatic phenol coupling towards (+)-salutaridine (+)-3

**Scheme 6:**
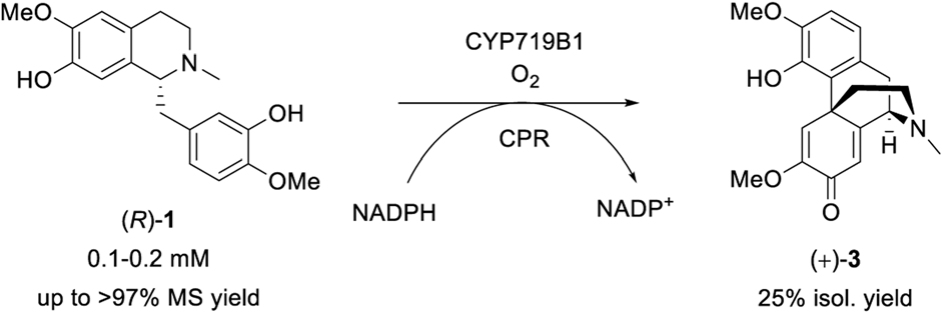
Biotransformation of (*R*)-1 towards the promorphinan target (+)-salutaridine (+)-3 employing SalSyn (CYP719B1); reaction scale: 0.5–200 mL.

The final investigated step was the regioselective phenol coupling of (*R*)-1 to set up the promorphinan structure of (+)-salutaridine (+)-3, which represents the key step in morphinan biosynthesis and would be the ideal step for biomimetic organic synthesis (Scheme 6).^72^

This specific synthetic problem was addressed for the first time in organic synthesis already in the 1960's and has been extensively investigated ever since.^73^ Among all the developed phenol coupling strategies, including heavy metal oxidants,^73–76^ hypervalent iodine reagents,^77–79^ electrochemical set-ups,^18,19,80–86^ and transition metal catalysts,^12,13^ the conversion of (*R*)-1 towards (+)-3 either failed, resulted in low yields or had to be performed using sophisticated protecting and blocking group strategies to achieve high yields. For instance, a palladium-catalyzed arene coupling to access the promorphinan framework required an activating (bromo) and protecting group and was applied in a total synthesis of (−)-codeine 14, (−)-oxycodone, (−)-naloxone, and (−)-naltrexone.^12,13^

The main reasons for the limitations can be ascribed to the methods' lack of regioselectivity and the presence of the reactive phenolic functional groups. In comparison, the enzyme salutaridine synthase (SalSyn, CYP719B1) is known to catalyze exclusively the intramolecular *ortho-para* phenol coupling of (*R*)-1 towards (+)-3 in certain poppy species like *Papaver somniferum* and has recently been shown to be active in yeast- and bacteria-based fermentative multi-enzyme systems.^20,21^ However, since this plant CYP is membrane-associated and was reported to be unstable,^72^ literature lacks examples in which SalSyn was used in preparative biocatalytic strategies.

**Fig. 2:**
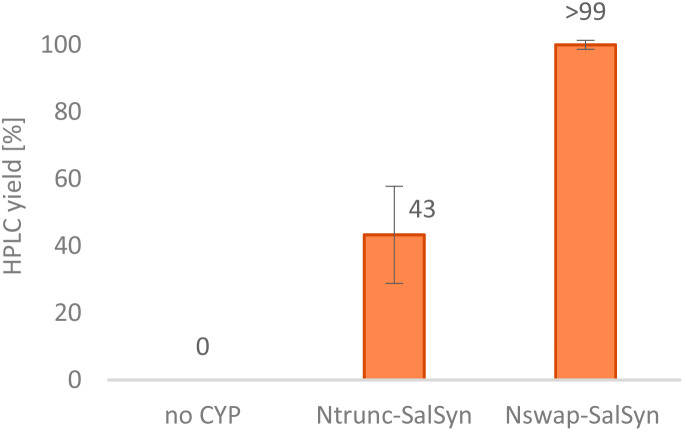
Conversion of (*R*)-1 towards (+)-salutaridine (+)-3 by two different SalSyn constructs. Reaction conditions: substrate *rac*-1 (0.2 mM), coexpressed SalSyn (CYP719B1) and AtR2 preparation (suspended *E. coli* cells, OD_600_ ≈ 0.22), NADPH (0.05 mM), d-glucose (50 mM), 0.2% DMSO v/v, KPi buffer (100 mM, pH 7.5), 30 °C, 180 rpm, 20 h; reaction scale: 0.5 mL. The experiment was performed in triplicates.

Our idea was to employ CYP719B1 as a single catalyst to circumvent the limitation of fermentative processes (such as side activities, limited substrate/product concentration, *etc.*) and ultimately incorporate the enzyme in an efficient chemo-enzymatic synthesis route. In 2016, SalSyn was reported to be active when expressed in *E. coli*, however only if the native transmembrane domain was removed.^21^ In our study, we used such a N-terminal-truncated SalSyn candidate (Ntrunc-SalSyn) and prepared a second construct, named here Nswap-SalSyn, containing a polar charged N-terminal sequence (KKTSSKGR) inspired by previous general P450 optimization studies.^87^ These enzymes were coexpressed with the compatible reductase AtR2 from *Arabidopsis thaliana* in *E. coli*, and used as resting cells for biotransformations performed in buffer. All experiments in which lysate was used rather than whole cells did not show phenol coupling activity, which was assumed to be due to the low enzyme stability reported for this specific enzyme.^72^ For the performed biotransformations, *rac*-1 was used at a concentration of 0.2 mM [*i.e.*, 0.1 mM (*R*)-1] and product formation was followed *via* HPLC-UV and HPLC-MS. The N-terminal truncated P450 led to a HPLC yield of 43% whereas the more polar construct Nswap-SalSyn showed quantitative product formation for the substrate loading used, corresponding to a ≥2.3-fold increase in yield (Fig. 2). Finally, the biotransformation was upscaled to 12.8 mg (optically pure starting material), which resulted in the isolation of 25% isolated yield of (+)-3. Studying different volume to surface ratios for the reaction (reaction volume to size of reaction flask) showed the crucial impact of oxygen for the enzymatic reaction (see ESI†).

Even if the substrate concentration was still low and the enzyme requires further optimization towards higher productivity by improving stability and expression, SalSyn (CYP719B1) seems to be suitable for the use in a chemo-enzymatic strategy, as shown in the herein described approach.

## 3 Conclusions

Here, a synthetic route towards (*R*)-reticuline (*R*)-1 and subsequently to the crucial natural promorphinan (+)-3 [(+)-salutaridine] on the route to morphine/thebaine is proposed requiring only a single protecting group. As a prochiral precursor for (*R*)-1, the corresponding iminium ion 2 was identified. While nature requires eight steps to construct intermediate 2 from two l-tyrosine molecules, herein 2 was obtained by advanced organic synthesis starting from renewable feedstock eugenol 24 within 5 steps, thereby outperforming biosynthesis regarding its reaction step count in an overall high-yielding sequence (39% isol. yield). The Bischler–Napieralski reaction was used as the last step of this synthetic sequence. Importantly, this cyclization step was achieved avoiding protecting groups, which had not been shown for the synthesis of similar targets before, probably due to the reactive phenol hydroxy groups. By stereoselectively reducing the prochiral intermediate 2 to (*R*)-1 using a dehydroreticuline reductase (PsDRR), the detour nature is actually taking by making first (*S*)-1 and inverting this structure *via* an oxidation–reduction sequence, was circumvented. This means a reduction of step count in comparison to nature. Thus, the preparation of the BIA (*R*)-reticuline (*R*)-1 was achieved in a six-step chemo-enzymatic sequence with unmatched efficiency, accessing the target amine in 36% isolated yield and with >99% ee. To access (+)-salutaridine (+)-3 from (*R*)-1*via* a protecting group-free phenol coupling, for which organic synthesis lacks an equivalent,^88^ a novel variant of the salutaridine synthase possessing a polar charged N-terminal sequence proved to be suited best giving (+)-3 with >99% conversion albeit at low substrate concentration [0.2 mM *rac*-1 >97% MS yield at 0.19 mM optically pure substrate (upscale)]. It is clear, that especially this step needs further improvement. Interestingly, the synthetic sequence presented is currently not suggested by any retrosynthetic program, most likely due to a lack of integrated biocatalysis or design of novel options (like Bischler–Napieralski without protecting groups at phenol).

The sequence described leads on the one hand to the natural product (+)-3, representing the morphinan gateway, and on the other hand also serves as a synthetic platform for natural intermediates (*R*)-1 and 2. This is in contrast to total synthesis routes which usually access the final targets only. Thereby, either multiple protecting and blocking groups were required, especially for the crucial phenol coupling in biomimetic organic synthesis,^12,13^ or fundamentally different concepts were used.^14^ Furthermore, even if opioid targets are accessible by engineered organisms, final titers are far from representing competitive numbers. The only reported example of a morphinan precursor, namely (*S*)-1, which was shown to be accessed in high concentrations was produced by a yeast platform.^65^ However, (*R*)-1 is still not accessible by microbial approaches since the epimerization of (*S*)-1 by STORR was shown to be the bottleneck of fermentative opioid production systems. Thus, the developed chemo-enzymatic approach also overcomes limits which were encountered in fermentative systems.

In summary, the here presented synthetic route combining early-stage chemical synthesis requiring only one protecting group with late-stage biocatalysis introducing the chirality and performing a regioselective *ortho-para* phenol coupling may serve as a base for further development to access opiates in a chemo-multi-enzymatic sequence. Additionally, potential derivatives of (+)-3 with potential bioactivity would now be accessible by starting from alternative precursors.

## Data availability

All experimental and characterization data including HPLC traces and NMR spectra are available in the ESI.†

## Author contributions

EC, JP, SB, BH, MGZ, AS, IOG conducted the experiments. EC prepared the ESI.† EC and JP contributed to the formal analysis of the results and reviewed the manuscript. EC wrote the first draft of the manuscript. WK conceptualized and supervised the research, did project administration, edited the draft and allocated funding.

## Conflicts of interest

EC, JP and WK are inventors of the patent ‘Verfahren zur Herstellung von Corytuberin und Salutaridin’.

## Supplementary Material

SC-014-D3SC02304D-s001
