# Supplementary material for: Concise synthesis of (R)-reticuline and (+)-salutaridine by combining early-stage organic synthesis and late-stage biocatalysis
Source: Chem Sci. 2023 Aug 24;14(36):9863–71. doi: 10.1039/d3sc02304d (PMC10510765; doi:10.1039/d3sc02304d)
Supplement: SC-014-D3SC02304D-s001 [file SC-014-D3SC02304D-s001.pdf]

Electronic Supplementary Material (ESI) for Chemical Science.
This journal is © The Royal Society of Chemistry 2023

Supporting Information

Concise synthesis of (*R*)-reticuline and (+)-salutaridine by combining early-stage organic synthesis and late-stage biocatalysis

Emmanuel Cigan, Jakob Pletz, Sarah Berger, Bettina Hierzberger, Alexander Steiner, Michael Grilec-Zlamal, Isabel Oroz-Guinea, Wolfgang Kroutil

Contents

1 General 2
2 Chemical synthesis of iminium salt **2** 4
2-(4-Hydroxy-3-methoxyphenyl)acetaldehyde, homovanillin (**28**) 4
4-(2-(Benzyl(methyl)amino)ethyl)-2-methoxyphenol (**30**) 5
2-Methoxy-4-(2-(methylamino)ethyl)phenol hydrochloride (**26**·HCl) 5
N-(4-Hydroxy-3-methoxyphenethyl)-2-(3-hydroxy-4-methoxyphenyl)-*N*-methylacetamide (**25**) 6
7-Hydroxy-1-(3-hydroxy-4-methoxybenzyl)-6-methoxy-2-methyl-3,4-dihydroisoquinolin-2-ium chloride (**2**).. 7
1-(3-Hydroxy-4-methoxybenzyl)-6-methoxy-2-methyl-1,2,3,4-tetrahydroisoquinolin-7-ol (*rac*-**1**) 8
2a Amination of homovanillin 9
3 Protein expression 10
3 Enzymatic reduction of dehydroreticuline (**2**) to (*R*)-reticuline [(*R*)-**1**] 14
(*R*)-1-(3-hydroxy-4-methoxybenzyl)-6-methoxy-2-methyl-1,2,3,4-tetrahydroisoquinolin-7-ol [(*R*)-**1**] 20
4 Enzymatic C-C phenol coupling of (*R*)-reticuline [(*R*)-**1**] to salutaridine (**3**) 22
5 Experimental spectra 27
6 Protein sequences 39
7 DNA sequences 40
8 Primers 42
9 References 42

1 General

All chemicals were purchased from Sigma Aldrich, Acros Organics, Alfa Aesar or ABCR and were used as received. All solvents were purchased from Roth. Solvents labeled with abs. were absolutely dry. Laboratory grade water was used from the in-house equipment (desalination apparatus). Gene strings were ordered from Invitrogen by Thermo Fisher Scientific and Plasmids containing genes were ordered from Biocat GmbH and General Biosystems. PsDRR was a gift from Prof. P. Facchini. Glucose dehydrogenase (GDH) was obtained as a crude cell-free extract from DSM (activity for $NADP^+$ of batches GDH.002, GDH.003 and GDH.004: 2.2-2.4 U mg^{-1}) and used as received. Lb-ADH was available in-house and published earlier from our group.[1] FDH was available as in-house preparation based on a published sequence (NADP-FDH-Variant D222Q/H224N from *Pseudomonas* sp. 101, see expression part).[2-3] PtDH was prepared based on a published sequence (see expression part).[4-5]

All equipment was used under standard conditions unless stated otherwise.

<u>TLC</u>

Reactions were monitored *via* TLC. As stationary phase silica gel plates from Merck were used (silica gel 60 F254, 20x20 cm). Detection was achieved by UV (254 nm) and CAM blue. For making the detection faster a heat gun was used to dry the silica gel plates.

TLC solutions:

CAM blue: 90 mL H_2O (distilled), 2.5 g ammonium molybdate tetrahydrate, 1 g ammonium cerium(IV) sulfate dehydrate, stirring until dissolved and 10 mL H_2SO_4 added.

<u>Flash chromatography</u>

Column chromatography was performed on silica gel 60 from Merck with particle sizes 40-63 µm. A 30- to 100-fold excess of silica gel was used with respect to the mass of dry crude product, depending on the separation problem. The crude material was dissolved in an appropriate solvent and either loaded as the liquid material or subsequently adsorbed on the 2.5-fold excess of Celite™. For the Celite preparation, the solvent was removed under vacuum and the adsorbed crude material was dried under oil pump vacuum. The dimension of the column was adjusted to the required amount of silica gel and formed a pad between 16 and 30 cm. In general, the silica gel was mixed with the eluent and charged into the column before equilibration by forcing an appropriate amount of eluent through by over-pressure. Subsequently, the dissolved or adsorbed crude material was loaded onto the top of the silica gel and the mobile phase was forced through the column by pressure exerted by a rubber bulb pump. The volume of each collected fraction was adjusted between 20% and 30% of the silica gel volume, according to the separation problem.

Instrumentation

^{1}H- and ^{13}C- and spectra were recorded on a Bruker AVANCE III 300 spectrometer (^{1}H: 300.36 MHz; ^{13}C: 75.53 MHz) with an autosampler. Chemical shifts were referenced to the residual proton and carbon signal of the deuterated solvent [$CDCl_3$: δ = 7.260 ppm (^{1}H), 77.160 ppm (^{13}C); MeOH-d_4: δ = 3.31 (^{1}H), 49.00 ppm (^{13}C)]. Chemical shifts δ are given in ppm (parts per million) and coupling constants J in Hz (Hertz). Signal multiplicities are abbreviated as s (singlet), bs (broad singlet), d (doublet), dd (doublet of doublet), t (triplet) and m (multiplet). Additionally, quaternary carbon atoms are designated as C_q and aromatic carbon atoms bearing a hydrogen as CH_{arom}. 1D-NMR spectra (APT) as well as 2D-NMR spectra (HH-COSY, HSQC) were recorded for the identification and confirmation of the structure. Deuterated solvents for nuclear resonance spectroscopy were purchased from Roth, Armar Chemicals and Euriso-top®.

GC-MS measurements were performed on an Agilent 7890A GC system, equipped with an Agilent 5975C mass-selective detector (EI 70 eV) and a HP-5-MS column (30 m x 0.25 mm x 0.25 µm film) using He at a flow rate of 0.5 mL min^{-1}. Temperature program: 100 °C, hold 0.5 min, 10 °C min^{-1} 300 °C, hold 2 min, inlet temperature 250 °C.

Melting points were determined on an "MPD350.BM2.5" melting point apparatus from Gallenkamp with an integrated microscopical support. They were measured in open capillary tubes and were not corrected.

IR-spectra were recorded neat on a Bruker Alpha-P (ATR) instrument.

High resolution mass spectra were recorded on an Agilent 6230 TOF LC/MS using ESI method (positive or negative mode, nozzle voltage 2.0 kV) with coupled Agilent 1260 Infinity Series HPLC.

Liquid chromatography

Low resolution mass spectra (LC-MS) were recorded on an Agilent Technologies 6120 Quadrupole LC/MS detector in combination with an Agilent Technologies 1260 Infinity HPLC system, equipped with a Kinetex 2.6 µm C-18 100 Å column (50x4.6 mm) or a Luna 5 µm C-18 100 Å column (250x4.6 mm). Water/acetonitrile (+0.1 vol-% of formic acid) was used as eluent.

HPLC-UV analysis was carried out on a Shimadzu HPLC system, equipped with a Luna 5 µm C-18 100 Å column (250x4.6 mm) [DGU-20A (degasser), LC-20A (pump), SIL-20A (autosampler), CTO-20AC (column oven), SPD-M20A (detector), CBM-20AC (controller)] with water, acetonitrile and methanol as eluent. As additive either TFA or DEA [0.1% (v/v)] or none of them were used.

Determination of reticuline and salutaridine: HPLC yields were determined by HPLC on an achiral reversed phase C18 stationary phase (Luna). Eluent: buffer (30 mM $HCOONH_4$, pH 2.8)/methanol/acetonitrile = 67/30/3 (isocratic); flow rate: 0.5 mL min^{-1}; column temperature: 25 °C; detection wavelength: 280 nm.

2 Chemical synthesis of iminium salt **2**

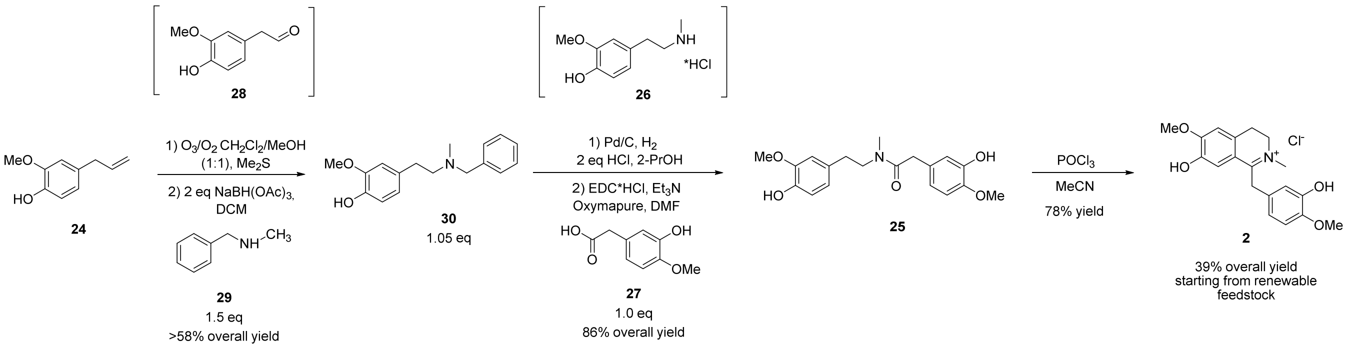

Scheme S1: Synthetic route towards natural intermediate **2**.

2-(4-Hydroxy-3-methoxyphenyl)acetaldehyde, homovanillin (**28**)

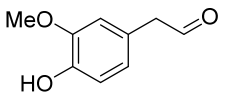

A three-necked round-bottom flask (500 mL) with a magnetic stirring bar was charged with eugenol (**24**, 5.00 g, 30.5 mmol, 1 eq) followed by CH_2Cl_2 (125 mL) and MeOH (125 mL). The yellow solution was cooled to -10 °C with a cryostat in an EtOH bath and an O_3/O_2 mixture was passed through the solution through a sintered glass frit for 6 h. After complete consumption of the starting material (TLC, EtOAc/cyclohexane = 3:1, R_f = 0.54), nitrogen was passed through the solution for 10 min in order to remove the excess of ozone. The reactive intermediate was quenched by the addition of Me_2S (11.2 mL, 153 mmol, 5 eq) at -10 °C and after 15 min the mixture was allowed to warm to room temperature. After complete removal of the hydroperoxyacetal (TLC, cyclohexane/EtOAc = 1:1, R_f = 0.36) the mixture was reduced under vacuum. The product was purified by column chromatography [size: 30 x 6 cm, cyclohexane/EtOAc =3:1 (v/v)].

Yield: 3.77 g (22.7 mmol, 74%) pale-yellow solid.

R_f = 0.44 (cyclohexane/ EtOAc = 1:1 (v/v)) (254 nm, CAM: dark blue).

IR (ATR) = 3341, 3062, 3005, 2988, 2955, 2867, 2748, 1702, 1599, 1510, 1463, 1451, 1429, 1363, 1261, 1233, 1205, 1175, 1148, 1123, 1048, 1025, 1008, 916, 852, 808, 792, 710, 621, 561, 506, 467 cm^{-1}.

mp: 43 - 45 °C.

^{1}H-NMR (300.13 MHz, $CDCl_3$): δ = 9.71 (t, J = 2.5 Hz, 1H, CHO), 6.91 (d, J = 8.0 Hz, 1H, CH_{arom}), 6.72 (dd, J = 8.0, 2.0 Hz, 1H, CH_{arom}), 6.68 (d, J = 2.0 Hz, 1H, CH_{arom}), 5.64 (bs, 1H, OH), 3.88 (s, 3H, OCH_3), 3.60 (d, J = 2.5 Hz, 2H, CH_2).

^{13}C-NMR (75.47 MHz, $CDCl_3$): δ = 199.7 (CHO), 147.0 (C_q), 145.2 (C_q), 123.5 (C_q), 122.7 (CH_{arom}), 115.0 (CH_{arom}), 112.1 (CH_{arom}), 56.1 (OCH_3), 50.3 (CH_2).

GC-MS (ACHIRAL-MSD): t_R = 8.086 min, m/z (rel. int.) = 166 [M^+] (25), 138 (9), 137 (100), 122 (18), 94 (11), 77 (6), 66 (5), 51 (6).

HR-MS (HPLC-ESI): *m/z* [M-H]$^-$ calcd for $C_9H_{10}O_3$: 165.0557; found: 165.0557.

4-(2-(Benzyl(methyl)amino)ethyl)-2-methoxyphenol (**30**)

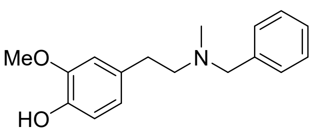

A dry 500 mL round-bottom flask with magnetic stirring bar was charged with 2-(4-hydroxy-3-methoxyphenyl)acetaldehyde (**28**) (4.00 g, 24.1 mmol, 1 eq) and CH_2Cl_2 (240 mL). The pale-yellow solution was cooled to 0 °C with an ice bath and *N*-methyl-1-phenylmethanamine (**29**) (4.80 mL, 36.1 mmol, 1.5 eq) was slowly added. $NaBH(OAc)_3$ (10.4 g, 48.1 mmol, 2 eq) was added in portions over a period of 3 min, the flask was sealed with a septum and the mixture was left stirring in the thawing ice bath. After 150 min another portion of $NaBH(OAc)_3$ (2.60 g, 0.5 eq) was added at room temperature. Complete conversion of the starting material was detected after a total of 18 h by TLC and the reaction mixture was quenched by the addition of saturated aqueous $NaHCO_3$ solution and solid $NaHCO_3$. The solvents were removed on the rotary evaporator (bath temperature: 50 °C) and the product was purified by two consecutive column chromatographies (1st column: 17 x 4.8 cm, CH_2Cl_2/acetone = 1:1, 2nd column: 16 x 4.8 cm, CH_2Cl_2/acetone = 2:1).

Yield: 5.15 g (19.0 mmol, 79%) light-brown solid.

R_f = 0.73 ($CHCl_3$/MeOH/NH_4OH = 4:1:1% (v/v/v)) (254 nm, CAM: dark blue).

IR (ATR) = 2995, 2968, 2931, 2857, 2827, 2757, 2671, 2544, 2478, 1603, 1588, 1513, 1497, 1464, 1455, 1430, 1417, 1382, 1343, 1311, 1268, 1231, 1195, 1152, 1125, 1081, 1033, 1004, 987, 961, 924, 860, 843, 824, 778, 755, 728, 700, 639, 567, 522, 487,463, 426, 412 cm^{-1}.

mp: 94 - 96 °C.

^{1}H-NMR (300.13 MHz, $CDCl_3$): δ = 7.37 – 7.21 (m, 5H, CH_{arom}), 6.82 (d, *J* = 7.8 Hz, 1H, CH_{arom}), 6.72 – 6.63 (m, 2H, CH_{arom}), 3.86 (s, 3H, OCH_3), 3.62 (s, 2H, $ArCH_2N$), 2.86 – 2.75 (m, 2H, CH_2), 2.73 – 2.61 (m, 2H, CH_2), 2.32 (s, 3H, NCH_3).

^{13}C-NMR (75.47 MHz, $CDCl_3$): δ = 146.6 (C_q), 144.1 (C_q), 138.1 (C_q), 132.1 (C_q), 129.4 (CH_{arom}), 128.5 (CH_{arom}), 127.4 (CH_{arom}), 121.4 (CH_{arom}), 114.4 (CH_{arom}), 111.5 (CH_{arom}), 62.1 (CH_2), 59.3 (CH_2), 56.0 (OCH_3), 42.0 (NCH_3), 33.4 (CH_2).

HR-MS (HPLC-ESI): *m/z* [M+H]$^+$ calcd for $C_{17}H_{21}NO_2$: 272.1645; found: 272.1645.

2-Methoxy-4-(2-(methylamino)ethyl)phenol hydrochloride (**26**·HCl)

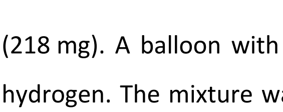

A 1000 mL round-bottom flask with magnetic stirring bar was charged with 4-(2-(benzyl(methyl)amino)ethyl)-2-methoxyphenol (**30**) (4.35 g, 16.0 mmol, 1 eq), 2-propanol (160 mL), 12 M aqueous HCl (2.7 mL, 32.0 mmol, 2 eq) and Pd/C 10% (218 mg). A balloon with hydrogen was fitted on top of the flask and it was evacuated and backfilled with hydrogen. The mixture was vigorously stirred at 22 °C until complete conversion of the starting material was detected after 3 d. The mixture was filtrated through a pad of Celite (6 cm) and the pad was rinsed consecutively

with 2-propanol (500 mL), 2-propanol/H_2O (1:1, 300 mL) and MeOH (400 mL). The solvents were removed on the rotary evaporator and the product was dried under vacuum.

Yield: 3.13 g (14.4 mmol, 90%) brown solid.

R_f = 0.35 ($CHCl_3$/MeOH/NH_4OH = 2:1:1% (v/v/v)) (254 nm, CAM: blue).

IR (ATR) = 3113, 3066, 3021, 2962, 2935, 2846, 2758, 2423, 1602, 1586, 1523, 1455, 1432, 1404, 1373, 1310, 1277, 1263, 1235, 1218, 1205, 1156, 1132, 1112, 1046, 1032, 996, 958, 910, 861, 824, 794, 728, 677, 628, 567, 548, 517, 454 cm^{-1}.

mp: 145 - 147 °C.

^{1}H-NMR (300.13 MHz, MeOD-d$_4$): δ = 6.92 (d, J = 1.9 Hz, 1H, CH_{arom}), 6.82 (d, J = 8.0 Hz, 1H, CH_{arom}), 6.77 (dd, J_1 = 8.0 Hz, J_2 = 1.9 Hz, 1H, CH_{arom}), 3.86 (s, 3H, OCH_3), 3.22 (t, J = 7.7 Hz, 2H, CH_2), 2.92 (t, J = 7.4 Hz, 2H, CH_2), 2.70 (s, 3H, NCH_3).

^{13}C-NMR (75.47 MHz, MeOD-d$_4$): δ = 149.3 (C_q), 146.8 (C_q), 128.8 (C_q), 122.3 (CH_{arom}), 116.6 (CH_{arom}), 113.4 (CH_{arom}), 56.5 (OCH_3), 51.8 (CH_2), 33.8 (NCH_3), 32.9 (CH_2).

HR-MS (HPLC-ESI): m/z $[M+H]^+$ calcd for $C_{10}H_{15}NO_2$: 182.1176; found: 182.1177.

Note: The deprotected amine salt can be directly used for the following amide coupling step without the need of a column chromatography purification.

N-(4-Hydroxy-3-methoxyphenethyl)-2-(3-hydroxy-4-methoxyphenyl)-*N*-methylacetamide (**25**)

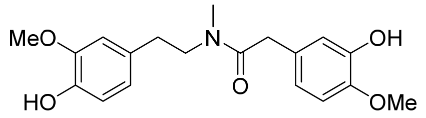

A dry 250 mL round-bottom flask with magnetic stirring bar was charged with 2-methoxy-4-(2-(methylamino)ethyl)phenol hydrochloride (**26**·HCl) (560 mg, 2.57 mmol, 1.05 eq), DMF (20 mL) and Oxyma Pure (350 mg, 2.44 mmol, 1 eq). The yellow solution was cooled to 0 °C with an ice bath and Et_3N (0.34 mL, 2.44 mmol, 1 eq) was added followed by EDC·HCl (520 mg, 2.69 mmol, 1.1 eq). To the vigorously stirred solution 2-(3-hydroxy-4-methoxyphenyl)acetic acid (**27**) (460 mg, 2.44 mmol, 1 eq) in DMF (4 mL) was added dropwise over a period of 3 h via syringe pump. Complete conversion of the starting material was detected after 19 h (TLC) and the solvent was removed under vacuum. The residual solid material was dissolved in EtOAc (70 mL) and washed with aq. HCl solution (3 x 50 mL). The combined aqueous washing solution was neutralized with $NaHCO_3$ solution and extracted with EtOAc (3 x 70 mL). The organic phases were combined with the previous organic phases, concentrated under vacuum and purified by column chromatography [26 x 3.3 cm, EtOAc/cyclohexane= 2:1 to 4:1 (v/v)]. The product was dried under oil pump vacuum (Note: strong foaming!).

Yield: 807 mg (2.34 mmol, 96%) colorless, glassy solid.

R_f = 0.20 (EtOAc/cyclohexane = 3:1 (v/v)) (254 nm, CAM: dark blue).

IR (ATR) = 3247, 3008, 2936, 2839, 1614, 1588, 1510, 1431, 1403, 1359, 1269, 1234, 1213, 1151, 1128, 1027, 965, 929, 856, 816, 787, 758, 693, 665, 615, 591, 559, 457 cm^{-1}.

mp: 41-43 °C.

The product is obtained as a mixture of rotamers (ratio rotamer 1/rotamer 2 = 1.14/1), to which NMR signals are assigned based on the peak intensities as well as the APT and HSQC spectra.

^{1}H-NMR (300.13 MHz, $CDCl_3$): δ = 6.90 – 6.48 (m, 6H, CH_{arom}), 4.94 (brs, 2H, ArOH), 3.94 – 3.77 (m, 6H, OCH_3), 3.66 –3.34 (m, 4H, CH_2), 2.97 (s, 1.4H, NCH_3, rotamer 2), 2.87 (s, 1.6H, NCH_3, rotamer 1), 2.76 (t, 1.1H, CH_2, rotamer 1), 2.63 (t, 0.9H, CH_2, rotamer 2).

rotamer 1: ^{13}C-NMR (75.47 MHz, $CDCl_3$): δ = 171.4 (C_q), 146.7 (C_q), 145.9 (C_q), 145.7 (C_q), 144.2 (C_q), 131.0 (C_q), 128.1 (C_q), 121.5 (CH_{arom}), 120.3 (CH_{arom}), 115.2 (CH_{arom}), 114.4 (CH_{arom}), 111.6 (CH_{arom}), 111.1 (CH_{arom}), 56.1 (OCH_3), 56.0 (OCH_3), 50.4 (CH_2), 40.8 (CH_2), 36.7 (NCH_3), 33.4 (CH_2).

rotamer 2: ^{13}C-NMR (75.47 MHz, $CDCl_3$): δ = 171.6 (C_q), 146.8 (C_q), 145.9 (C_q), 145.7 (C_q), 144.7 (C_q), 130.1 (C_q), 128.4 (C_q), 121.5, 120.2 (CH_{arom}), 115.2 (CH_{arom}), 114.8 (CH_{arom}), 111.5 (CH_{arom}), 111.1 (CH_{arom}), 56.2 (OCH_3), 56.1 (OCH_3), 52.5 (CH_2), 40.2 (CH_2), 34.5 (CH_2), 33.9 (NCH_3).

HR-MS (HPLC-ESI): *m/z* $[M+H]^+$ calcd for $C_{19}H_{23}NO_5$: 346.1649; found: 346.1650.

7-Hydroxy-1-(3-hydroxy-4-methoxybenzyl)-6-methoxy-2-methyl-3,4-dihydroisoquinolin-2-ium chloride (**2**)

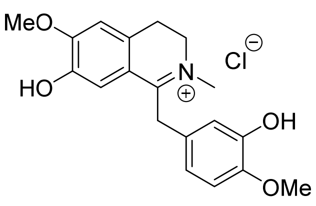

A three-necked round bottom flask (250 mL) with magnetic stirring, air condenser, pressure equilibrator and gas valve was purged with Argon for 45 min. *N*-(4-hydroxy-3-methoxyphenethyl)-2-(3-hydroxy-4-methoxyphenyl)-*N*-methylacetamide (**25**) (1.00 g, 2.90 mmol, 1 eq) in dry MeCN (30 mL) was added in an Ar counter-stream followed by $POCl_3$ (824 µL, 8.69 mmol, 3 eq). The colorless solution was heated to reflux and complete conversion of the starting material was detected after 4 h (TLC). Celite (2.5 g) was added and the solvent was removed on the rotary evaporator. The product was purified by column chromatography (size: 30 x 1.8 cm, $CHCl_3$/MeOH/TFA = 14:2:1%, 12:2:1%, 10:2:1% (v/v/v)) and a second column chromatography [size: 30 x 1.8 cm, $CHCl_3$/MeOH/TFA = 8:1:1%, 7:1:1%, 6:1:1%, 5:1:1%, 4:1:1% (v/v/v)]. The product was dissolved in MeOH, filtrated through cotton and the solvent was removed under vacuum. The product was then dissolved in distilled H_2O (50 mL), filtrated through a pad of Celite (2 cm), treated with 12 M HCl (500 µL) and was dried under vacuum.

Yield: 821 mg (2.26 mmol, 78%) bright-yellow powder.

R_f = 0.29 ($CHCl_3$/MeOH/TFA = 4:1:1% (v/v/v)) (254 nm, CAM: blue).

IR (ATR) = 3540, 3091, 2926, 2847, 2749, 1661,1624, 1603, 1570, 1524, 1435, 1382, 1341, 1301, 1239, 1210, 1187, 1151, 1131, 1093, 1042, 1016, 980, 948, 881, 867, 839, 814, 794, 761, 741, 715, 681, 647, 589, 558, 542, 482, 442, 409 cm^{-1}.

mp: 143 - 145 °C.

^{1}H-NMR (300.13 MHz, MeOD-d_4): δ = 7.39 (s, 1H, CH_{arom}), 7.05 (s, 1H, CH_{arom}), 6.89 (d, $^{3}J_{HH}$ = 8.2 Hz, 1H, CH_{arom}), 6.65 (d, $^{4}J_{HH}$ =2.2 Hz, 1H, CH_{arom}), 6.61 (dd, $^{3}J_{HH}$ = 8.2 Hz, $^{4}J_{HH}$ = 2.2 Hz, 1H, CH_{arom}), 4.42 (s, 2H, CH_2), 4.06 (t, $^{3}J_{HH}$ = 7.7 Hz, 2H, CH_2), 4.00 (s, 3H, OCH_3), 3.82 (s, 3H, OCH_3), 3.70 (s, 3H, NCH_3), 3.14 (t, $^{3}J_{HH}$ = 7.7 Hz, 2H, CH_2).

^{13}C-NMR (75.47 MHz, MeOD-d_4): δ = 176.6 (C_q), 156.9 (C_q), 148.8 (C_q), 148.6 (C_q), 147.8 (C_q), 133.5 (C_q), 126.8 (C_q), 120.9 (C_q), 120.3 (CH_{arom}), 118.0 (CH_{arom}), 116.1 (CH_{arom}), 113.5 (CH_{arom}), 111.9 (CH_{arom}), 57.0 (OCH_3), 56.4 (OCH_3), 53.9 (CH_2), 44.6 (NCH_3), 36.3 (CH_2), 26.4 (CH_2).

HR-MS (HPLC-ESI): *m/z* $[M-Cl]^+$ calcd for $C_{19}H_{22}NO_4^+$: 328.1543; found: 328.1541.

1-(3-Hydroxy-4-methoxybenzyl)-6-methoxy-2-methyl-1,2,3,4-tetrahydroisoquinolin-7-ol (*rac*-**1**)

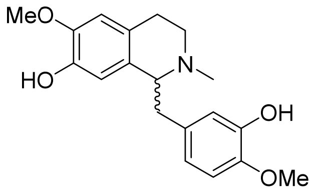

A dry 10 mL round-bottom flask with magnetic stirring bar was charged with 7-hydroxy-1-(3-hydroxy-4-methoxybenzyl)-6-methoxy-2-methyl-3,4-dihydroisoquinolin-2-ium chloride (**2**) (172 mg, 473 µmol, 1 eq) and MeOH (4 mL) and the yellow solution was cooled to 0 °C with an ice bath. $NaBH_4$ (17.9 mg, 473 µmol, 1 eq) was added to the vigorously stirred solution and complete conversion of the starting material was detected after 20 min. The solvent was removed under vacuum, the crude product was adsorbed on Celite (MeOH) and the product was purified by column chromatography (size: 18 x 1.4 cm, CH_2Cl_2/MeOH/NH_4OH = 30:2:1%).

Yield: 80.6 mg (245 µmol, 51%) pale-yellow foam.

R_f = 0.38 (CH_2Cl_2/MeOH/NH_4OH = 9:1:1%) (254 nm, CAM: dark blue).

mp: 72 - 75 °C.

The NMR data is in accordance with literature.[3]

2a Amination of homovanillin (**28**)

Although numerous common strategies were tested (i.e. an excess of substrate amine, variation of solvent, etc.), no suitable conditions were found to overcome side product formation. In general, it was possible to obtain the target compound **26** as the main product, accompanied by the tertiary amine as the major side product. In this context, it was possible to push the ratio between the target product and the overalkylated tertiary amine product up to 89% (Figure S1). However, the better this ratio became, the more additional side products appeared. For the direct amination using methylamine, the HCl salt was used rather than the free base, which led to higher yields of the target amine when MeOH was used as the solvent. Apart from the commonly used hydride sources NaBH4, NaCNBH3 and NaBH(OAc)3, also the additional use of titanium (IV) isopropoxide Ti(OiPr)4 was investigated in an alternative strategy,[6-8] however leading either to no product formation or complex product mixtures.

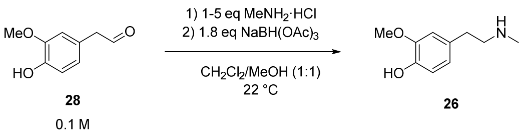

Scheme S2: Direct amination of substrate aldehyde **28** by using different amounts of $MeNH_2$·HCl.

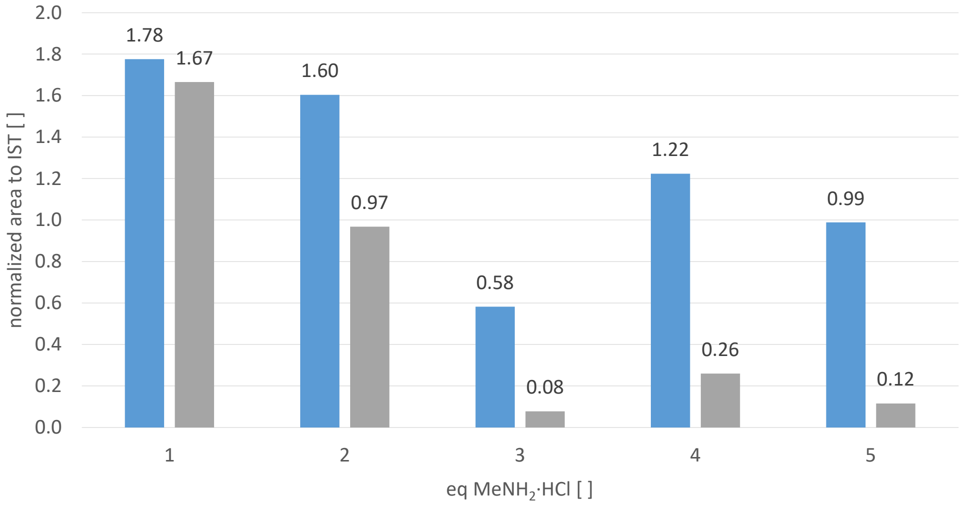

Figure S1: $MeNH_2$·HCl loading study for the direct reaction of homovanillin (**28**) to phenethylamine target **26**. Blue: target product **2**, grey: di-alkylated tertiary side product, IST: internal standard.

Overall, the direct chemical amination of homovanillin (**28**) with methylamine (base or salt) in an organic solvent led to low yields of the target product, usually accompanied by a significant number and quantity of byproducts, which consequently rendered this strategy unsuitable. As an alternative, an enzymatic methylamination in an aqueous system was evaluated. For this purpose, various in-house imine reductases were tested for the reductive amination of aldehyde **28** with methylamine in the presence of NADPH and a glucose-based cofactor regeneration system. However, the enzymatic transformation did not lead to the desired compound but to an undefined product (most likely a dimer of **28**) which only occurred in the presence of methylamine (Figure S2). Noteworthy, the structurally similar compounds phenylacetone and phenylacetaldehyde did not show this behaviour and were stable also in the presence of MeNH2 concentrations up to 300 mM.

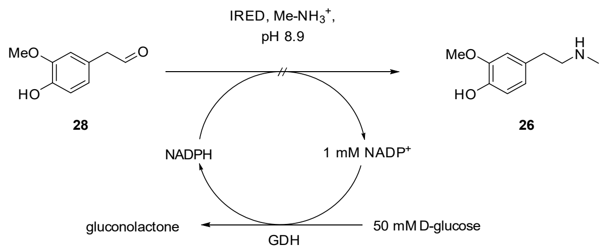

Scheme S3: Target biotransformation for the amination of the substrate aldehyde **28.**

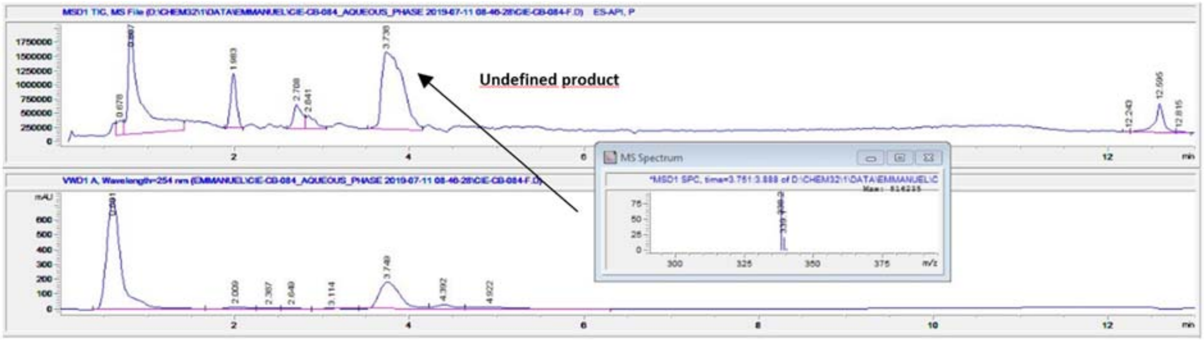

Figure S2: HPLC-MS and –UV chromatograms of the amination of the substrate aldehyde **28** using imine reductases in buffer.

3 Protein expression

<u>Expression of PsDRR</u>

Transformation into *E. coli* Arctic express(DE3) cells as the expression host

The plasmid containing the corresponding gene and chemically competent *E. coli* ArcticExpress (DE3) host cells (50 µL) were mixed in 1.5 mL microcentrifuge vials. The mixtures were incubated on ice for 30 min. Subsequently, the competent cell/DNA mixtures were shock-heated at 42 °C for 20 s. Autoclaved LB-medium (700 µL) was added to each sample followed by shaking at 37 °C, 300 rpm for 1 h. Subsequently, 100 µL of the mixture was applied onto previously prepared agar plates containing kanamycin (50 µg mL^{-1}) which were placed in an incubator at 37 °C overnight.

Preparation of overnight cultures (ONC)

50 mL plastic tubes were filled with 10 mL LB-medium and 10 µL of kanamycin stock solution (50 mg mL^{-1}) was added. A single culture from the agar plate was picked for inoculation and cultivated at 30 °C, 300 rpm overnight.

Main culture

A 1 L-baffled cultivation flask was filled with 330 mL autoclaved LB medium, supplemented with kanamycin solution (300 µL, 50 mg mL^{-1}, final conc.: 50 µg mL^{-1}) and 3.3 mL prepared overnight culture (1%). Cell growth proceeded at 30 °C, 120 rpm until OD_{600} = 1.0 was reached. Subsequently, the cultures were induced with IPTG (1 M stock, final conc.: 0.5 mM). The cell cultures were incubated at 20 °C, 120 rpm for 20-24 h.

In order to harvest the enzyme, the cultures were centrifuged (5000 rpm, 4 °C for 20 min), resuspended in distilled water and centrifuged again (5000 rpm, 4 °C for 20 min). The pellets were resuspended again in 10 mL water per gram wet pellet. For the preparation of whole lyophilized cells, the resuspension was directly flash-frozen (liquid N_2) and lyophilized. Alternatively, the cell resuspension was sonicated (1 s on, 2 s off, 40% amplitude, 5 min) and centrifuged (14000 rpm, 4 °C 20 min). For the latter case, the pellets were discarded and

the supernatant was flash-frozen (liquid N_2) and lyophilized. The obtained whole lyophilized whole cells as well as lyophilized cell free extracts (CFE) were stored at -20 °C.

Expression of FDH and PtDH

Precultures were prepared as described above using kanamycin (final conc.: 50 µg mL^{-1}).

Baffled cultivation flasks (1 L) were filled with 330 mL autoclaved LB medium, supplemented with kanamycin solution (300 µL, 50 mg mL^{-1}, final conc.: 50 µg mL^{-1}) and 3.3 mL prepared overnight culture (1%). Cell growth proceeded at 37 °C, 120 rpm until OD_{600} = 0.5-0.7 was reached. Subsequently, the cultures were induced with IPTG (1 M stock, final conc.: 1 mM). The cell cultures were incubated at 20 °C (FDH) or 25 °C (PtDH), 120 rpm for 20-24 h. In order to harvest the enzyme, the cultures were centrifuged (5000 rpm, 4 °C for 20 min), resuspended in deionized water and centrifuged again (5000 rpm, 4 °C for 20 min). The pellets were resuspended again in 10 mL water per gram wet pellet. For the preparation of whole lyophilized cells, the resuspension was directly flash-frozen (liquid N_2) and lyophilized. Alternatively, the cell resuspension was sonicated (1 s on, 2 s off, 40% amplitude, 5 min) and centrifuged (14000 rpm, 4 °C 20 min). For the latter case, the pellets were discarded and the supernatant was flash-frozen (liquid N_2) and lyophilized. The obtained lyophilized whole cells as well as lyophilized cell free extracts (CFE) were stored at -20 °C.

Preparation of CYP-CPR strains (SalSyn and AtR2)

Chemically competent *E. coli* BL21(DE3) cells were transformed with the plasmid pCDF-1b containing the AtR2 encoding gene as described above for the transformation of PsDRR in *E. coli* Arctic express(DE3), albeit using spectinomycin (100 µg mL^{-1}) as antibiotic. The transformed strain was used for the following preparation of co-expressing strains harboring AtR2 and the respective CYP.

Preparation of electrocompetent *E. coli* BL21(DE3) cells harbouring SalSyn or P450 reductase

Preparation of overnight cultures

Falcon tubes were filled with 10 mL LB-media and spectinomycin solution (10 µL, 100 mg mL^{-1}, final conc.: 100 µg mL^{-1}) was added. A single colony from an agar plate (containing AtR2) was picked for inoculation and cultivated at 37 °C, 120 rpm overnight.

Cultivation and harvesting

LB medium (500 mL) supplemented with spectinomycin (final conc.: 100 µg mL^{-1}) was inoculated with fresh ONC [1% (v/v)]. The cells were grown at 37 °C to an OD_{600} of 0.5-0.7, cooled on ice for 20 min and transferred to sterile 400 mL centrifugation vessels. All steps were kept as close to 0 °C as possible and performed under sterile conditions. The cells were centrifuged at 4800 rpm for 15 min and the supernatant was discarded. The received pellets were resuspended carefully in 500 mL of ice-cold glycerol (10%). The cells were centrifuged at 4800 rpm for 15 min and the supernatant was discarded again. The pellets were resuspended carefully in 250 mL of ice-cold glycerol (10%). Subsequently, the cells were centrifuged at 4800 rpm for 15 min and the supernatant was discarded again. The pellets were resuspended in 20 mL of ice-cold glycerol (10%), centrifuged, and resuspended in 2 mL of ice-cold glycerol (10%). The suspensions were used to make aliquots of 80 µl which were flash-frozen in liquid nitrogen and stored at -80 °C until use.

The resulting electro-competent *E. coli* BL21(DE3) cells were transformed with pET-28a plasmids containing the respective SalSyn constructs by electroporation using an electroporation apparatus (MicroPulser™, Program: Ec2). The transformed cell solutions were applied onto previously prepared agar plates containing spectinomycin and kanamycin (final conc.: 50 and 100 µg mL^{-1}, respectively), which were placed in an incubator at 37 °C overnight.

Expression of CYP-CPR strains

Precultures were prepared as described above using both spectinomycin and kanamycin (final conc.: 50 and 100 µg mL^{-1}, respectively).

Cultivation

TB medium (5 mL) in 10 mL DWP was supplemented with 5 µL kanamycin solution and 5 µL spectinomycin solution (final conc.: 50 and 100 µg mL^{-1}, respectively) and inoculated with 1% ONC. Cells were grown at 37 °C, 120 rpm for 6 h. Cultures were induced with 5 µL IPTG (from 1 M stock, final conc.: 1 mM), supplemented with 5 µL 5-aminolevulinic acid (from 1 M stock, final conc.: 1 mM) and incubated at 13 or 20 °C [ArcticExpress(DE3) or BL21(DE3), respectively], 120 rpm for 22 h. The cultures were centrifuged (4000 rpm, 15 °C for 30 min) and the pellet was used for the biotransformations.

Table 1: Details on enzymes and their preparation

Name	pEG*	Source organism	Source of plasmid	*E. coli* host strain	Plasmid backbone	Tag (C or N terminus)	Medium	Antibiotic		Inducer		Additives	Preculture		Expression		
								Name	Conc. (µg mL^{-1})	Name	Conc. (mM)		T (°C)	rpm	T (°C)	rpm	time [h]
PsDRR	497	*Papaver somniferum*	[9]	ArcticExpress (DE3)	pET-47b	His6 (N)	LB	Kan	50	IPTG	1	None	30	120	20	120	20-24
Ntrunc-SalSyn	752	*Papaver somniferum*	This work based on[10]	BL21(DE3)	pET-28a	His6 (C)	TB	Kan	50	IPTG	1	5-Ala (1 mM)	30	120	30	120	6
Nswap-SalSyn	753	*Papaver somniferum*	This work	BL21(DE3)	pET-28a	His6 (C)	TB	Kan	50	IPTG	1	5-Ala (1 mM)	30	120	30	120	6
AtR2	754	*Arabidopsis thaliana*	This work	BL21(DE3)	pCDF-1b	S (C)	TB	Spec	100	IPTG	1	None	30	120	30	120	6

* Internal reference number. pEG stands for “plasmids of the Elk Group”. Please refer to this number in case of any inquiries.

3 Enzymatic reduction of dehydroreticuline **2** to (*R*)-reticuline (*R*)-**1**

Microcentrifuge vials (1.5 mL) were charged with a stock solution of D-glucose (1.25 M stock, final conc.: 9 mg mL^{-1} = 50 mM) and a stock of PsDRR-enzyme preparation (lyophilized cells or CFE resuspended in buffer). GDH solution (50 mg mL^{-1} stock, final conc.: 2 mg mL^{-1}), substrate solution (150 mM stock of **2**, final conc.: 10-100 mM.), $NADP^+$ solution (20 mM stock, final conc.: 0-2 mM), optionally cosolvent (DMSO, ethanol, isopropanol, acetone) and Tris-HCl buffer (0.1 M, pH 8.5) were added to reach a final volume of 500 µl. The reaction samples were mixed at 30 °C, 900 rpm for 20 h and quenched with 500 µl methanol. The samples were centrifuged, filtrated through cotton, and injected to HPLC-UV.

This procedure was slightly changed for different studies.

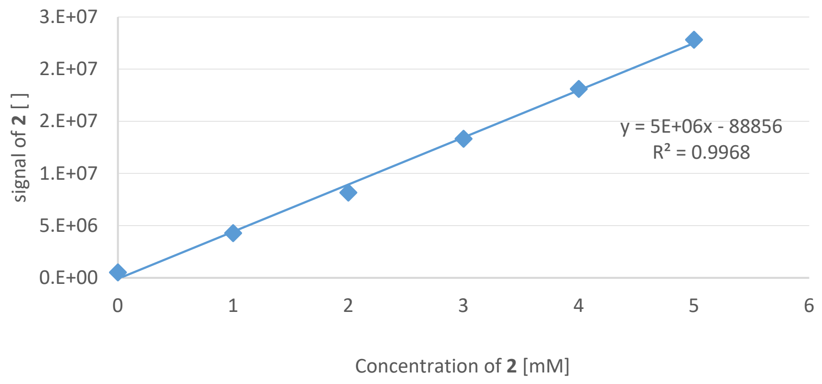

Figure S3: Calibration curve of the substrate **2** for the concentration range 0-5 mM (in duplicates).

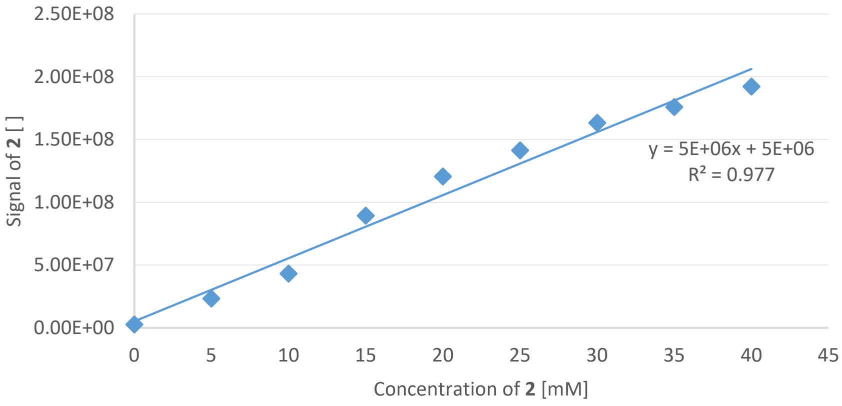

Figure S4: Calibration curve of the substrate **2** for the concentration range 0-40 mM (in duplicates).

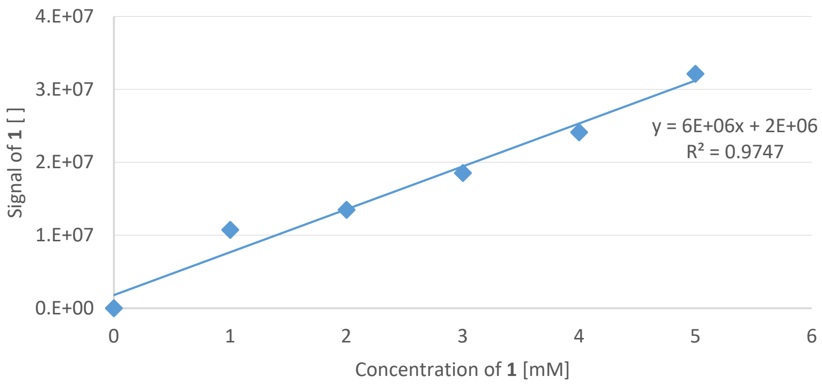

Figure S5: Calibration curve of the product *rac*-**1** for the concentration range 0-5 mM (in duplicates).

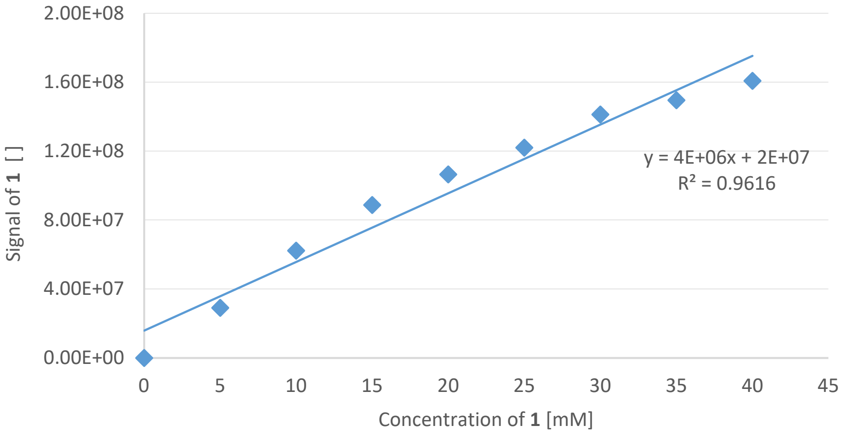

Figure S6: Calibration curve of the product *rac*-**1** for the concentration range 0-40 mM (in duplicates).

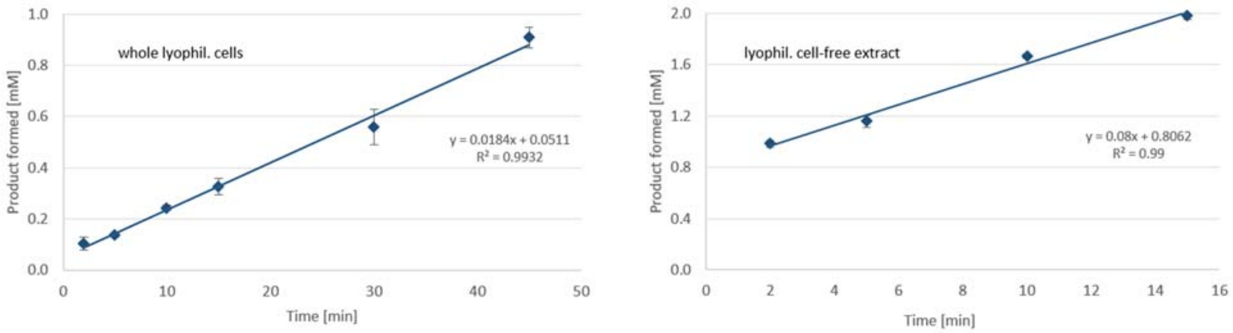

Figure S7: Activity assay systems for the reduction of **2** to (*R*)-**1** employing whole lyophilized cells or lyophilized cell-free extract containing PsDRR. Reaction conditions: substrate **2** (2 mM), PsDRR preparation (lyophilized cells (left) or CFE (right), 10 mg mL^{-1}), $NADP^+$ (1 mM), D-glucose (50 mM), GDH (24 U mL^{-1}), KPi buffer (100 mM, pH 7.5), 30 °C, 900 rpm, 0-45 min. The experiment was performed in triplicates.

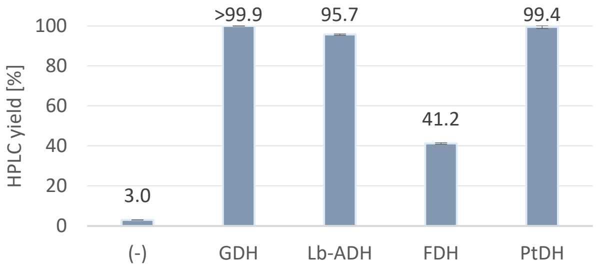

Figure S8: Investigation of different cofactor regeneration systems for the reduction of **2** to (*R*)-**1** employing whole lyophilized cells containing PsDRR. Reaction conditions: substrate **2** (30 mM), PsDRR preparation (lyophilized cells, 0.06 U mL^{-1}), NADP^{+} (1 mM), cofactor regeneration systems: D-glucose, 2-propanol, ammonium formate or sodium phosphite (50 mM), GDH, ADH, FDH or PtDH (2 mg mL^{-1}), KPi buffer (100 mM, pH 7.5), 30 °C, 900 rpm, 20 h. Control reaction (-): no cofactor regeneration enzyme and no co-substrate (e.g. D-glucose). The experiment was performed in triplicates.

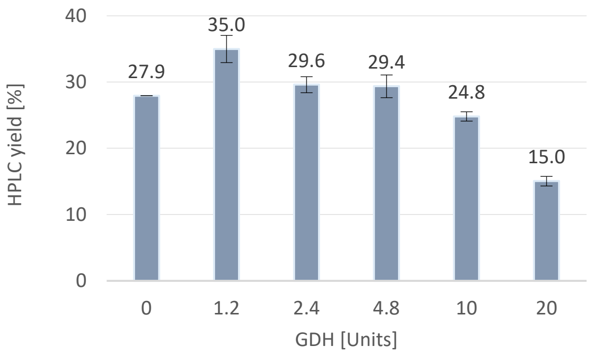

Figure S9: Investigation of different cofactor concentration for the reduction of **2** to (*R*)-**1** employing whole lyophilized cells containing PsDRR. Reaction conditions: substrate **2** (30 mM), PsDRR preparation (lyophilized cells, 0.06 U mL^{-1}), NADP^{+} (0-2 mM), D-glucose (50 mM), GDH (0-20 U mL^{-1}), KPi buffer (100 mM, pH 7.5), DMSO [10% (v/v)], 30 °C, 900 rpm, 20 h. The experiment was performed in triplicates.

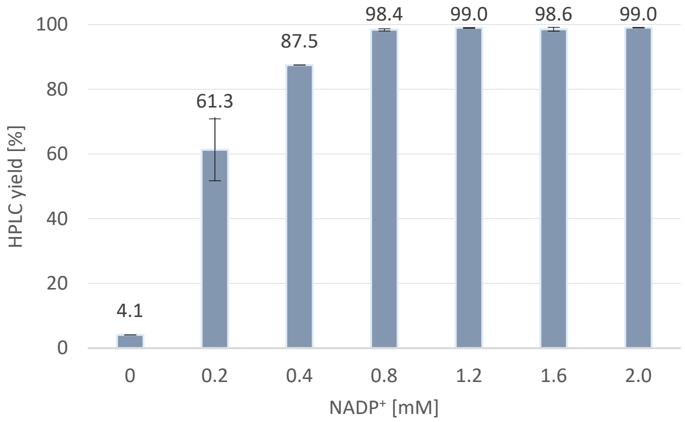

Figure S10: Investigation of the NADP^{+} concentration for the reduction of **2** to (*R*)-**1** employing whole lyophilized cells containing PsDRR. Reaction conditions: substrate **2** (30 mM), PsDRR preparation (lyophilized cells, 0.06 U mL^{-1}), NADP^{+} (0-2 mM), D-glucose (50 mM), GDH (4.5 U mL^{-1}), KPi buffer (100 mM, pH 7.5), DMSO [10% (v/v)], 30 °C, 900 rpm, 20 h. The experiment was performed in triplicates.

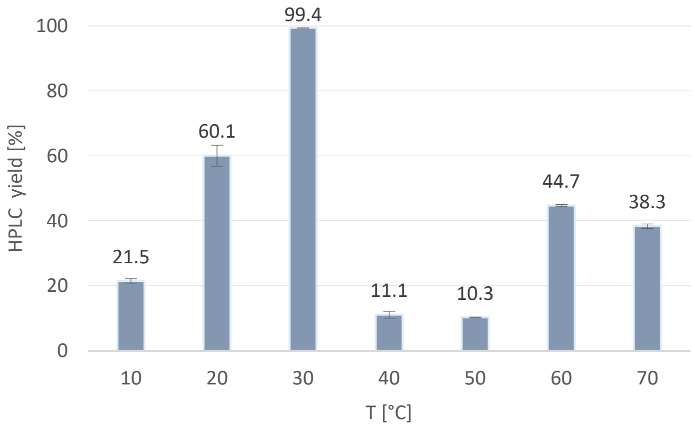

Figure S11: Investigation of different reaction temperatures for the reduction of **2** to (*R*)-**1** employing whole lyophilized cells containing PsDRR. Reaction conditions: substrate **2** (30 mM), PsDRR preparation (lyophilized cells, 0.06 U mL^{-1}), NADP$^+$ (1 mM), D-glucose (50 mM), GDH (4.5 U mL^{-1}), KPi buffer (100 mM, pH 7.5), 10-70 °C, 900 rpm, 20 h. The experiment was performed in triplicates.

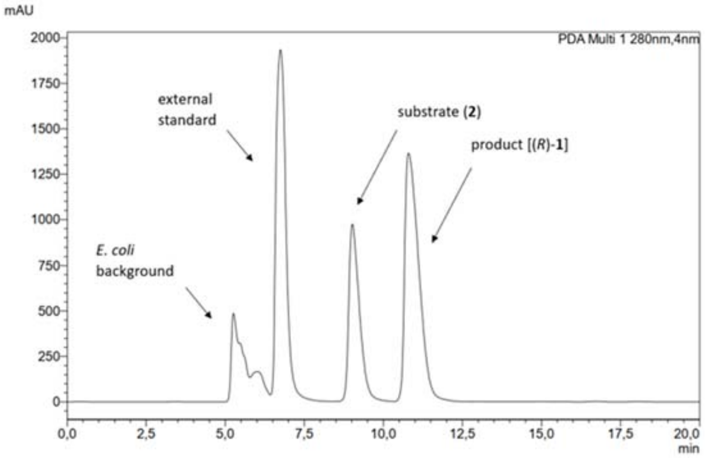

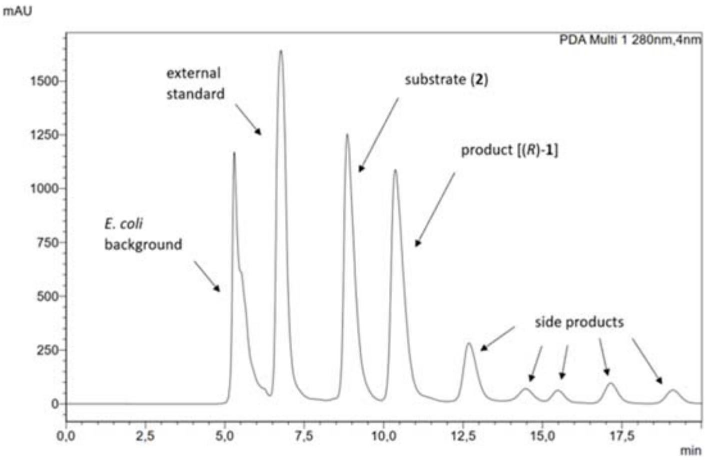

Figure S12: HPLC-DAD chromatograms of the temperature study shown in Figure S11. Above: biotransformation performed at 20 °C; Below: biotransformation performed at 60 °C.

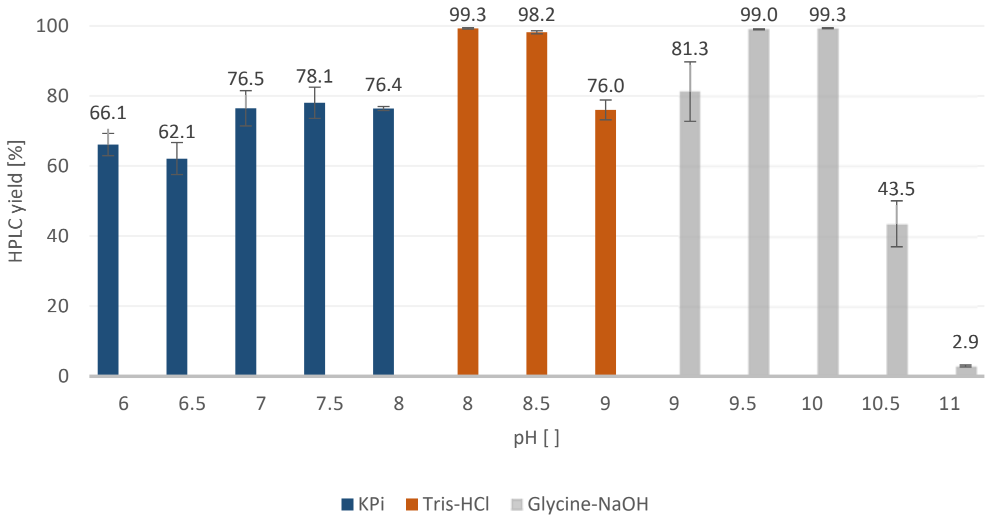

Figure S13: Investigation of the optimal pH and buffer for the reduction of **2** to (*R*)-**1**. Reaction conditions: substrate **2** (30 mM), *Ps*DRR preparation (lyophilized cells, 0.06 U mL^{-1}), NADP$^+$ (1 mM), D-glucose (50 mM), GDH (4.5 U mL^{-1}), buffer (KPi, Tris-HCl, glycine-NaOH, 100 mM, pH 6-11), 30 °C, 900 rpm, 20 h. The experiment was performed in triplicates.

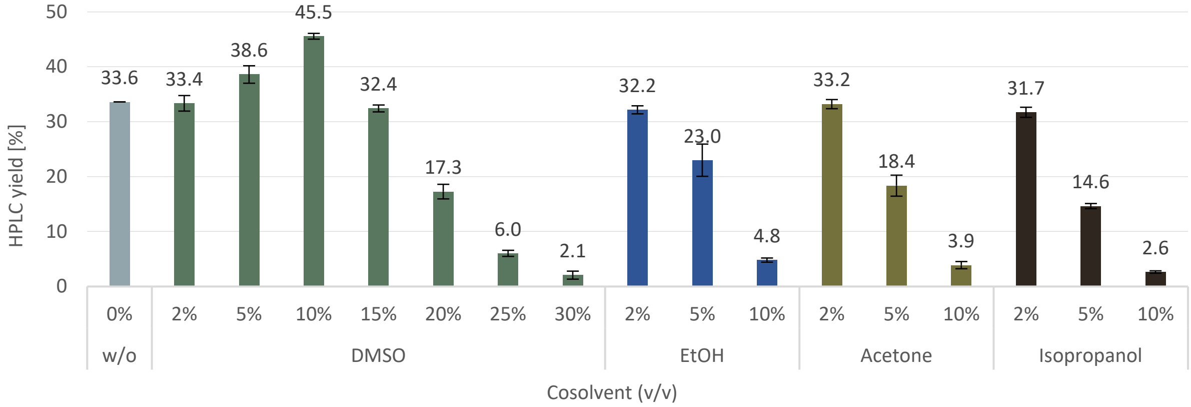

Figure S14: Co-solvent optimization study for the reduction of **2** to (*R*)-**1** employing whole lyophilized cells containing *Ps*DRR. Reaction conditions: substrate **2** (30 mM), *Ps*DRR preparation (lyophilized cells, 0.06 U mL^{-1}), NADP$^+$ (1 mM), D-glucose (50 mM), GDH (4.5 U mL^{-1}), KPi buffer (100 mM, pH 7.5), cosolvent [0-30% (v/v) DMSO, EtOH, acetone or 2-PrOH), 30 °C, 900 rpm, 20 h. The experiment was performed in triplicates.

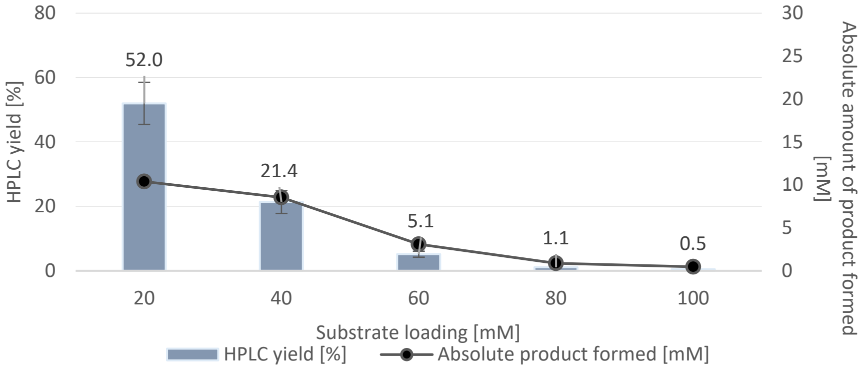

Figure S15: Substrate loading study of the reduction of **2** to (*R*)-**1** using optimized conditions. Reaction conditions: substrate **2** (20-100 mM), PsDRR preparation (lyophilized cells, 0.06 U mL^{-1}), $NADP^+$ (1 mM), D-glucose (50 mM), GDH (4.5 U mL^{-1}), Tris-HCl buffer (100 mM, pH 8.5), DMSO [10% (v/v)], 30 °C, 900 rpm, 20 h. The experiment was performed in triplicates.

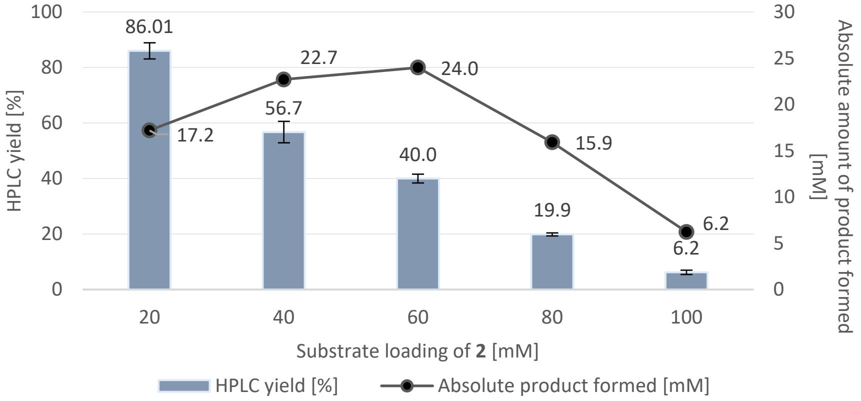

Figure S16: Substrate loading study of the reduction of **2** to (*R*)-**1** using not optimized conditions. Reaction conditions: substrate **2** (20-100 mM), PsDRR preparation (lyophilized cell-free extract, 0.16 U mL^{-1}), $NADP^+$ (1 mM), D-glucose (50 mM), GDH (4.5 U mL^{-1}), KPi buffer (100 mM, pH 7.0), 30 °C, 900 rpm, 20 h. The experiment was performed in triplicates.

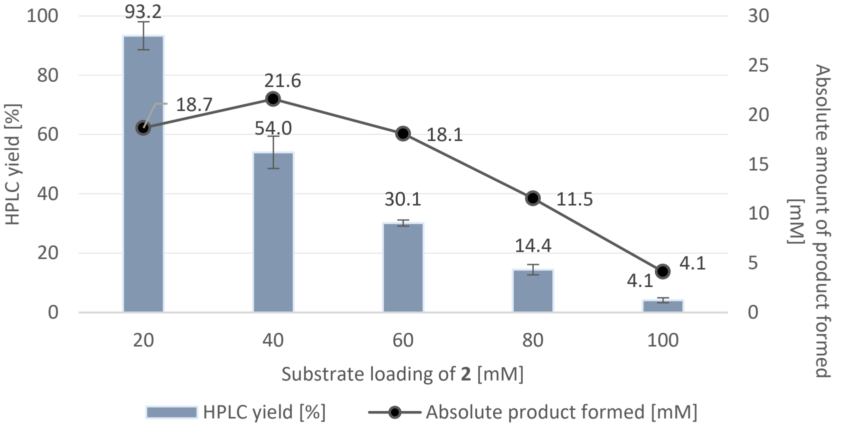

Figure S17: Substrate loading study of the reduction of **2** to (*R*)-**1** using optimized conditions. Reaction conditions: substrate **2** (20-100 mM), PsDRR preparation (lyophilized cell-free extract, 0.16 U mL^{-1}), $NADP^+$ (1 mM), D-glucose (50 mM), GDH (4.5 U mL^{-1}), Tris-HCl buffer (100 mM, pH 8.5), DMSO [10% (v/v)], 30 °C, 900 rpm, 20 h. The experiment was performed in triplicates.

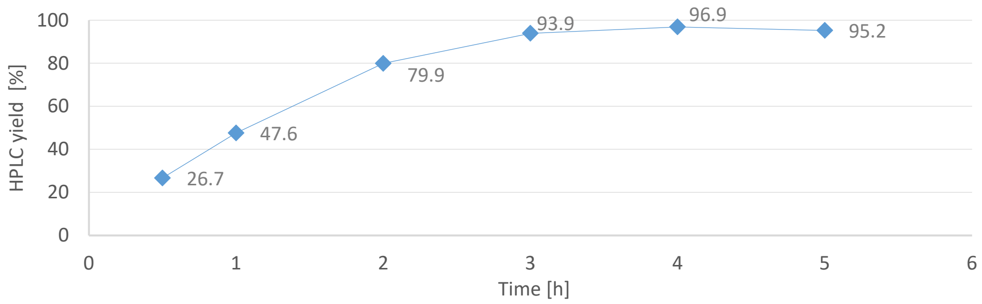

Figure S18: Reaction time study of the reduction of **2** to (*R*)-**1**. Reaction conditions: substrate **2** (10 mM), *Ps*DRR preparation (lyophilized cell-free extract, 0.16 U mL^{-1}), $NADP^+$ (1 mM), D-glucose (50 mM), GDH (4.5 U mL^{-1}), KPi buffer (100 mM, pH 7.5), 30 °C, 900 rpm.

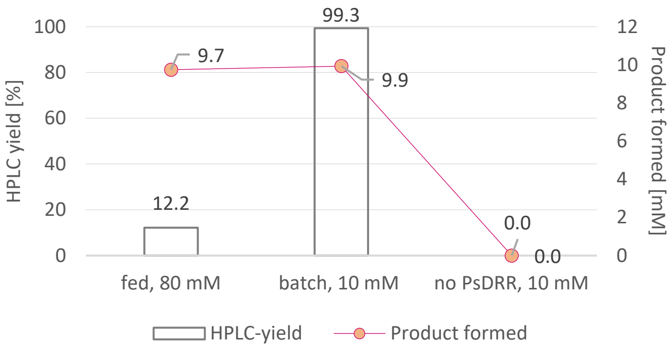

Figure S19: Fed batch system for the reduction of **2** to (*R*)-**1**. Reaction conditions: substrate **2** (80 mM: addition of 10 mM each 4 h), *Ps*DRR preparation (lyophilized cells, 0.06 U mL^{-1}), $NADP^+$ (1 mM), D-glucose (50 mM), GDH (4.5 U mL^{-1}), KPi buffer (100 mM, pH 7.5), DMSO [10% (v/v)], 30 °C, 900 rpm, 32 h. The experiment was performed in duplicates.

(*R*)-1-(3-hydroxy-4-methoxybenzyl)-6-methoxy-2-methyl-1,2,3,4-tetrahydroisoquinolin-7-ol [(*R*)-**1**]

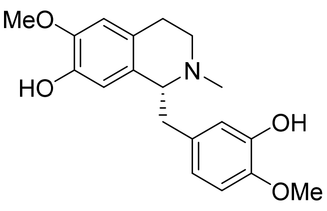

A 250 mL baffled Erlenmeyer flask was charged with lyophilized cells harboring *Ps*DRR (1.7 g, final conc.: 0.06 U mL^{-1}) and resuspended in 50 mL Tris-HCl buffer (0.1 M, pH 8.5). The iminium salt was added as a solid (200 mg, 0.54 mmol) whereas D-glucose was added as a 0.9 M solution in buffer (3.0 mL, final conc.: 50 mM). The reaction was started by the addition of 1 mL of a 54 mM $NADP^+$ solution in buffer (final conc.: 1 mM). The cultivation flask was capped with a metal cap and shaken at 30 °C, 140 rpm. After complete consumption of the substrate (24 , the reaction mixture was transferred to a 250 mL round bottom flask, flash-frozen and lyophilized overnight. The dried material was applied onto a silica-plug and eluted with EtOAc and MeOH. The filtrate containing the target product was concentrated under reduced pressure and the thus received yellow oil (~1.5 g) was purified by column chromatography (size: 29 x 1.9 cm, CH_2Cl_2/MeOH/NH_4OH = 15:1:1% to 7:1:1%) and dried under vacuum.

Yield: 162 mg (492 µmol, 92%) pale yellow crystals.

R_f = 0.37 (CH_2Cl_2/MeOH/NH_4OH = 9:1:1%) (254 nm, CAM: dark blue).

^{1}H NMR (300.13 MHz, $CDCl_3$) δ = 6.77-6.71 (dd, J = 11.3, 5.0 Hz, 2H, CH_{arom}), 6.65 – 6.48 (m, 2H, CH_{arom}), 6.36 (s, 1H, CH_{arom}), 4.69 (brs, 2H, ArOH), 3.84 (d, J = 2.6 Hz, 6H, OCH_3), 3.74 – 3.61 (m, 1H, CH), 3.25 – 3.10 (m, 1H, CH_2), 3.03 (dd, J = 14.1, 6.1 Hz, 1H, CH_2), 2.88 – 2.68 (m, 3H, CH_2), 2.65 – 2.50 (m, 1H, CH_2), 2.46 (s, 3H, NCH_3).

^{13}C NMR (75.47 MHz, $CDCl_3$) δ = 145.46 (C_q), 145.29 (C_q), 145.16 (C_q), 143.49 (C_q), 133.24 (C_q), 130.25 (C_q), 125.27 (C_q), 121.02 (CH_{arom}), 115.76 (CH_{arom}), 113.86 (CH_{arom}), 110.71 (CH_{arom}), 110.58 (CH_{arom}), 64.60 (CH), 56.03 (OCH_3), 55.95 (OCH_3), 46.80 (CH_2), 42.48 (NCH_3), 41.06 (CH_2), 25.06 (CH_2).

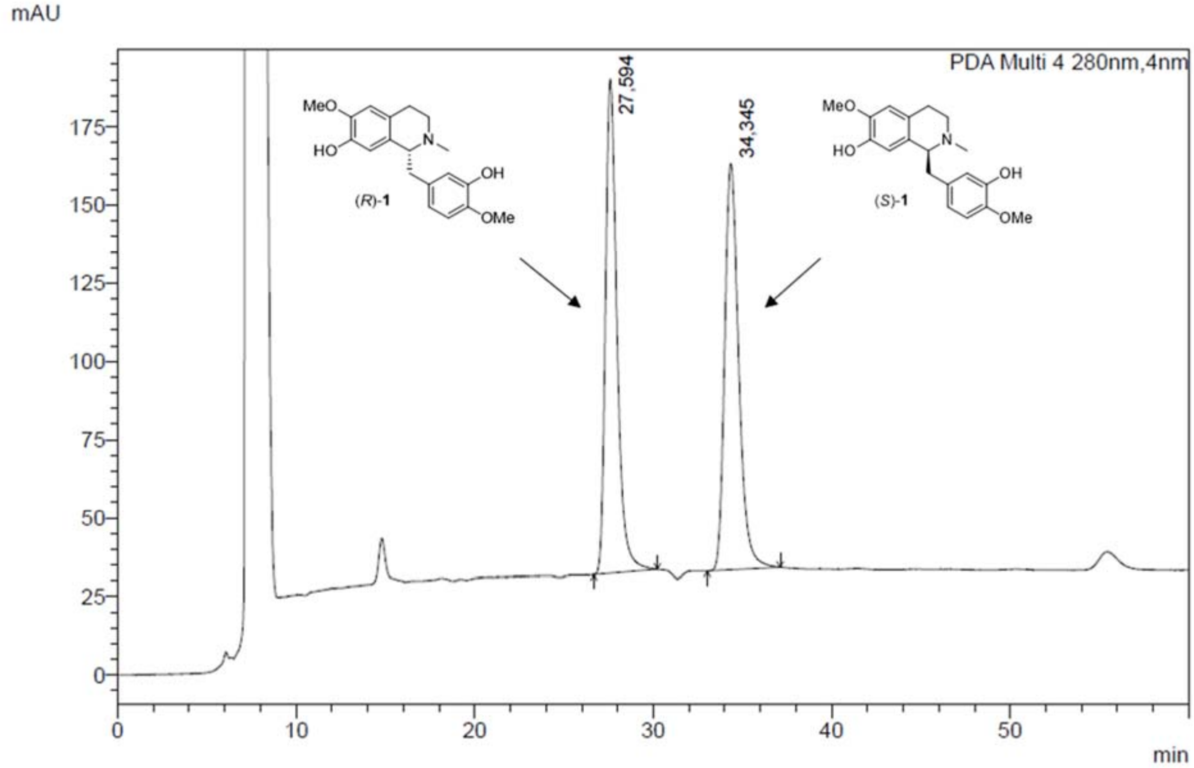

Figure S20: Normal-phase chromatogram of the separation of (*R*)- and (*S*)-**1**.

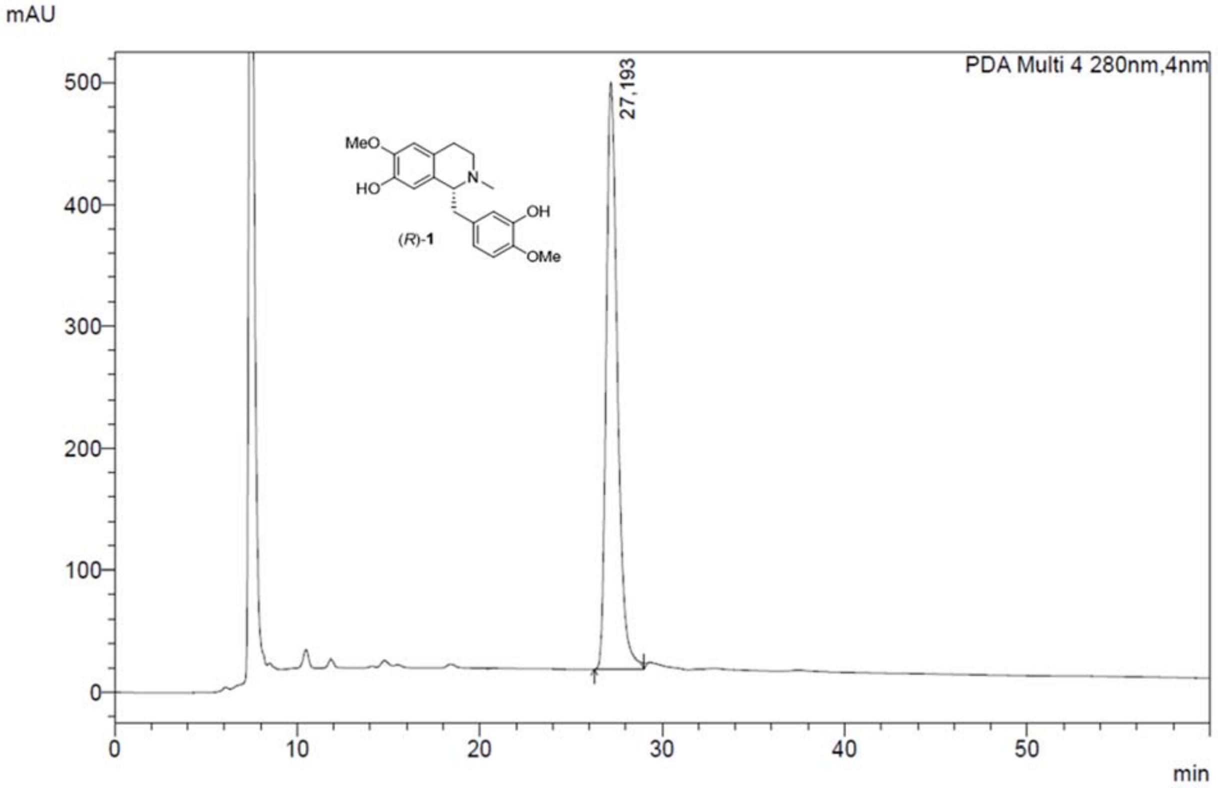

Figure S21: Normal-phase chromatogram of (*R*)-**1** from the PsDRR-catalyzed biotransformation at 0.5 mmol scale using whole lyophilized *E. coli* cells.

4 Enzymatic C-C phenol coupling of (*R*)-reticuline (*R*)-**1** to (+)-salutaridine (+)-**3**

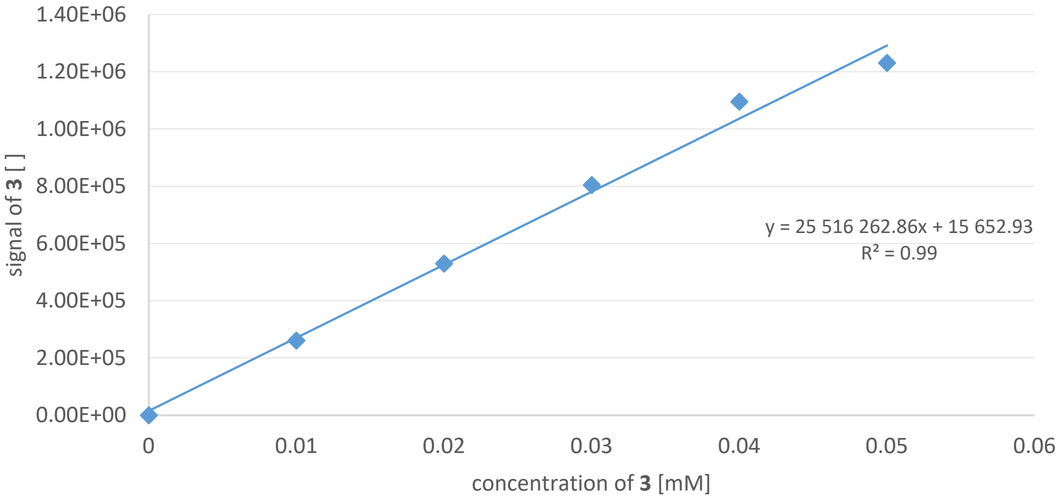

Figure S22: Calibration curve of the product salutaridine (**3**) for the concentration range 0-0.05 mM (in duplicates).

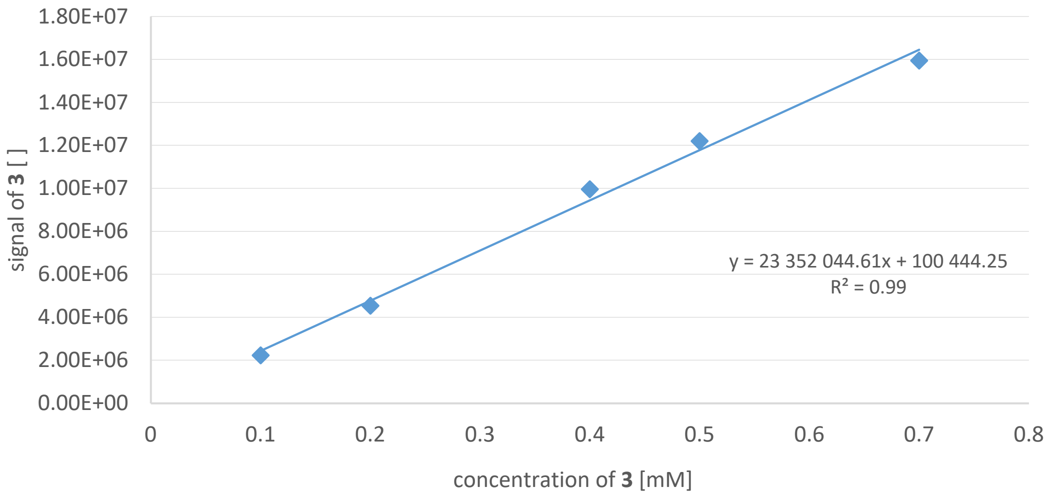

Figure S23: Calibration curve of the product salutaridine **3** for the concentration range 0.1-0.7 mM (in duplicates).

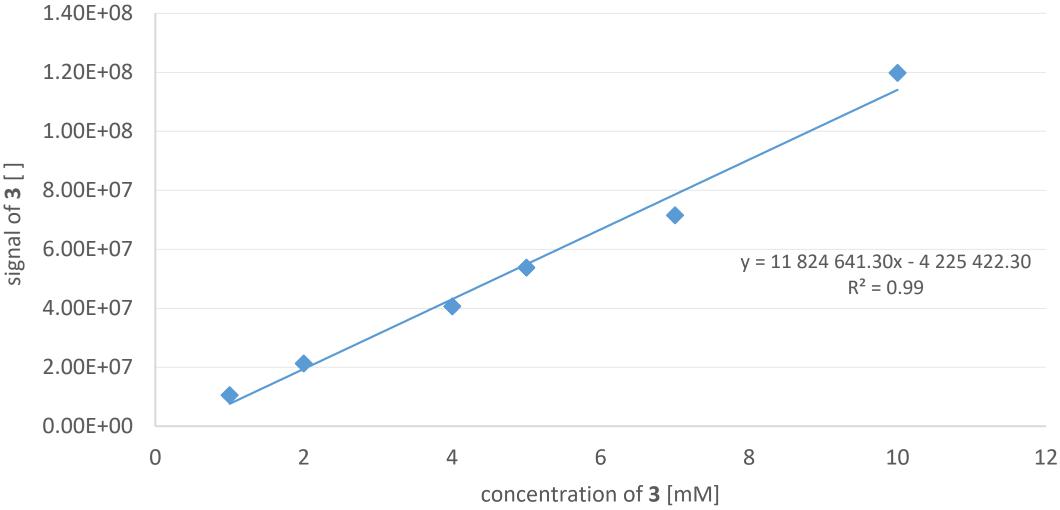

Figure S24: Calibration curve of the product salutaridine **3** for the concentration range 1-10 mM (in duplicates).

<u>Biotransformations employing SalSyn (CYP-CPR) strains: small-scale conversion studies</u>

The pellets obtained from the cultivation of the respective CYP-CPR strains in 10 mL DWPs were resuspended in KPi buffer (0.5 mL, 0.1 M, pH 7.5) and transferred to 2 mL DWPs. The substrate (*rac*-**2**) was added (1 µL, 100 mM stock in DMSO, final conc.: 0.2 mM) as well as NADPH solution (1 µL, 25 mM stock, final conc.: 0.05 mM) and D-glucose solution (20 µL, 1.25 M stock, final conc.: 50 mM).The biotransformations were incubated at 30 °C, 180 rpm for 20 h. The samples were quenched with methanol (200 µL) and transferred to fresh microcentrifuge vials. The samples were centrifuged (14680 rpm, 4 °C for 10 min) and the supernatant was filtrated through cotton. The samples were analyzed via HPLC-MS and –UV and TLC.

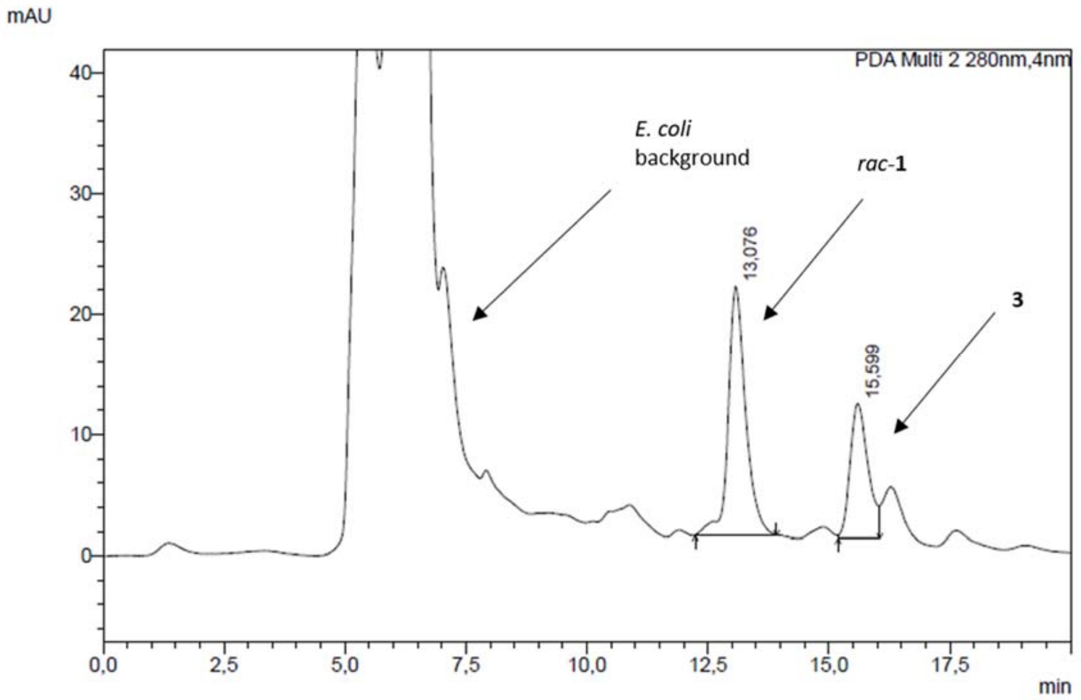

Figure S25: A selected example of a RP-HPLC measurement from conversion studies of **1** to **3** using whole *E. coli* cells harboring SalSyn and CPR. Eluent: buffer (30 mM $HCOONH_4$, pH 2.8)/methanol/acetonitrile = 67/30/3 (isocratic).

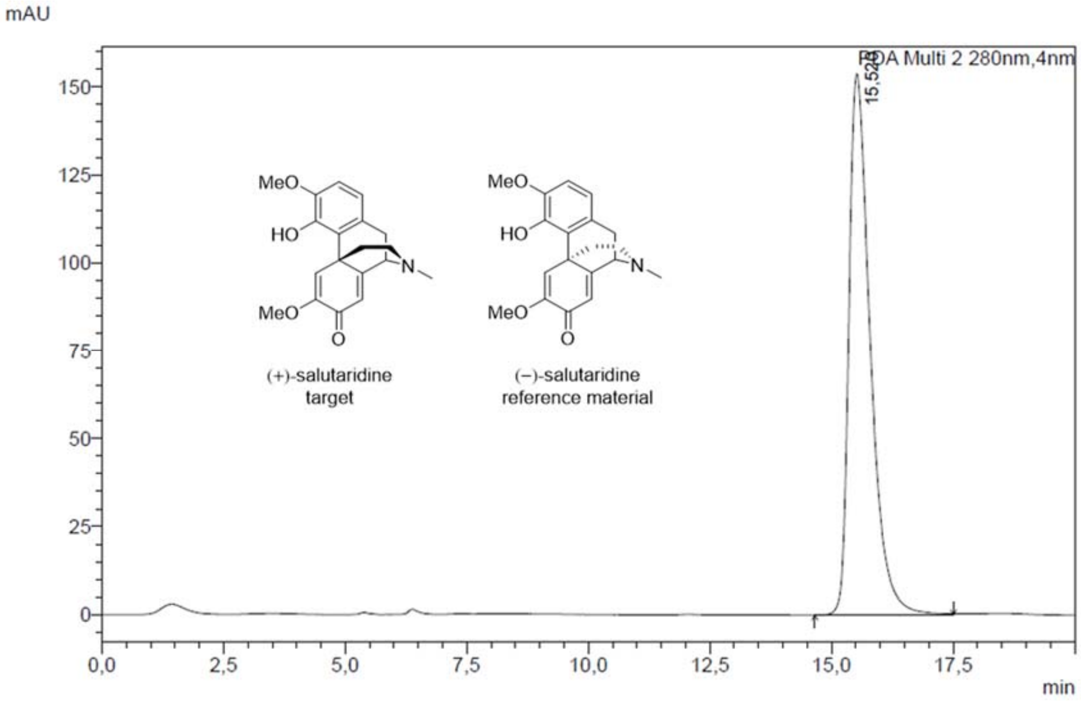

Figure S26: Achiral RP-HPLC measurement of the mirror image reference of the target product salutaridine (**3**). Eluent: buffer (30 mM $HCOONH_4$, pH 2.8)/methanol/acetonitrile = 67/30/3 (isocratic). CAS: 4090-18-0.

Equation 1: Calculation of the HPLC yield of salutaridine **3**. The factor 2 derives from the fact that *rac*-reticuline *rac*-**1** was used as substrate source, i.e., a maximum of 50% conversion to salutaridine was possible; f = response factor. The factor 2 (bold) was involved since *rac*-**1** was used rather than optically pure (*R*)-**1**, which is the only form accepted by the studied enzyme.

$$HPLC\ yield\ (salutaridine)[\%] = \frac{\mathbf{2} * peak\ area\ (salutaridine) * 100}{peak\ area\ (salutaridine) + f * (peak\ area\ (reticuline)}$$

$$f = \frac{\varepsilon_{280}(reticuline)}{\varepsilon_{280}\ (salutaridine)} = \frac{calibration\ slope\ (reticuline)}{calibration\ slope\ (salutaridine)}$$

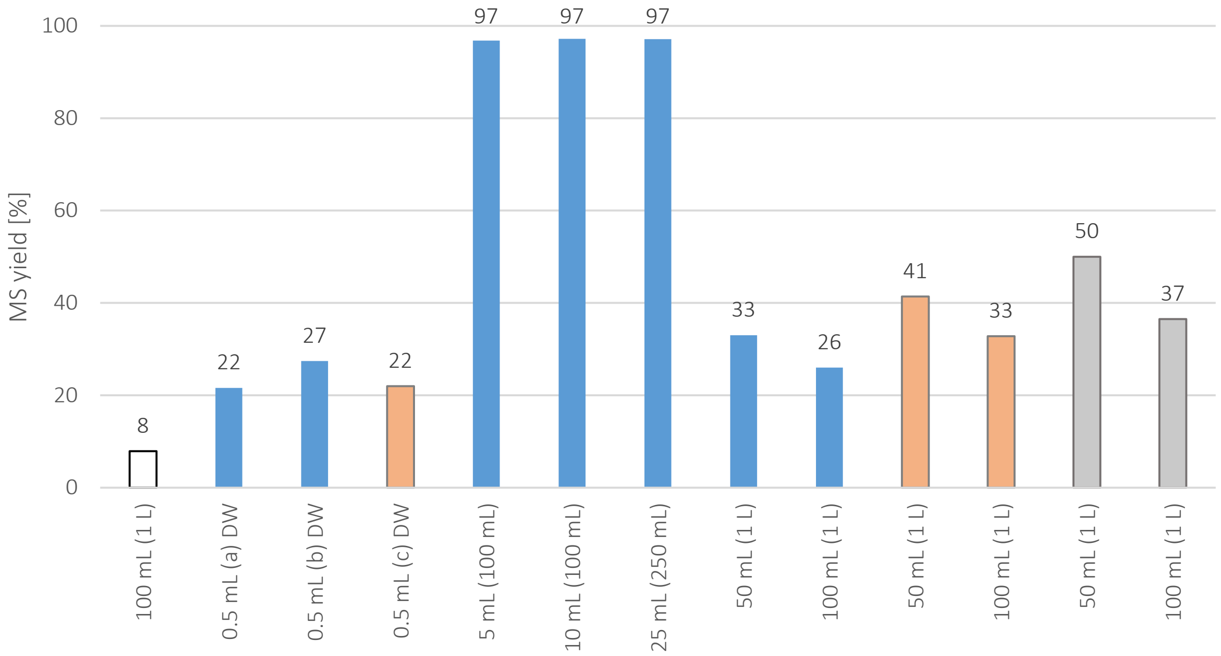

Figure S27: Studies on different volume to surface ratios for the biotransformation of (*R*)-1 towards (+)-**3**.

Semi-preparative scale

4-Hydroxy-3,6-dimethoxy-17-methyl-5,6,8,14-tetradehydromorphinan-7-one [(+)-**3**]

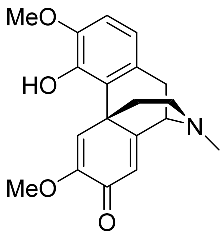

Three 2 L baffled Erlenmeyer flasks filled with autoclaved 660 mL TB medium were supplemented with kanamycin and spectinomycin solution (660 µL each, final conc.: 50 and 100 µg mL) and ONC (final conc.: 1%) of a cotransformed *E. coli* BL21(DE3) strain (harboring Nswap-KK-SalSyn and AtR2). The cells were grown at 20 °C, 120 rpm until OD_{600} = 1.0 was reached and the cultures were induced with IPTG (660 µL each, final conc.: 1 mM) and supplemented with a 5-ALA solution (660 µL each, final conc.:1 mM).

The enzymes were expressed at 20 °C, 120 rpm for 22 h and the combined culture was centrifuged (5000 rpm, 4 °C, 20 min). The supernatant was discarded, and the pellet was resuspended in KPi buffer (200 mL, 0.1 M, pH 7.5). The cell suspension was distributed to eight 250 mL baffled Erlenmeyer flasks (25 mL per flask) which were supplemented with D-glucose solution (in total 6.7 mL, final conc.: 50 mM), NADPH solution (in total 4.0 mL, final conc.: 0.1 mM) and a substrate stock of (*R*)-**1** (in total 12.8 mg per 1.5 mL DMSO, final conc.: 0.19 mM). The samples were incubated at 30 °C, 170 rpm for 24 h. Subsequently, the samples were pooled and the reaction mixture was centrifuged (5000 rpm, 4 °C, 20 min). The supernatant was transferred to a separatory funnel and the mixture was extracted with EtOAc (3x250 mL). The combined organic phase was concentrated under vacuum and the product was purified by column chromatography (size: 17 x 1.5 cm, CH_2Cl_2/MeOH/NH_4OH = 6:1:1%). The ^{1}H-NMR was recorded (Figure S50) and fits to literature.[11]

Yield: 3.5 mg (11 µmol, 27%) yellow viscous oil.

^{1}H NMR (700 MHz, CD_3OD) δ = 7.86 (s, 1H), 6.85 (d, *J* = 8.3 Hz, 1H), 6.67 (d, *J* = 8.3 Hz, 1H), 6.34 (s, 1H), 3.86 (s, 3H), 3.79 (d, *J* = 5.4 Hz, 1H), 3.74 (s, 3H), 3.41 – 3.34 (m, 1H), 3.05 – 2.97 (m, 1H), 2.63 (dd, J = 12.1, 3.6 Hz, 1H), 2.51 – 2.40 (m, 5H), 1.76 – 1.67 (m, 1H).

^{13}C-NMR (175.11 MHz, CD_3OD) δ = 183.35, 164.90, 151.81, 148.02, 145.56, 130.19, 125.10, 123.68, 123.01, 119.57, 111.56, 62.21, 56.72, 55.30, 47.98, 45.27, 41.65, 38.21, 33.60.

5 Experimental spectra

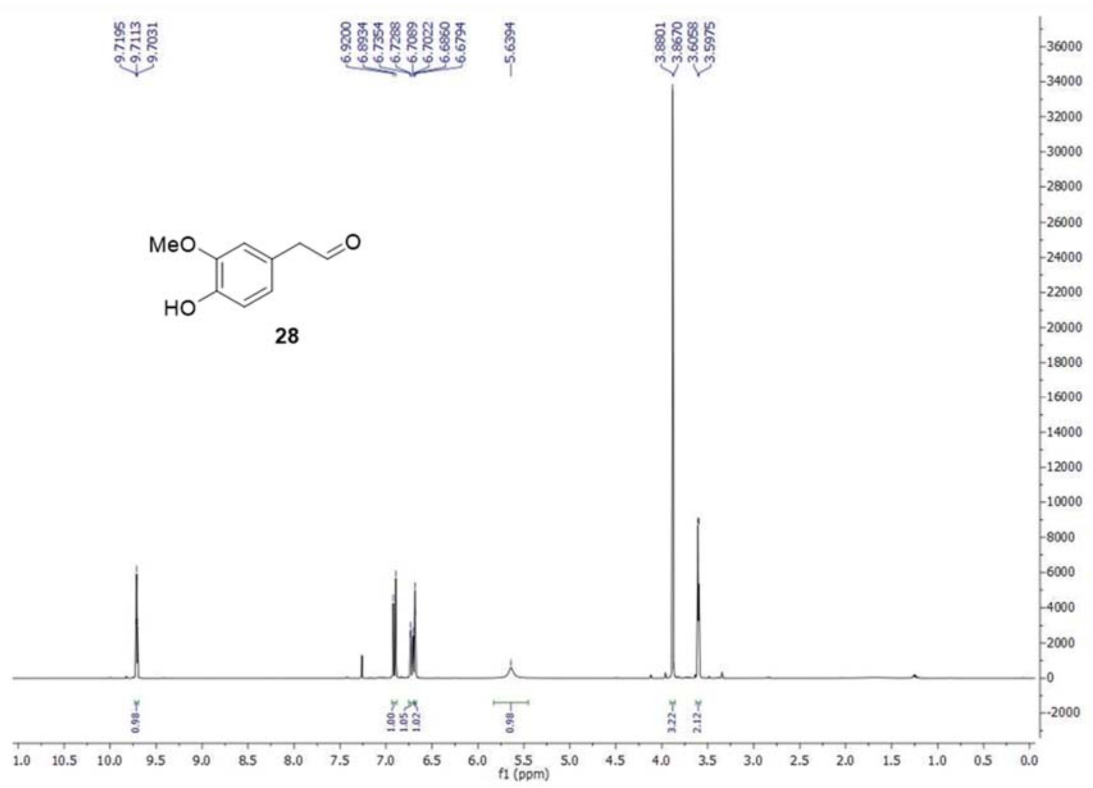

Figure S28: ^{1}H-NMR (300.13 MHz, $CDCl_3$) of 2-(4-hydroxy-3-methoxyphenyl)acetaldehyde (**28**).

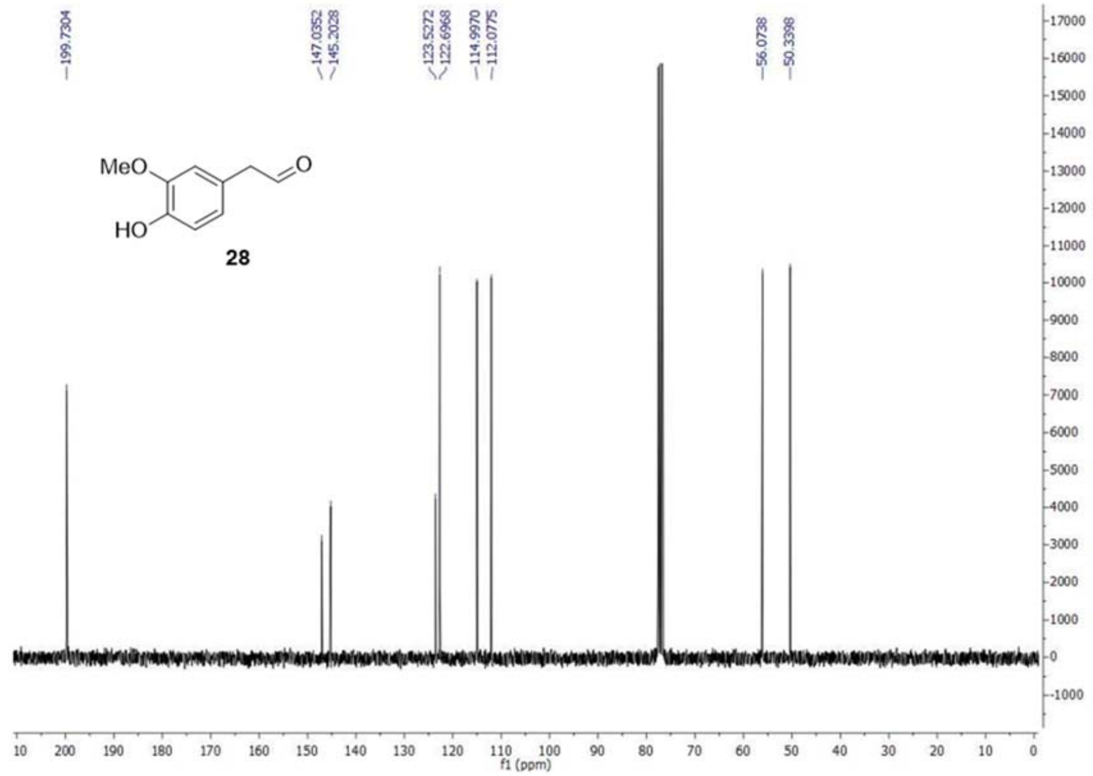

Figure S29: ^{13}C-NMR (75.47 MHz, $CDCl_3$) of 2-(4-hydroxy-3-methoxyphenyl)acetaldehyde (**28**).

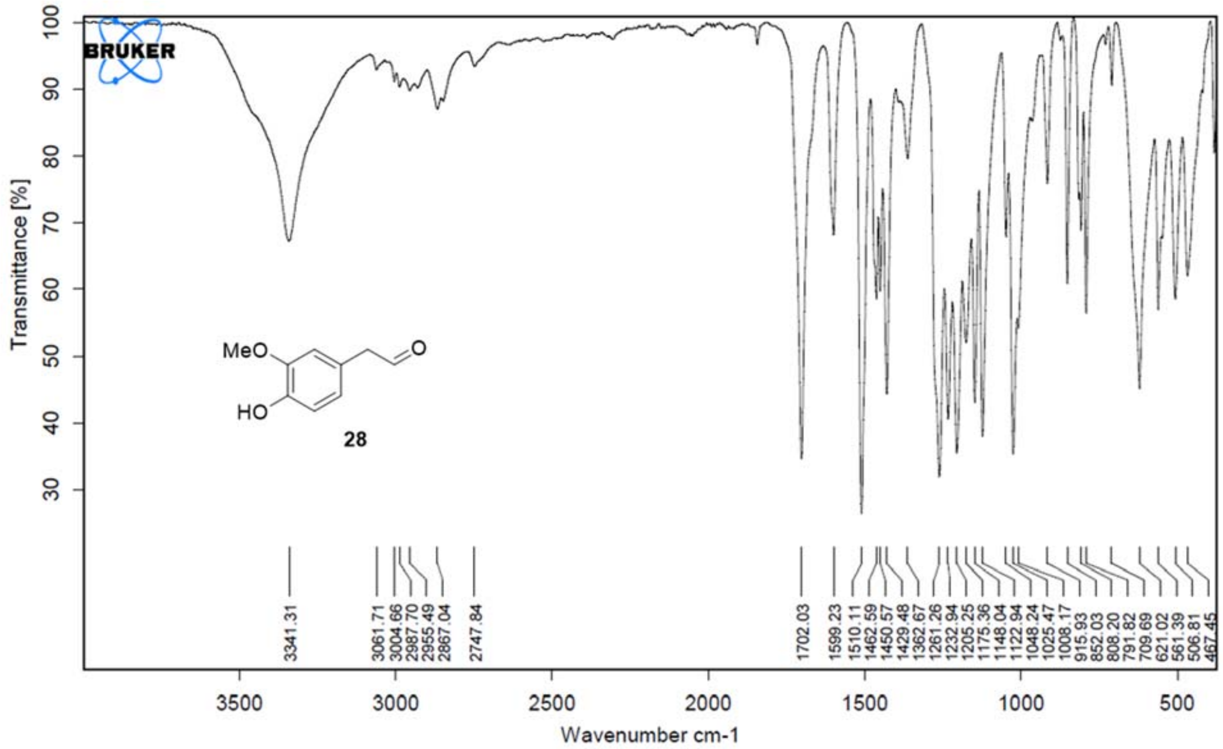

Figure S30: ATR-IR spectrum of 2-(4-hydroxy-3-methoxyphenyl)acetaldehyde (**28**).

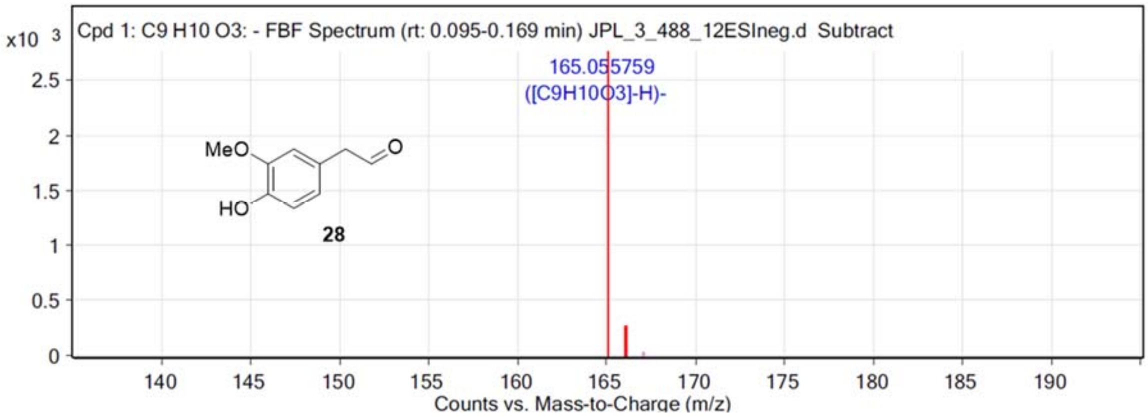

Figure S31: HR-MS spectrum of 2-(4-hydroxy-3-methoxyphenyl)acetaldehyde (**28**).

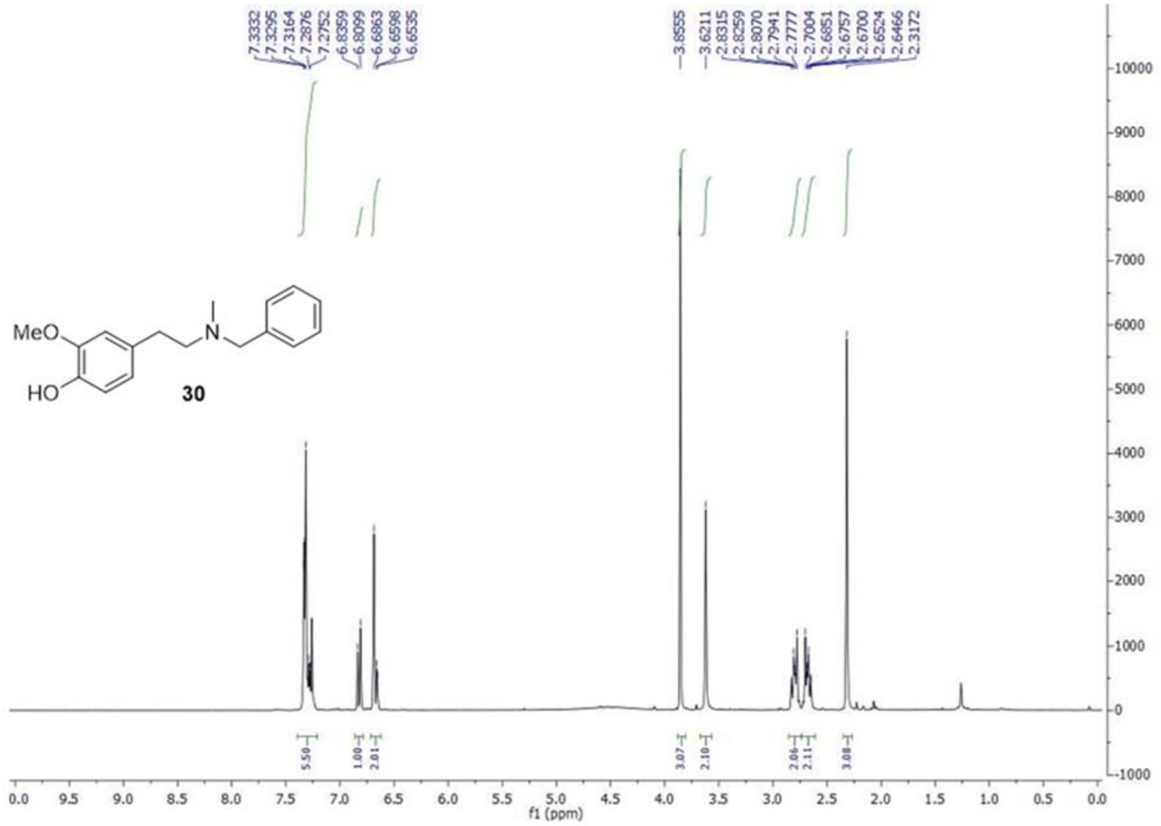

Figure S32: ^{1}H-NMR (300.13 MHz, $CDCl_3$) of 4-(2-(benzyl(methyl)amino)ethyl)-2-methoxyphenol (**30**).

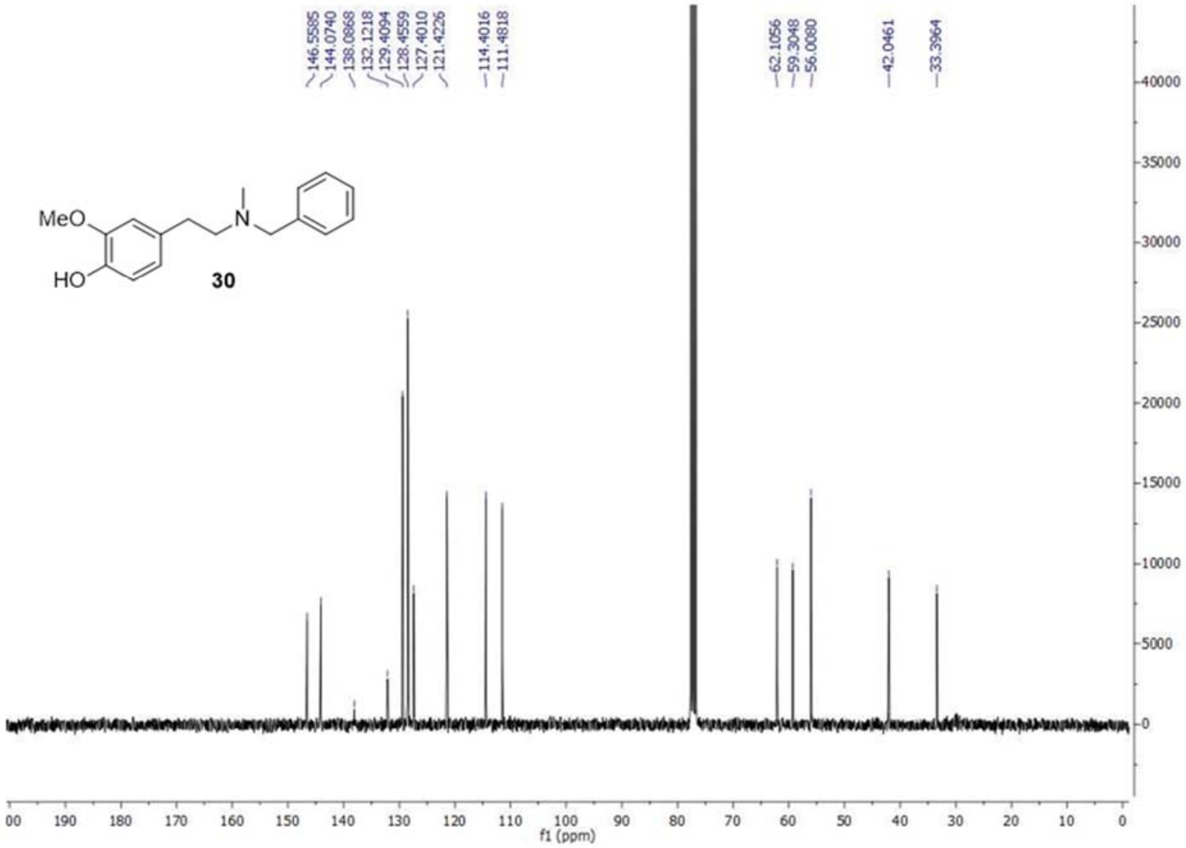

Figure S33: ^{13}C-NMR (75.47 MHz, $CDCl_3$) of 4-(2-(benzyl(methyl)amino)ethyl)-2-methoxyphenol (**30**).

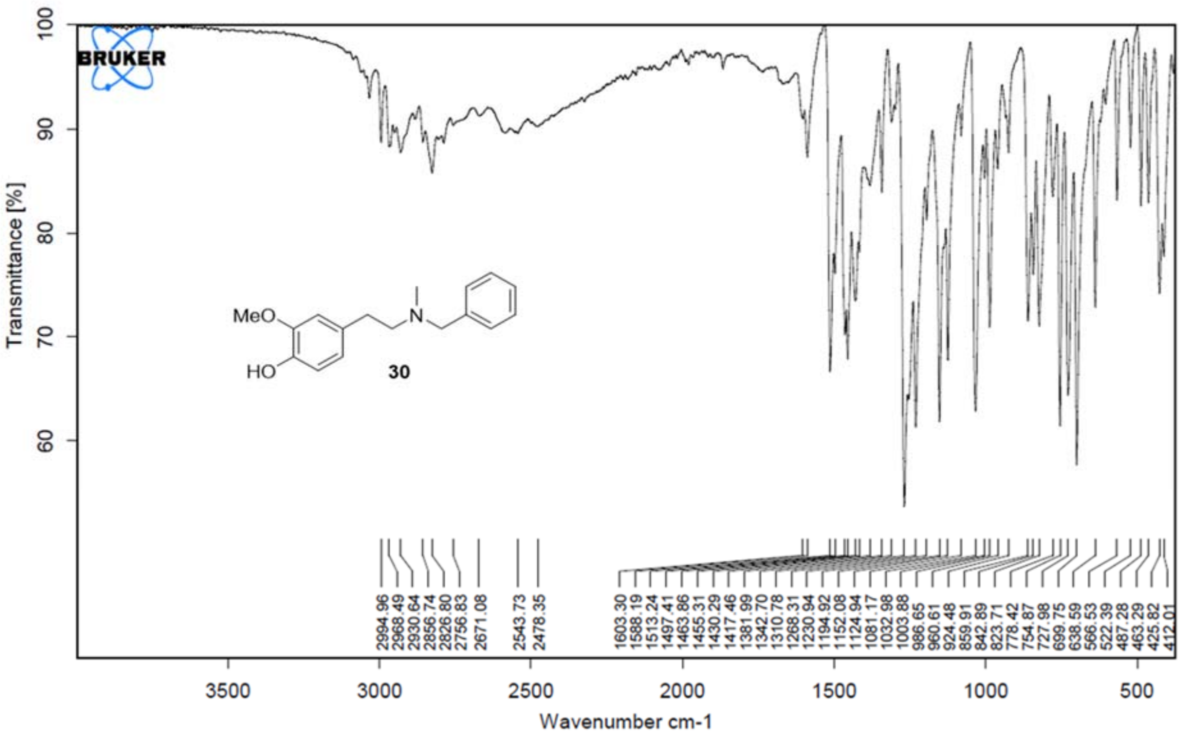

Figure S34: ATR-IR spectrum of 4-(2-(benzyl(methyl)amino)ethyl)-2-methoxyphenol (**30**).

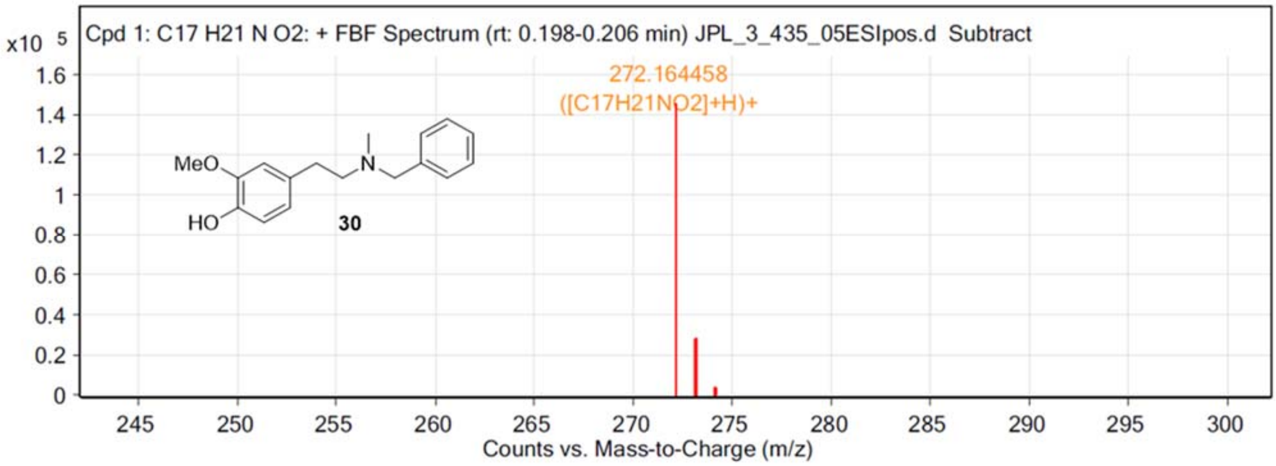

Figure S35: HR-MS spectrum of 4-(2-(benzyl(methyl)amino)ethyl)-2-methoxyphenol (**30**).

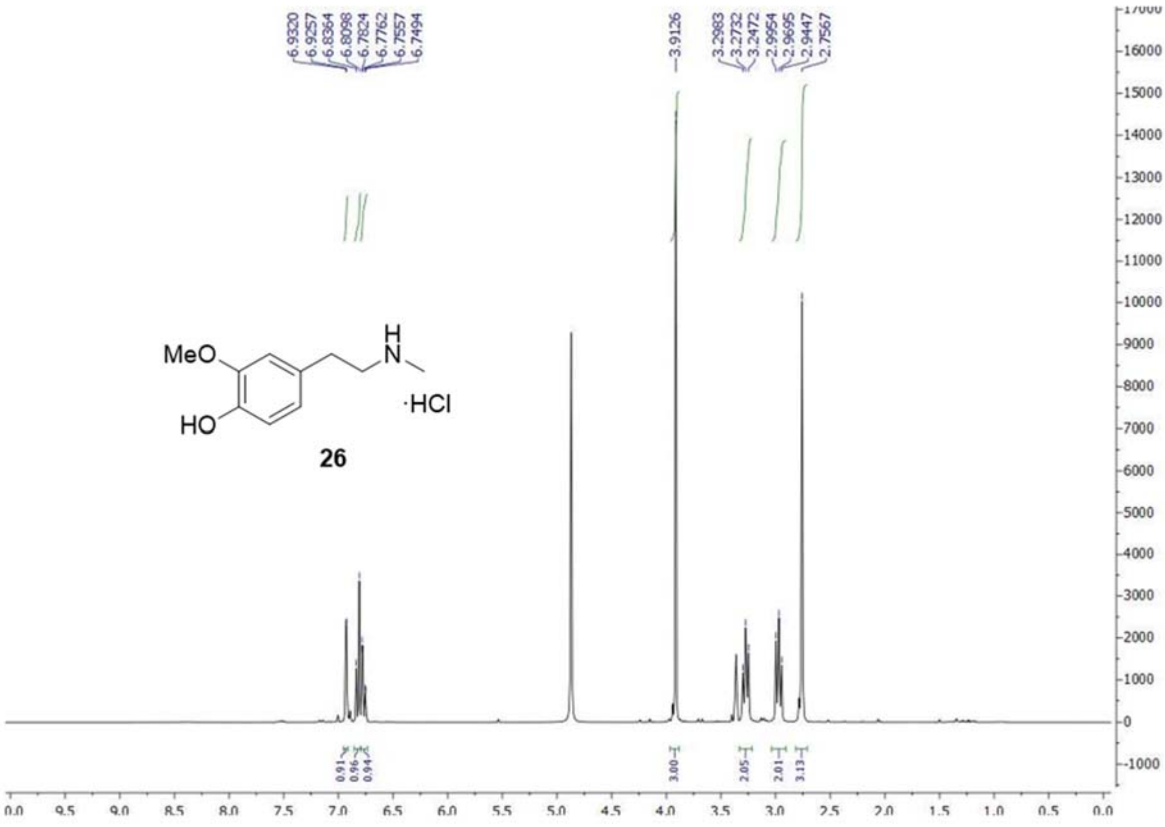

Figure S36: ^{1}H-NMR (300.13 MHz, MeOD-d$_4$) of 2-methoxy-4-(2-(methylamino)ethyl)phenol hydrochloride (**26**).

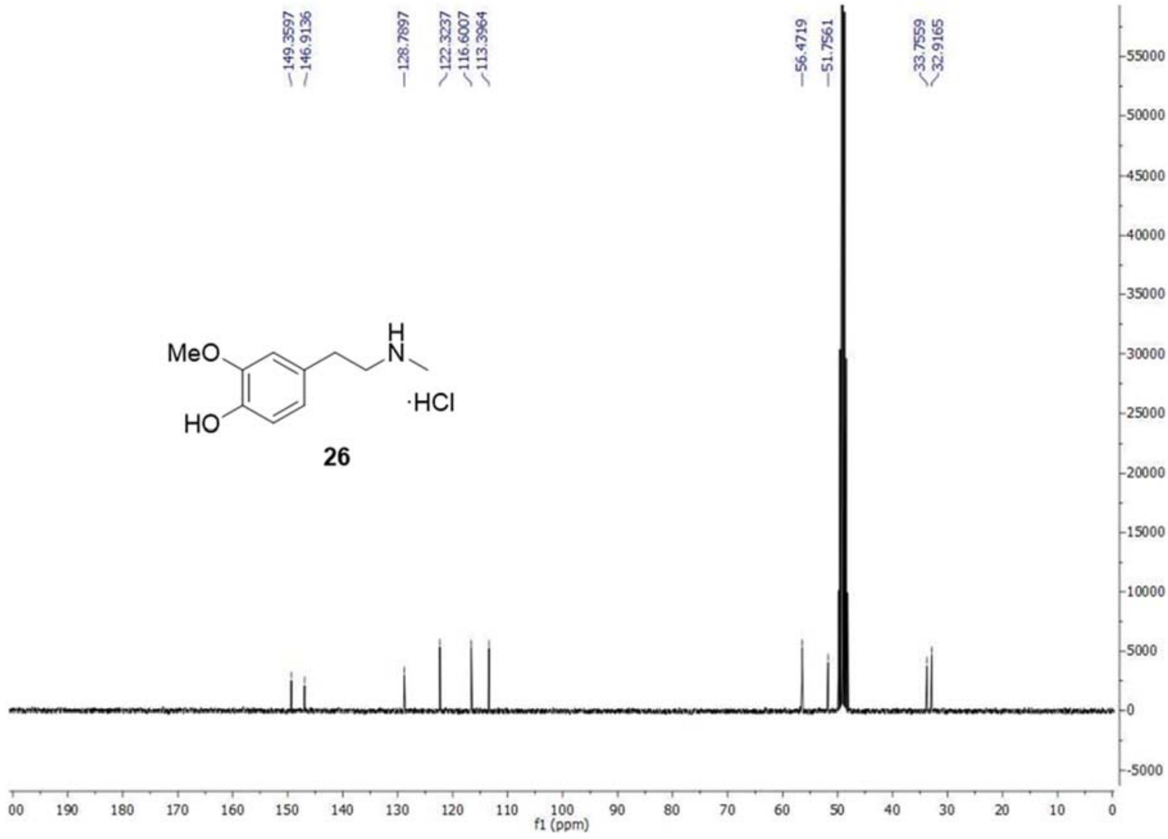

Figure S37: ^{13}C-NMR (75.47 MHz, MeOD-d$_4$) of 2-methoxy-4-(2-(methylamino)ethyl)phenol hydrochloride (**26**).

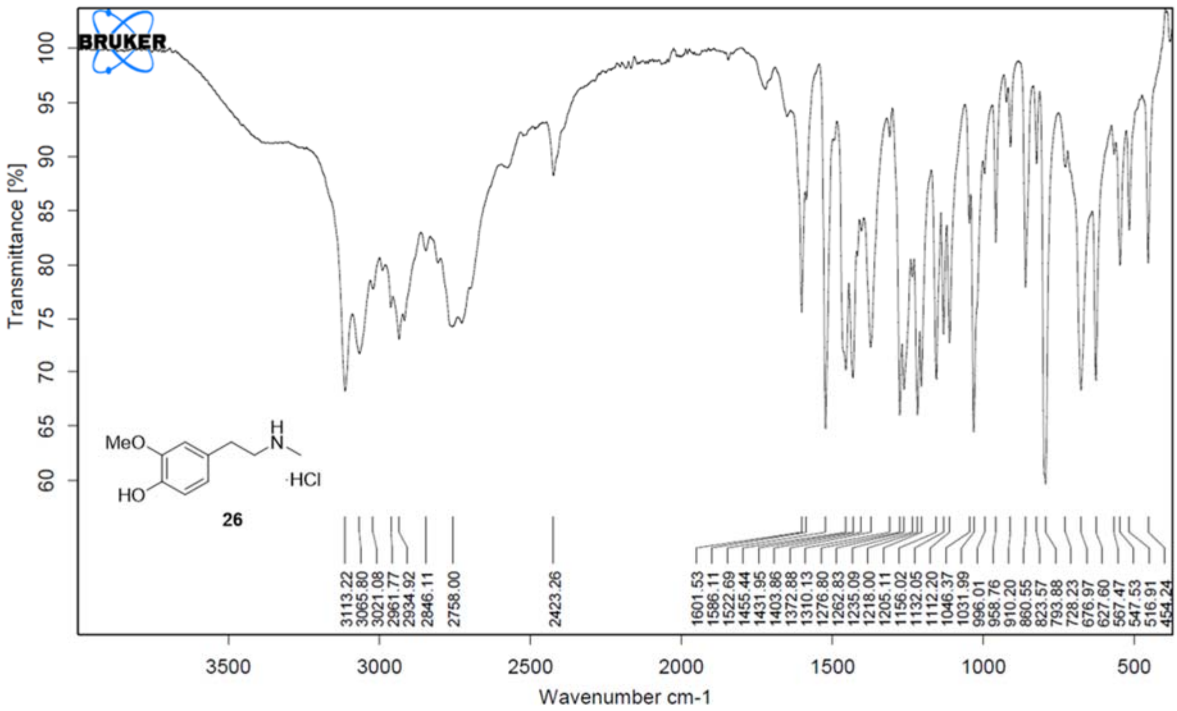

Figure S38: ATR-IR spectrum of 2-methoxy-4-(2-(methylamino)ethyl)phenol hydrochloride (**26**).

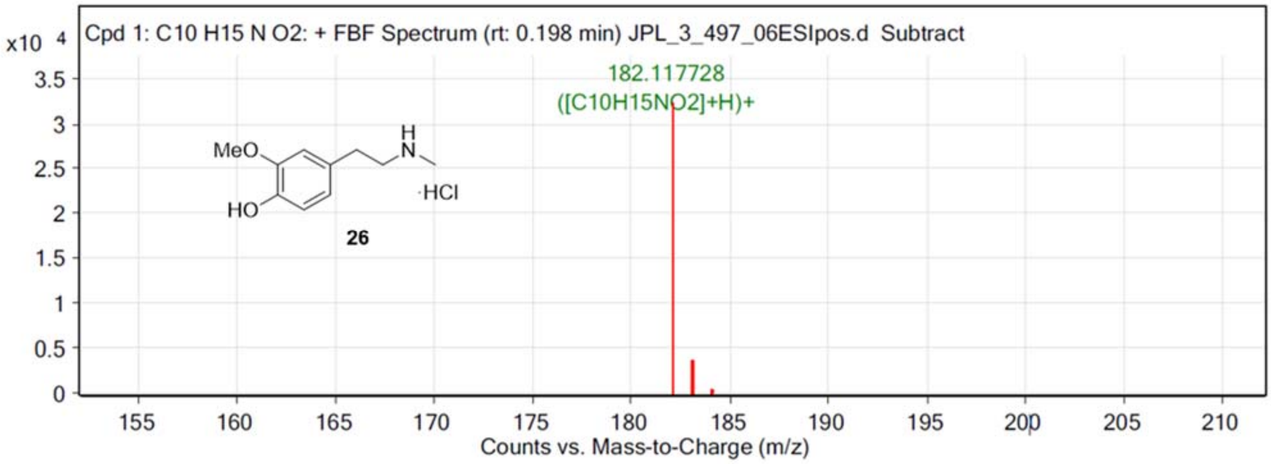

Figure S39: HR-MS spectrum of 2-methoxy-4-(2-(methylamino)ethyl)phenol hydrochloride (**26**).

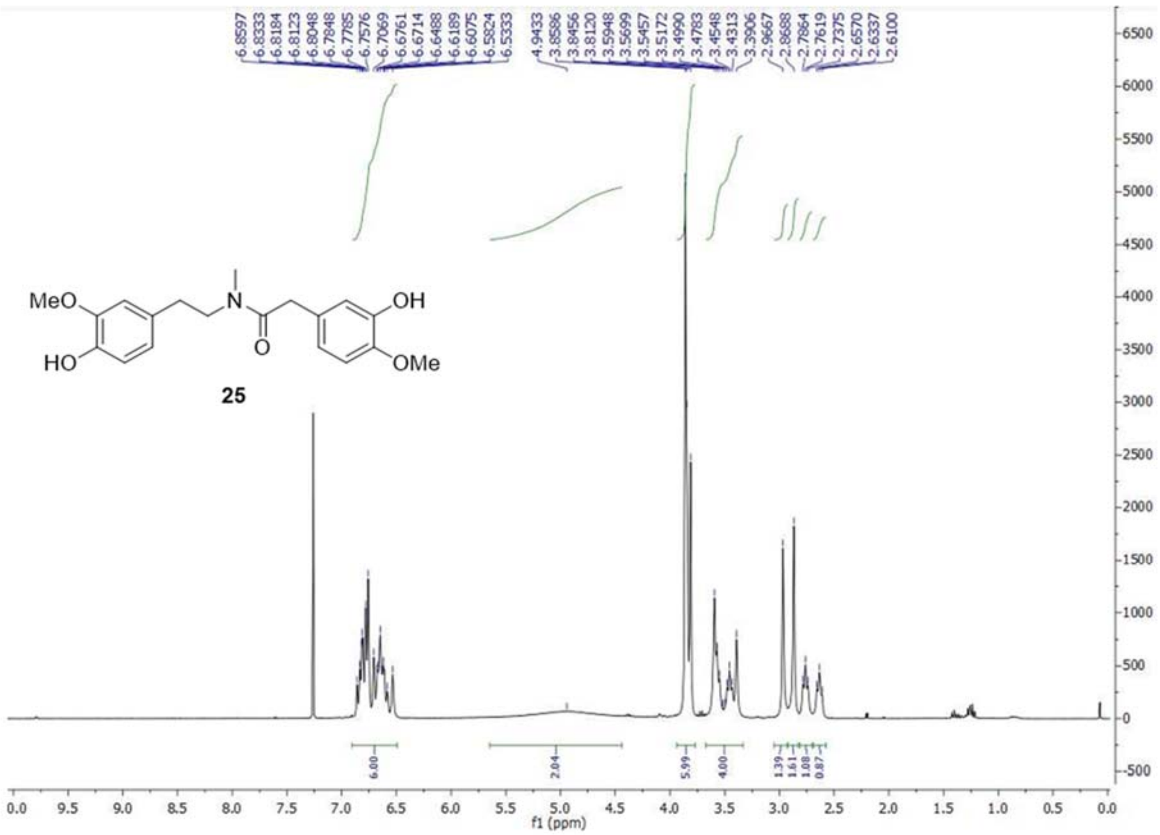

Figure S40: ^{1}H-NMR (300.13 MHz, $CDCl_3$) of *N*-(4-hydroxy-3-methoxyphenethyl)-2-(3-hydroxy-4-methoxyphenyl)-*N*-methylacetamide (**25**).

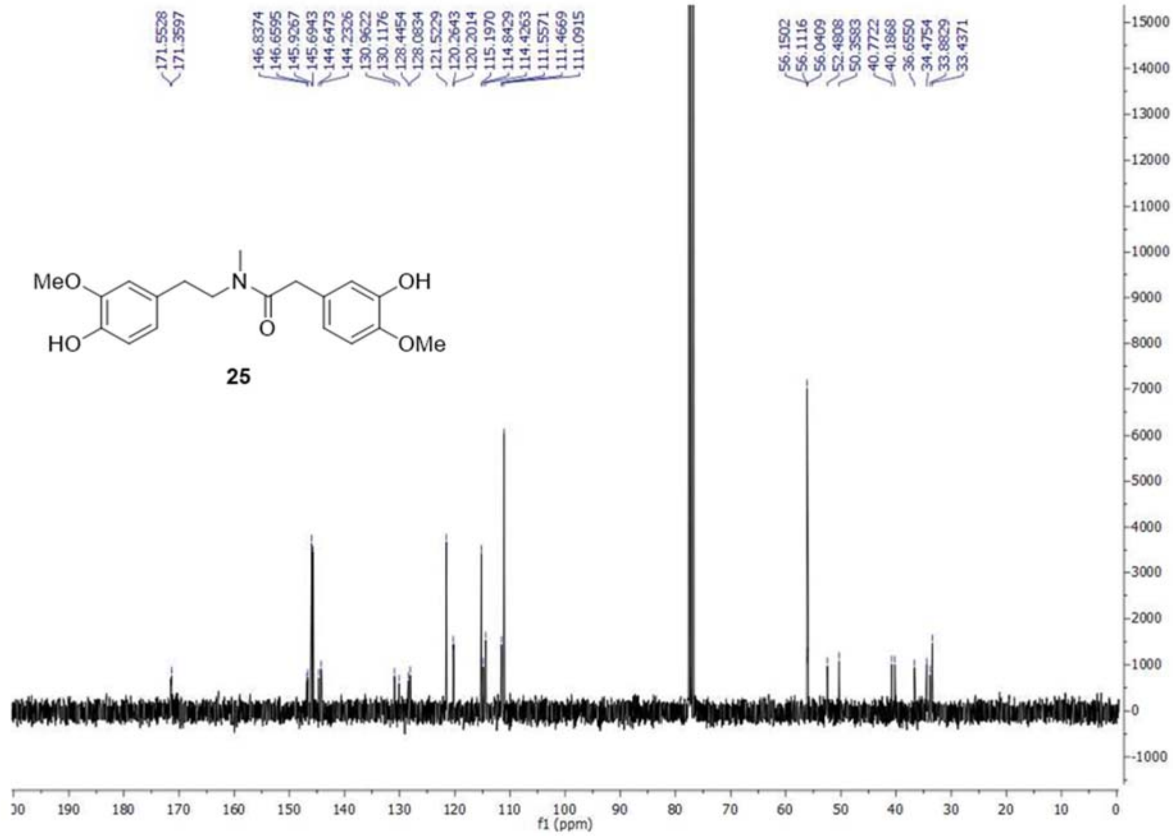

Figure S41: ^{13}C-NMR (75.47 MHz, $CDCl_3$) of *N*-(4-hydroxy-3-methoxyphenethyl)-2-(3-hydroxy-4-methoxyphenyl)-*N*-methylacetamide (**25**).

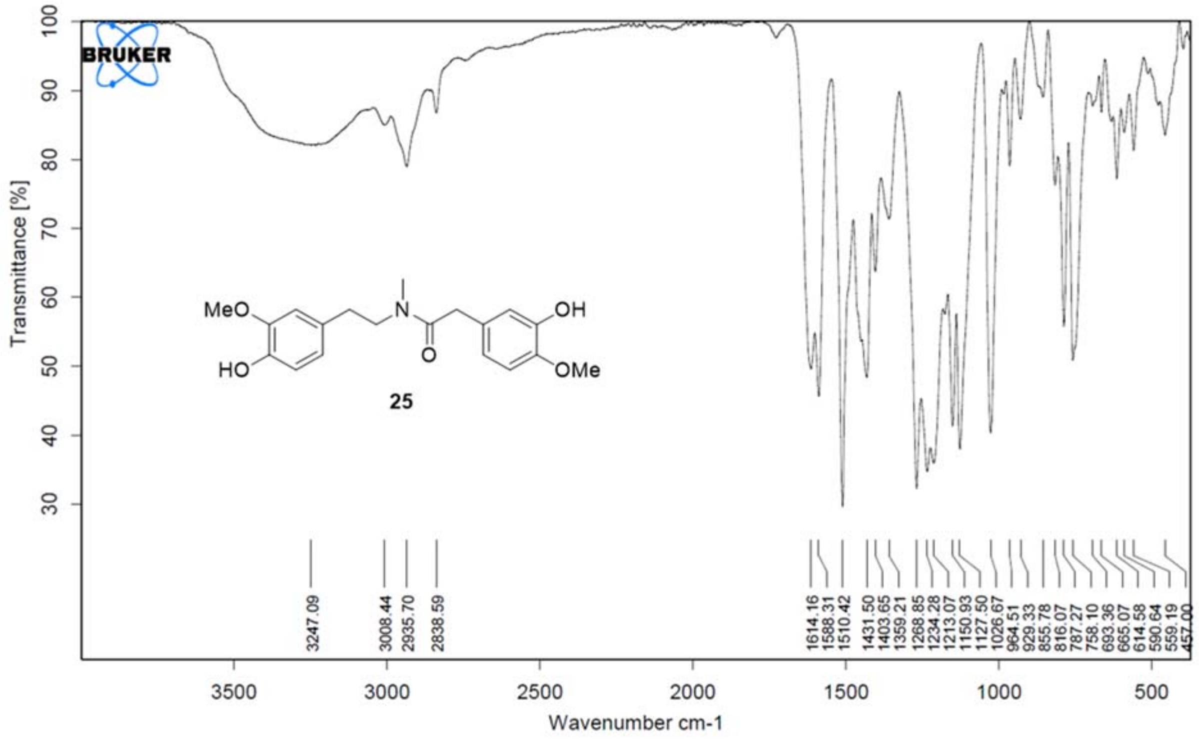

Figure S42: ATR-IR spectrum of *N*-(4-hydroxy-3-methoxyphenethyl)-2-(3-hydroxy-4-methoxyphenyl)-*N*-methylacetamide (**25**).

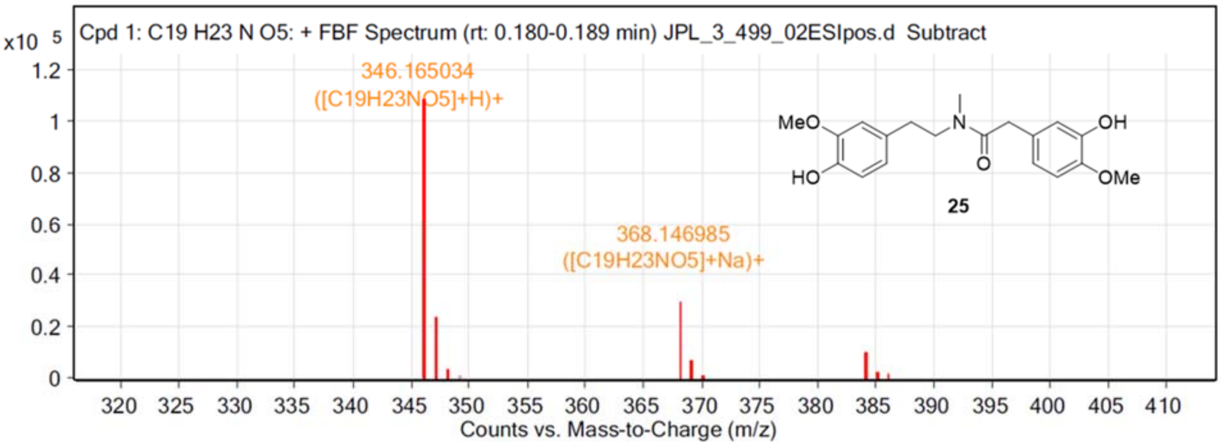

Figure S43: HR-MS spectrum of *N*-(4-hydroxy-3-methoxyphenethyl)-2-(3-hydroxy-4-methoxyphenyl)-*N*-methylacetamide (**25**).

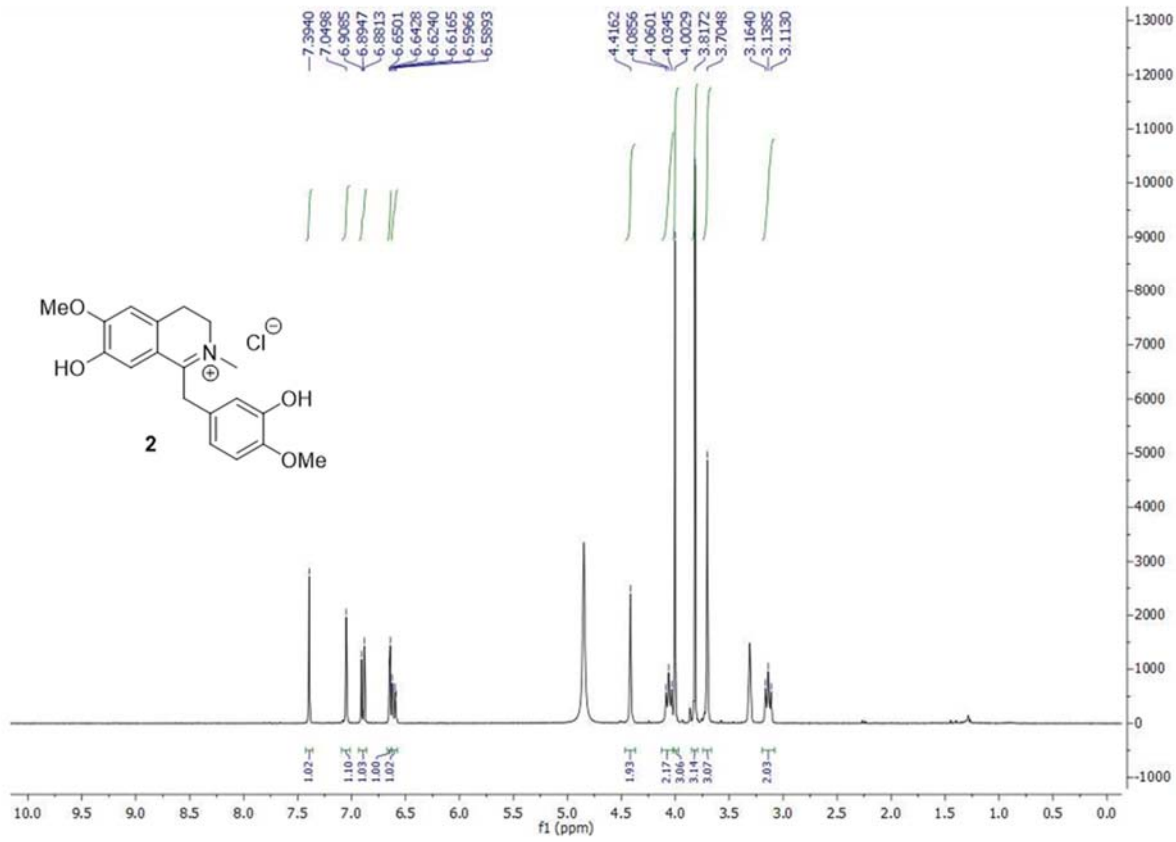

Figure S44: ^{1}H-NMR (300.13 MHz, MeOD-d_4) of 7-hydroxy-1-(3-hydroxy-4-methoxybenzyl)-6-methoxy-2-methyl-3,4-dihydroisoquinolin-2-ium chloride (**2**).

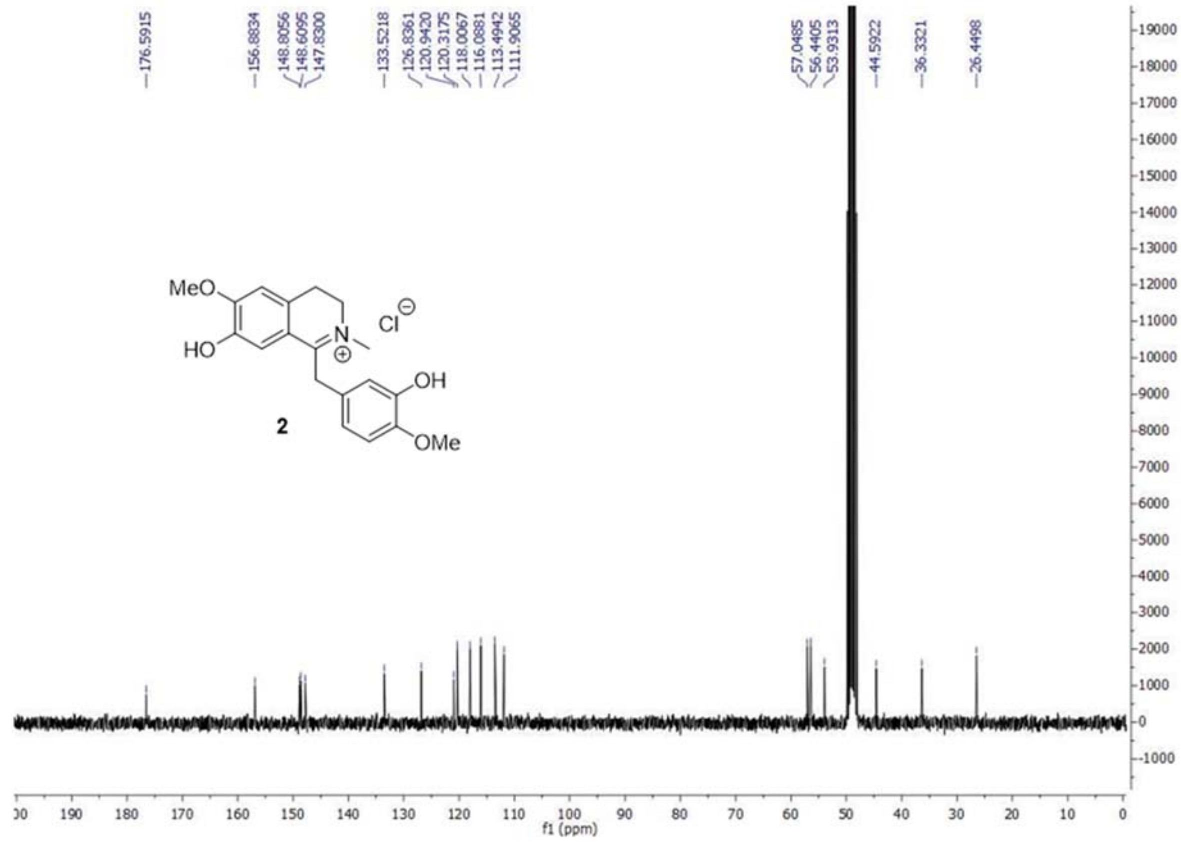

Figure S45: ^{13}C-NMR (75.47 MHz, MeOD-d_4) of 7-hydroxy-1-(3-hydroxy-4-methoxybenzyl)-6-methoxy-2-methyl-3,4-dihydroisoquinolin-2-ium chloride (**2**).

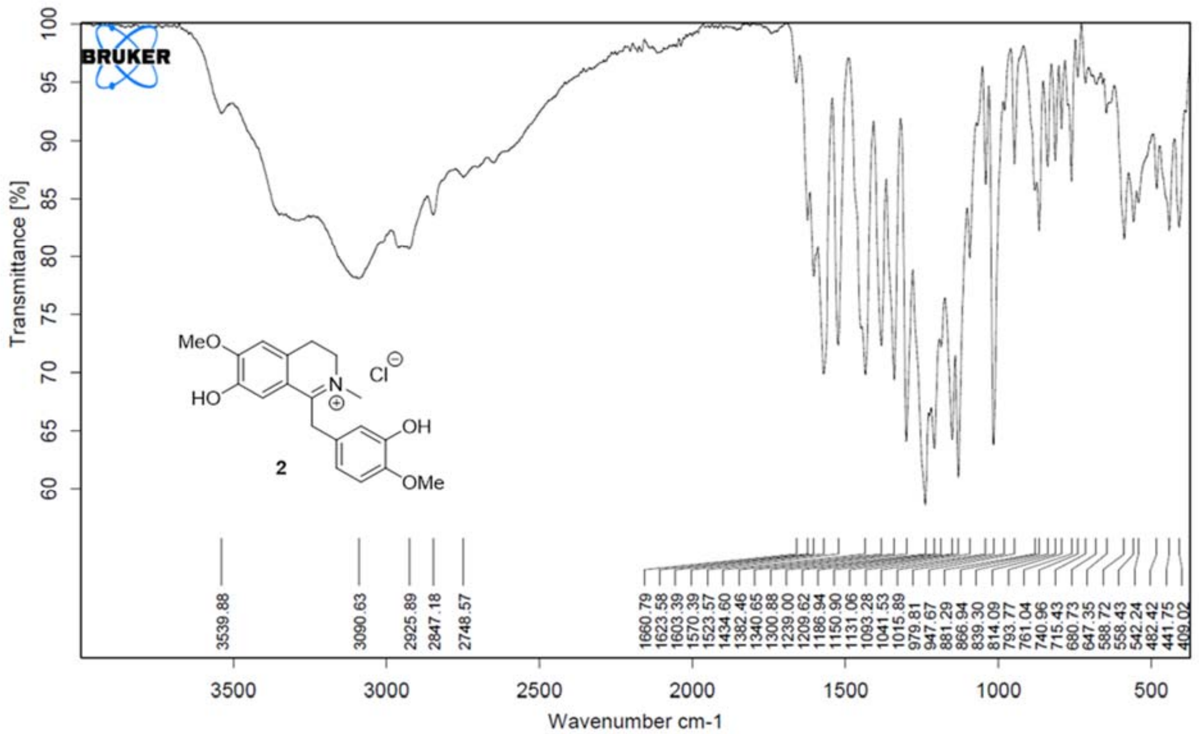

Figure S46: ATR-IR spectrum of 7-hydroxy-1-(3-hydroxy-4-methoxybenzyl)-6-methoxy-2-methyl-3,4-dihydroisoquinolin-2-ium chloride (**2**).

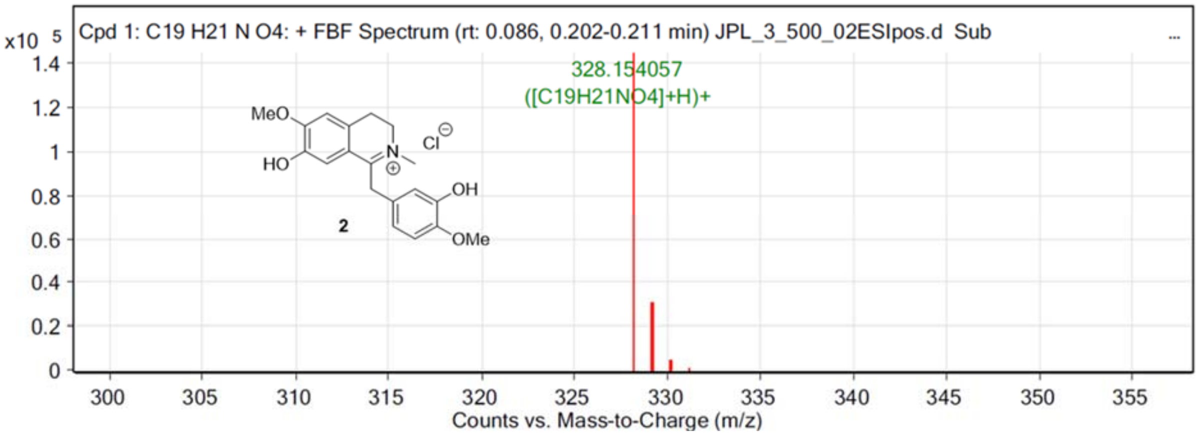

Figure S47: HR-MS spectrum of 7-hydroxy-1-(3-hydroxy-4-methoxybenzyl)-6-methoxy-2-methyl-3,4-dihydroisoquinolin-2-ium chloride (**2**, dehydroreticuline).

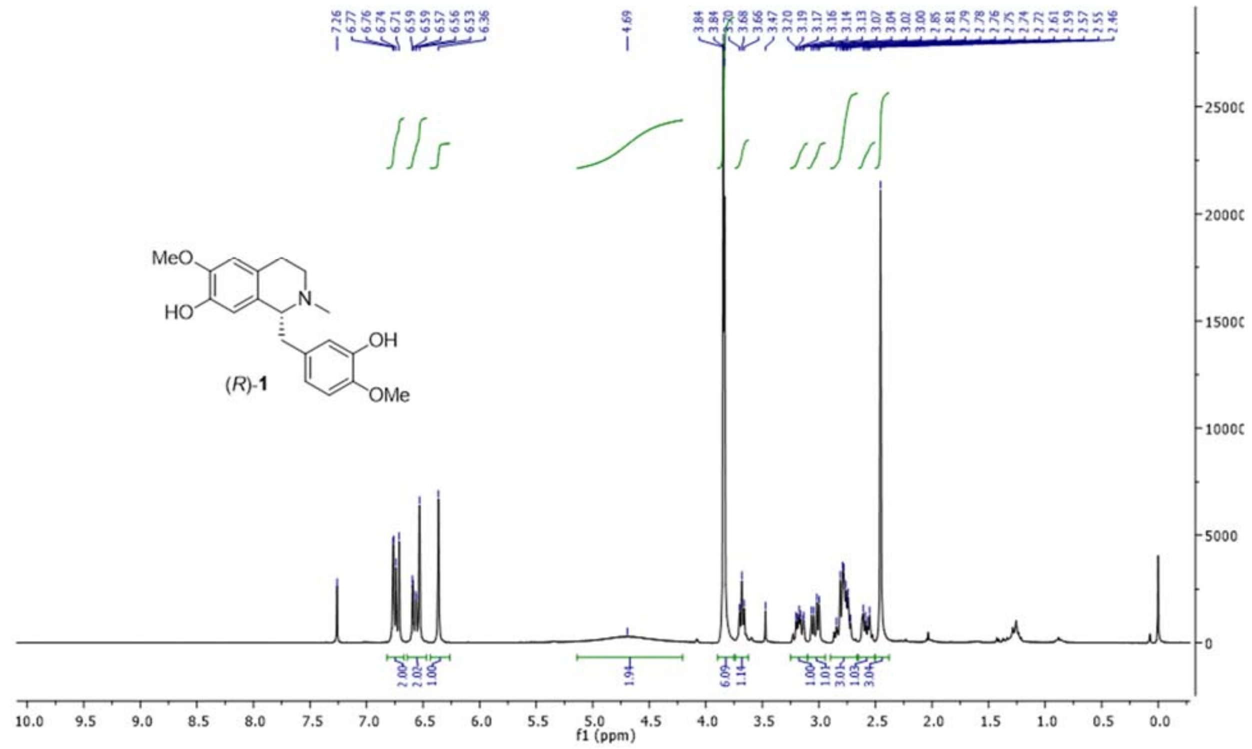

Figure S48: ^{1}H-NMR (300.13 MHz, MeOD-d_4) of (*R*)-1-(3-hydroxy-4-methoxybenzyl)-6-methoxy-2-methyl-1,2,3,4-tetrahydroisoquinolin-7-ol [(*R*)-**1**].

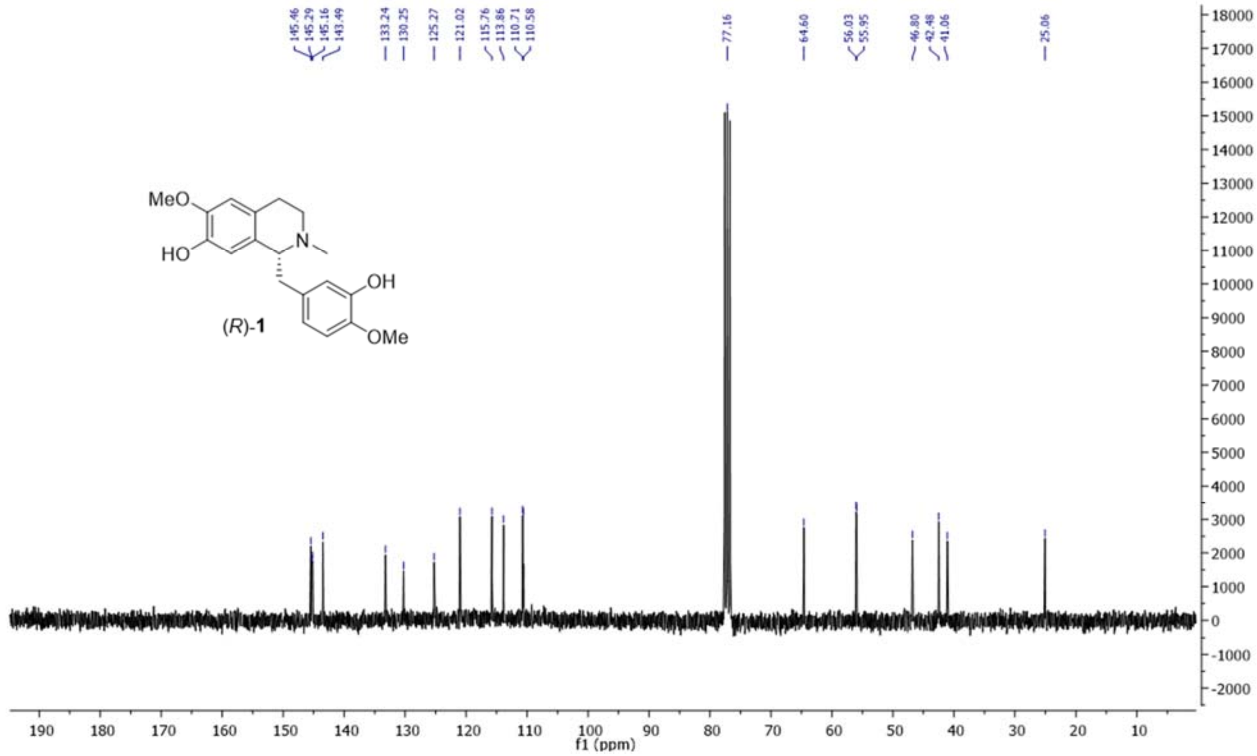

Figure S49: ^{13}C-NMR (75.47 MHz, MeOD-d_4) of (*R*)-1-(3-hydroxy-4-methoxybenzyl)-6-methoxy-2-methyl-1,2,3,4-tetrahydroisoquinolin-7-ol [(*R*)-**1**].

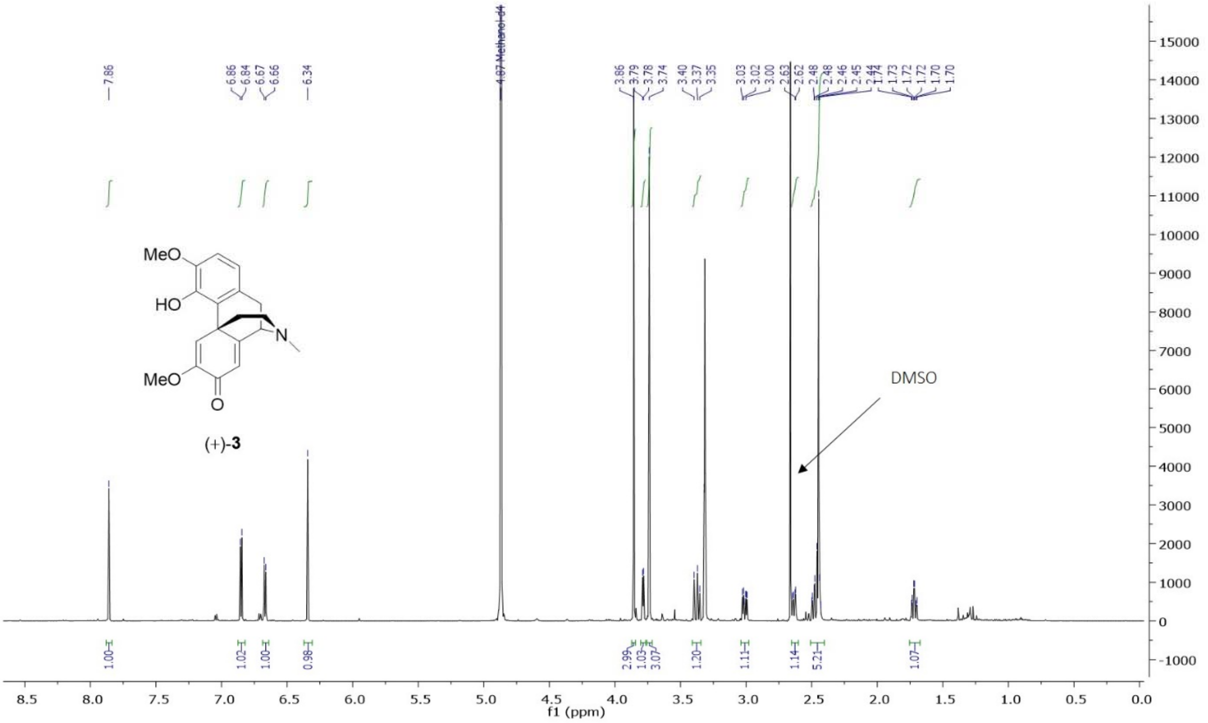

Figure S50: ^{1}H-NMR (700.44 MHz, MeOD-d$_4$) of salutaridine [(+)-**3**].

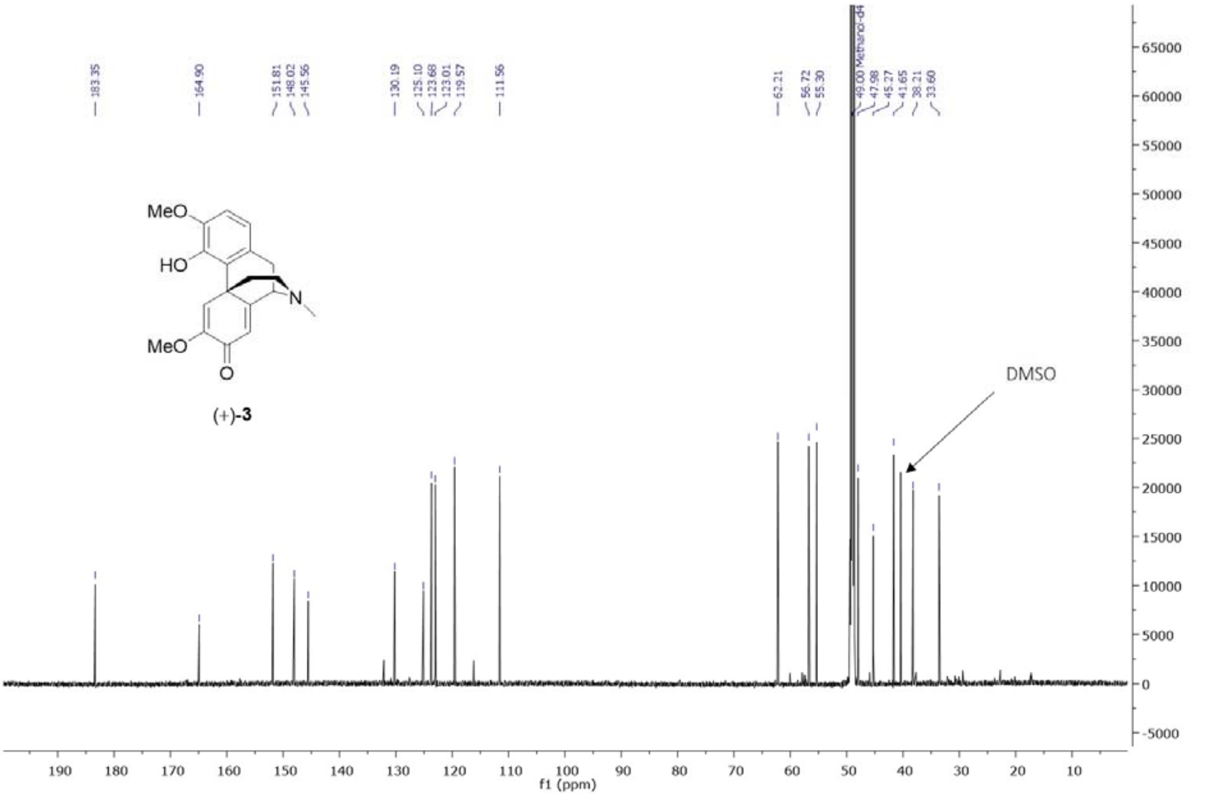

Figure S51: ^{13}C-NMR (175.11 MHz, MeOD-d$_4$) of salutaridine [(+)-**3**].

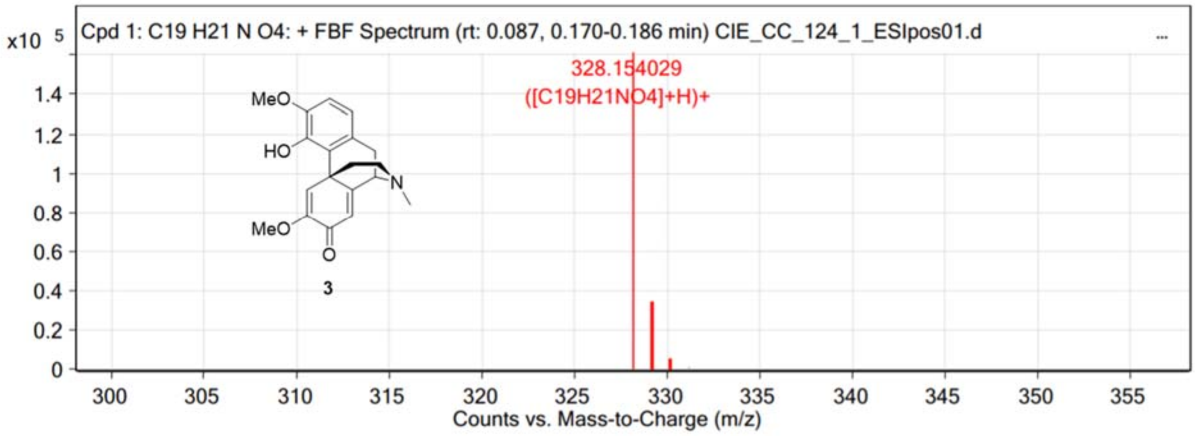

Figure S52: HR-MS spectrum of 4-Hydroxy-3,6-dimethoxy-17-methyl-5,6,8,14-tetradehydromorphinan-7-one [(+)-**3**].

6 Protein sequences

PsDRR

MESSGVPVITLGSGKVMPVLGMGTFEKVGKGSERERLAILKAIEVGYRYFDTAAAYETEEVLGEAIAEALQLGLVKSRDELFISSM
LWCTDAHADRVLLALQNSLRNLKLEYVDLYMLPFPASLKPGKITMDIPEEDICRMDYRSVWAAMEECQNLGFTKSIGVSNFSC
KKLQELMATANIPPAVNQVEMSPAFQQKKLREYCNANNILVSAISVLGSNGTPWGSNAVLGSEVLKKIAMAKGKSVAQVSMR
WVYEQGASLVVKSFSEERLRENLNIFDWELTKEDHEKIGEIPQCRILSAYFLVSPNGPFKSQEELWDDEA

Ntrunc-SalSyn

MGMKISFYQSANTTEWPAGPKTLPIIGNLHQLGGGVPLQVALANLAKVYGGAFTIWIGSWVPMIVISDIDNAREVLVNKSADY
SARDVPDILKIITANGKNIADCDSGPFWHNLKKGLQSCINPSNVMSLSRLQEKDMQNLIKSMQERASQHNGIIKPLDHAKEAS
MRLLSRVIFGHDFSNEDLVIGVKDALDEMVRISGLASLADAFKIAKYLPSQRKNIRDMYATRDRVYNLIQPHIVPNLPENSFLHFL
TSQDYSDEIIYSMVLEIFGLGVDSTAATAVWALSFLVGEQEIQEKLYREINNRTGGQRPVKVVDLKELPYLQAVMKETLRMKPIA
PLAVPHVAAKDTTFKGRRIVKGTKVMVNLYAIHHDPNVFPAPYKFMPERFLKDVNSDGRFGDINTMESSLIPFGAGMRICGGV
ELAKQMVAFALASMVNEFKWDCVSEGKLPDLSEAISFILYMKNPLEAKITPRTKPFRQ

Nswap-SalSyn

MGMKKTSSKGRKISFYQSANTTEWPAGPKTLPIIGNLHQLGGGVPLQVALANLAKVYGGAFTIWIGSWVPMIVISDIDNAREV
LVNKSADYSARDVPDILKIITANGKNIADCDSGPFWHNLKKGLQSCINPSNVMSLSRLQEKDMQNLIKSMQERASQHNGIIKPL
DHAKEASMRLLSRVIFGHDFSNEDLVIGVKDALDEMVRISGLASLADAFKIAKYLPSQRKNIRDMYATRDRVYNLIQPHIVPNLP
ENSFLHFLTSQDYSDEIIYSMVLEIFGLGVDSTAATAVWALSFLVGEQEIQEKLYREINNRTGGQRPVKVVDLKELPYLQAVMKE
TLRMKPIAPLAVPHVAAKDTTFKGRRIVKGTKVMVNLYAIHHDPNVFPAPYKFMPERFLKDVNSDGRFGDINTMESSLIPFGAG
MRICGGVELAKQMVAFALASMVNEFKWDCVSEGKLPDLSEAISFILYMKNPLEAKITPRTKPFRQ

AtR2

MGMSSSSSSSTSMIDLMAAIIKGEPVIVSDPANASAYESVAAELSSMLIENRQFAMIVTTSIAVLIGCIVMLVWRRSGSGNSKRV
EPLKPLVIKPREEEIDDGRKKVTIFFGTQTGTAEGFAKALGEEAKARYEKTRFKIVDLDDYAADDDEYEEKLKKEDVAFFFLATYGD
GEPTDNAARFYKWFTEGNDRGEWLKNLKYGVFGLGNRQYEHFNKVAKVVDDILVEQGAQRLVQVGLGDDDQCIEDDFTAW
REALWPELDTILREEGDTAVATPYTAAVLEYRVSIHDSEDAKFNDINMANGNGYTVFDAQHPYKANVAVKRELHTPESDRSCIH
LEFDIAGSGLTYETGDHVGVLCDNLSETVDEALRLLDMSPDTYFSLHAEKEDGTPISSSLPPPFPPCNLRTALTRYACLLSSPKKSAL
VALAAHASDPTEAERLKHLASPAGKDEYSKWVVESQRSLLEVMAEFPSAKPPLGVFFAGVAPRLQPRFYSISSSPKIAETRIHVTC
ALVYEKMPTGRIHKGVCSTWMKNAVPYEKSENCSSAPIFVRQSNFKLPSDSKVPIIMIGPGTGLAPFRGFLQERLALVESGVELG

PSVLFFGCRNRRMDFIYEEELQRFVESGALAELSVAFSREGPTKEYVQHKMMDKASDIWNMISQGAYLYVCGDAKGMARDV
HRSLHTIAQEQGSMDSTKAEGFVKNLQTSGRYLRDVW

7 DNA sequences

PsDRR

agggacccgggtaccAGATGGAGAGTAGTGGTGTACCAGTAATCACTCTGGGCTCGGGCAAGGTGATGCCTGTTCTGGGCAT
GGGAACATTTGAGAAAGTTGGTAAAGGGTCCGAAAGAGAGAGGTTGGCGATTTTAAAAGCGATAGAGGTGGGTTACAG
ATACTTCGATACAGCTGCTGCATACGAAACTGAAGAGGTTCTTGGAGAAGCTATTGCTGAAGCACTTCAACTTGGCCTAGT
CAAATCTCGAGATGAACTTTTCATCAGTTCCATGCTCTGGTGCACTGATGCTCACGCTGATCGTGTCCTCCTCGCTCTTCAG
AATTCGCTGAGGAATCTTAAATTGGAGTATGTGGATCTATATATGTTACCCTTCCCGGCAAGCTTGAAGCCTGGGAAGATA
ACGATGGACATACCAGAGGAAGATATTTGTCGCATGGACTACAGGTCTGTATGGGCAGCCATGGAAGAGTGTCAAAACC
TTGGCTTCACTAAATCAATCGGTGTTAGCAATTTCTCCTGCAAAAAGCTTCAGGAATTGATGGCGACCGCCAACATCCCTC
CAGCTGTGAATCAAGTGGAGATGAGCCCGGCTTTCCAACAAAAGAAGCTGAGAGAGTATTGCAACGCAAATAATATATTA
GTCAGTGCAATCTCTGTACTGGGATCAAACGGAACCCCATGGGGCTCCAATGCAGTTTTGGGTTCTGAGGTGCTTAAGAA
AATTGCTATGGCCAAAGGAAAATCTGTTGCTCAGGTTAGTATGAGATGGGTTTACGAGCAAGGCGCGAGTCTTGTGGTAA
AAAGTTTCAGTGAAGAGAGATTGAGGGAAAACTTGAACATATTTGACTGGGAACTCACTAAGGAAGACCATGAAAAGAT
CGGTGAGATTCCACAGTGCAGAATCTTGAGTGCTTATTTTTTGGTCTCACCTAATGGACCTTTCAAATCTCAAGAAGAGTT
GTGGGATGATGAAGCTTGAgagctcgctctggtg

Ntrunc-SalSyn

aggagatataccATGGGAATGAAGATCAGTTTCTACCAGAGCGCAAATACCACCGAATGGCCGGCCGGTCCGAAAACCCTGC
CGATTATTGGTAATCTGCATCAGCTGGGCGGCGGCGTTCCGCTGCAGGTTGCTCTGGCAAATCTGGCCAAAGTTTATGGC
GGTGCCTTTACCATTTGGATTGGTAGCTGGGTTCCGATGATTGTTATTAGTGATATTGATAACGCCCGTGAAGTTCTGGTT
AATAAGAGCGCAGATTATAGCGCACGCGATGTGCCGGATATTCTGAAAATTATTACCGCCAATGGTAAAAATATCGCCGA
TTGCGATAGCGGCCCGTTTTGGCATAATCTGAAAAAAGGCCTGCAGAGCTGCATTAATCCGAGTAATGTTATGAGCCTGA
GTCGCCTGCAGGAAAAAGATATGCAGAATCTGATTAAGAGCATGCAGGAACGCGCAAGCCAGCATAATGGCATTATTAA
GCCGCTGGATCATGCAAAAGAAGCAAGCATGCGTCTGCTGAGTCGTGTGATTTTTGGCCATGATTTTAGCAATGAAGATC
TGGTTATTGGCGTGAAAGATGCCCTGGATGAAATGGTTCGTATTAGCGGCCTGGCAAGCCTGGCAGATGCATTCAAAATT
GCCAAATATCTGCCGAGTCAGCGCAAAAATATTCGTGATATGTATGCCACCCGTGATCGTGTGTATAATCTGATTCAGCCG
CATATTGTTCCGAATCTGCCGGAAAATAGTTTTCTGCATTTTCTGACCAGCCAGGATTATAGTGATGAAATTATCTATAGCA
TGGTGCTGGAAATTTTTGGTCTGGGTGTTGATAGTACCGCAGCAACCGCAGTGTGGGCCCTGAGTTTTCTGGTGGGCGAA
CAGGAAATTCAGGAAAAACTGTATCGCGAAATTAATAATCGCACCGGTGGTCAGCGTCCGGTTAAAGTTGTGGATCTGAA
AGAACTGCCGTATCTGCAGGCAGTGATGAAAGAAACCCTGCGCATGAAACCGATTGCACCGCTGGCCGTTCCGCATGTGG
CCGCAAAAGATACCACCTTTAAAGGTCGCCGCATTGTTAAAGGCACCAAAGTGATGGTTAATCTGTATGCCATTCATCATG
ATCCGAATGTTTTTCCGGCACCGTATAAATTCATGCCGGAACGTTTTCTGAAAGATGTGAATAGCGATGGCCGCTTTGGCG
ATATTAATACAATGGAAAGCAGCCTGATTCCGTTTGGTGCCGGTATGCGTATTTGTGGCGGTGTGGAACTGGCAAAACAG
ATGGTTGCATTTGCACTGGCCAGTATGGTTAATGAATTCAAATGGGATTGTGTGAGCGAAGGCAAACTGCCGGATCTGAG
TGAAGCCATTAGCTTTATTCTGTATATGAAAAACCCGCTGGAAGCCAAAATTACCCCGCGCACCAAACCGTTTCGTCAGCT
CGAGCACCACCACCACCACCACtgagat

Nswap-SalSyn

gatataccATGGGAATGAAGAAAACGAGCAGTAAAGGCCGTAAGATCAGTTTCTACCAGAGCGCAAATACCACCGAATGGC
CGGCCGGTCCGAAAACCCTGCCGATTATTGGTAATCTGCATCAGCTGGGCGGCGGCGTTCCGCTGCAGGTTGCTCTGGCA
AATCTGGCCAAAGTTTATGGCGGTGCCTTTACCATTTGGATTGGTAGCTGGGTTCCGATGATTGTTATTAGTGATATTGAT
AACGCCCGTGAAGTTCTGGTTAATAAGAGCGCAGATTATAGCGCACGCGATGTGCCGGATATTCTGAAAATTATTACCGC
CAATGGTAAAAATATCGCCGATTGCGATAGCGGCCCGTTTTGGCATAATCTGAAAAAAGGCCTGCAGAGCTGCATTAATC
CGAGTAATGTTATGAGCCTGAGTCGCCTGCAGGAAAAAGATATGCAGAATCTGATTAAGAGCATGCAGGAACGCGCAAG

CCAGCATAATGGCATTATTAAGCCGCTGGATCATGCAAAAGAAGCAAGCATGCGTCTGCTGAGTCGTGTGATTTTTGGCC
ATGATTTTAGCAATGAAGATCTGGTTATTGGCGTGAAAGATGCCCTGGATGAAATGGTTCGTATTAGCGGCCTGGCAAGC
CTGGCAGATGCATTCAAAATTGCCAAATATCTGCCGAGTCAGCGCAAAAATATTCGTGATATGTATGCCACCCGTGATCGT
GTGTATAATCTGATTCAGCCGCATATTGTTCCGAATCTGCCGGAAAATAGTTTTCTGCATTTTCTGACCAGCCAGGATTATA
GTGATGAAATTATCTATAGCATGGTGCTGGAAATTTTTGGTCTGGGTGTTGATAGTACCGCAGCAACCGCAGTGTGGGCC
CTGAGTTTTCTGGTGGGCGAACAGGAAATTCAGGAAAAACTGTATCGCGAAATTAATAATCGCACCGGTGGTCAGCGTCC
GGTTAAAGTTGTGGATCTGAAAGAACTGCCGTATCTGCAGGCAGTGATGAAAGAAACCCTGCGCATGAAACCGATTGCA
CCGCTGGCCGTTCCGCATGTGGCCGCAAAAGATACCACCTTTAAAGGTCGCCGCATTGTTAAAGGCACCAAAGTGATGGT
TAATCTGTATGCCATTCATCATGATCCGAATGTTTTTCCGGCACCGTATAAATTCATGCCGGAACGTTTTCTGAAAGATGTG
AATAGCGATGGCCGCTTTGGCGATATTAATACAATGGAAAGCAGCCTGATTCCGTTTGGTGCCGGTATGCGTATTTGTGG
CGGTGTGGAACTGGCAAAACAGATGGTTGCATTTGCACTGGCCAGTATGGTTAATGAATTCAAATGGGATTGTGTGAGC
GAAGGCAAACTGCCGGATCTGAGTGAAGCCATTAGCTTTATTCTGTATATGAAAAACCCGCTGGAAGCCAAAATTACCCC
GCGCACCAAACCGTTTCGTCAGCTCGAGCACCACCACCACCACCACtgag

AtR2

gatataccATGGGAATGAGTAGCAGCAGTAGTAGTAGTACCAGCATGATTGATCTGATGGCCGCAATTATTAAGGGTGAAC
CGGTTATTGTGAGCGATCCGGCCAATGCCAGTGCCTATGAAAGTGTGGCCGCAGAACTGAGTAGCATGCTGATTGAAAAT
CGCCAGTTTGCAATGATTGTGACCACCAGTATTGCAGTGCTGATTGGCTGTATTGTTATGCTGGTTTGGCGTCGTAGTGGT
AGCGGCAATAGTAAACGTGTTGAACCGCTGAAACCGCTGGTTATTAAGCCGCGCGAAGAAGAAATTGATGATGGTCGCA
AAAAAGTGACCATTTTCTTTGGTACCCAGACCGGCACCGCCGAAGGCTTTGCAAAAGCACTGGGTGAAGAAGCAAAAGC
ACGTTATGAAAAAACCCGCTTTAAAATTGTGGATCTGGATGATTATGCAGCCGATGATGATGAATATGAAGAAAAACTGA
AGAAGGAAGATGTGGCCTTTTTCTTTCTGGCCACCTATGGTGACGGTGAACCGACCGATAATGCCGCCCGCTTTTATAAAT
GGTTTACCGAAGGTAATGACCGTGGTGAATGGCTGAAAAATCTGAAATATGGTGTGTTTGGTCTGGGTAATCGCCAGTAT
GAACATTTTAATAAGGTGGCAAAAGTGGTTGATGATATTCTGGTGGAACAGGGCGCCCAGCGCCTGGTTCAGGTTGGCCT
GGGCGATGATGATCAGTGCATTGAAGATGATTTTACCGCCTGGCGCGAAGCACTGTGGCCGGAACTGGATACCATTCTGC
GCGAAGAAGGTGACACCGCAGTGGCAACCCCGTATACCGCAGCCGTTCTGGAATATCGTGTGAGCATTCATGATAGTGA
AGATGCCAAATTCAATGATATTAACATGGCAAATGGCAATGGTTATACCGTTTTTGATGCACAGCATCCGTATAAAGCCAA
TGTGGCCGTTAAACGTGAACTGCATACCCCGGAAAGTGATCGTAGCTGCATTCATCTGGAATTTGATATTGCAGGTAGTG
GCCTGACCTATGAAACCGGTGACCATGTGGGTGTTCTGTGTGATAATCTGAGTGAAACCGTGGATGAAGCACTGCGCCTG
CTGGATATGAGCCCGGATACCTATTTTAGCCTGCATGCCGAAAAAGAAGATGGCACCCCGATTAGCAGTAGTCTGCCGCC
GCCGTTTCCGCCGTGCAATCTGCGCACCGCACTGACCCGTTATGCATGCCTGCTGAGCAGCCCGAAAAAATCAGCCCTGG
TGGCCCTGGCAGCCCATGCCTCAGATCCGACCGAAGCCGAACGCCTGAAACATCTGGCAAGTCCGGCCGGCAAAGATGA
ATATAGCAAATGGGTGGTTGAAAGCCAGCGCAGCCTGCTGGAAGTGATGGCCGAATTTCCGAGCGCAAAACCGCCGCTG
GGCGTTTTCTTTGCAGGCGTTGCCCCGCGTCTGCAGCCGAGATTTTATAGCATTAGTAGCAGCCCGAAGATTGCAGAAAC
CCGCATTCATGTGACCTGTGCACTGGTGTATGAAAAAATGCCGACCGGTCGTATTCATAAAGGTGTTTGTAGTACCTGGAT
GAAAAATGCAGTGCCGTATGAAAAAAGCGAAAATTGTAGCAGCGCACCGATTTTTGTGCGCCAGAGCAATTTTAAACTGC
CGAGCGATAGCAAAGTTCCGATTATTATGATTGGTCCGGGTACCGGTCTGGCACCGTTTCGTGGCTTTCTGCAGGAACGC
CTGGCCCTGGTGGAAAGTGGTGTGGAACTGGGCCCGAGTGTGCTGTTTTTCGGCTGCCGCAATCGCCGTATGGATTTTAT
CTATGAAGAAGAACTGCAGCGCTTTGTTGAAAGCGGTGCCCTGGCCGAACTGAGTGTTGCCTTTAGTCGCGAAGGTCCGA
CCAAAGAATATGTTCAGCATAAAATGATGGACAAAGCCAGTGATATTTGGAATATGATTAGCCAGGGTGCCTATCTGTAT
GTTTGCGGCGATGCCAAAGGTATGGCACGTGATGTGCATCGCAGTCTGCATACCATTGCACAGGAACAGGGCAGCATGG
ATAGTACCAAAGCCGAAGGTTTTGTGAAAAATCTGCAGACCAGTGGCCGCTATCTGCGCGATGTGTGGCTCGAGTCTGGT
AAAGAAACCGCTGCTGCGAAATTTGAACGCCAGCACATGGACTCGTCTACTAGCGCAGCTtaat

8 Primers

Nswap-SalSyn

Alteration site	Forward primer (5' to 3')	Reverse primer (5' to 3')
N-terminus of Ntrunc-SalSyn (KKTSSKGR was inserted)	AAACGAGCAGTAAAGGCCGTAAGATCAGTTTCTACCAG	CCTTTACTGCTCGTTTTCTTCATTCCCATGGTATATCTC

9 References

[1] S. Velikogne, V. Resch, C. Dertnig, J. H. Schrittwieser, W. Kroutil, *ChemCatChem* **2018**, 10, 3236-3246.

[2] V. I. Tishkov, A. G. Galkin, G. N. Marchenko, O. A. Egorova, D. V. Sheluho, L. B. Kulakova, L. A. Dementieva, A. M. Egorov, *Biochem. Bioph. Res. Co.* **1993**, 192, 976-981.

[3] M. Ihara, Y. Kawano, M. Urano, A. Okabe, *PLoS ONE* **2013**, 8, e71581.

[4] T. W. Johannes, R. D. Woodyer, H. Zhao, *Appl. Environ. Microbiol.* **2005**, 71, 5728-5734.

[5] M. J. McLachlan, T. W. Johannes, H. Zhao, *Biotechnol. Bioeng.* **2008**, 99, 268-274.

[6] R. J. Mattson, K. M. Pham, D. J. Leuck, K. A. Cowen, *J. Org. Chem*. **1990**, 55, 2552-2554.

[7] S. Bhattacharyya, *J. Org. Chem*. **1995**, 60, 4928-4929.

[8] E. Podyacheva, O. I. Afanasyev, A. A. Tsygankov, M. Makarova, D. Chusov, *Synthesis* **2019**, 51, 2667-2677.

[9] S. C. Farrow, J. M. Hagel, G. A. Beaudoin, D. C. Burns, P. J. Facchini, *Nat. Chem. Biol*. **2015**, 11, 728-732.

[10] A. Nakagawa, E. Matsumura, T. Koyanagi, T. Katayama, N. Kawano, K. Yoshimatsu, K. Yamamoto, H. Kumagai, F. Sato, H. Minami, *Nat. Commun.* **2016**, 7, 10390.

[11] V. Bhatt, S. Kumari, P. Upadhyay, P. Agrawal, Anmol, D. Sahalb, U. Sharma, *J. Ethnopharmacol.* **2020**, 262, 113185.
